# Vitamin Biosynthesis by Human Gut Butyrate-Producing Bacteria and Cross-Feeding in Synthetic Microbial Communities

**DOI:** 10.1128/mBio.00886-20

**Published:** 2020-07-14

**Authors:** Eva C. Soto-Martin, Ines Warnke, Freda M. Farquharson, Marilena Christodoulou, Graham Horgan, Muriel Derrien, Jean-Michel Faurie, Harry J. Flint, Sylvia H. Duncan, Petra Louis

**Affiliations:** a The Rowett Institute, University of Aberdeen, Foresterhill, Aberdeen, United Kingdom; b Biomathematics & Statistics Scotland, Aberdeen, United Kingdom; c Danone Nutricia Research, Palaiseau, Paris, France; VA Palo Alto Health Care System

**Keywords:** amino acid biosynthesis, butyrate, cross-feeding, human gut microbiota, vitamin biosynthesis

## Abstract

We investigated the requirement of 15 human butyrate-producing gut bacterial strains for eight B vitamins and the proteinogenic amino acids by a combination of genome sequence analysis and *in vitro* growth experiments. The *Ruminococcaceae* species Faecalibacterium prausnitzii and Subdoligranulum variabile were auxotrophic for most of the vitamins and the amino acid tryptophan. Within the *Lachnospiraceae*, most species were prototrophic for all amino acids and several vitamins, but biotin auxotrophy was widespread. In addition, most of the strains belonging to Eubacterium rectale and *Roseburia* spp., but few of the other *Lachnospiraceae* strains, were auxotrophic for thiamine and folate. Synthetic coculture experiments of five thiamine or folate auxotrophic strains with different prototrophic bacteria in the absence and presence of different vitamin concentrations were carried out. This demonstrated that cross-feeding between bacteria does take place and revealed differences in cross-feeding efficiency between prototrophic strains. Vitamin-independent growth stimulation in coculture compared to monococulture was also observed, in particular for F. prausnitzii A2-165, suggesting that it benefits from the provision of other growth factors from community members. The presence of multiple vitamin auxotrophies in the most abundant butyrate-producing *Firmicutes* species found in the healthy human colon indicates that these bacteria depend upon vitamins supplied from the diet or via cross-feeding from other members of the microbial community.

## Abstract (precis)

Microbes in the intestinal tract have a strong influence on human health. Their fermentation of dietary nondigestible carbohydrates leads to the formation of health-promoting short-chain fatty acids, including butyrate, which is the main fuel for the colonic wall and has anticarcinogenic and anti-inflammatory properties. A good understanding of the growth requirements of butyrate-producing bacteria is important for the development of efficient strategies to promote these microbes in the gut, especially in cases where their abundance is altered. The demonstration of the inability of several dominant butyrate producers to grow in the absence of certain vitamins confirms the results of previous *in silico* analyses. Furthermore, establishing that strains prototrophic for thiamine or folate (butyrate producers and non-butyrate producers) were able to stimulate growth and affect the composition of auxotrophic synthetic communities suggests that the provision of prototrophic bacteria that are efficient cross feeders may stimulate butyrate-producing bacteria under certain *in vivo* conditions.

## INTRODUCTION

The human large intestinal microbiota largely receives its energy requirements from dietary carbohydrates that cannot be digested in the upper intestine and reach the colon, which leads to the formation of a range of fermentation products. Butyrate, one of the major short-chain fatty acids (SCFA) produced by the gut microbiota, exerts multiple health benefits, including anti-inflammatory and anticarcinogenic effects (1). Among the phylum *Firmicutes*, in the order *Clostridiales*, several bacterial species are major producers of butyrate in the colon (2, 3). Utilization of nondigestible carbohydrates by butyrate-producing bacteria is well documented (4, 5). However, there is limited information about their requirements for growth factors (nucleotides, amino acids, and vitamins). Bacteria able to produce growth factors are called prototrophs, whereas bacteria that cannot produce them are auxotrophs and need to obtain them from external sources including diet or cross-feeding by other bacteria (6). A recent study explored the nutritional preferences of 96 human gut bacterial strains (7), but data from *Lachnospiraceae* and *Ruminococcaceae* members are still limited.

Vitamins are generally the main organic micronutrients needed for bacterial metabolism, in particular the B vitamins (biotin [B_7_], cobalamin [B_12_], folate [B_9_], nicotinic acid [B_3_], pantothenic acid [B_5_], pyridoxine [B_6_], riboflavin [B_2_], and thiamine [B_1_]). They primarily act as enzymatic cofactors or precursors of cofactors (8). Butyrate production directly depends on the presence of two vitamins: thiamine is a cofactor of pyruvate:ferredoxin 2-oxidoreductase that generates acetyl coenzyme A (acetyl-CoA) from pyruvate (9), and riboflavin is part of the electron transfer flavoprotein complex with butyryl-CoA dehydrogenase that leads to the generation of butyryl-CoA from crotonyl-CoA (10). Vitamin-related cofactors are metabolically expensive to produce; therefore, their final forms or precursors are commonly shared by microbial community members (11–13). Recently, an *in vitro* and *in vivo* study (14) confirmed the contribution of B vitamin exchange and sharing. However, it is still unknown how this applies to butyrate producers. Currently, most of the information on vitamin biosynthesis by gut bacteria has been generated through *in silico* approaches by interrogating genome sequence information, which revealed differences across taxa (15–18). The abundance of certain vitamin pathway genes within the microbiota has been observed to change over the life course of humans. For example, folate synthesis genes seem to be enriched in the gut microbiota of babies, while cobalamin and thiamine biosynthesis genes increase with age (19, 20). Differences in the abundance of certain vitamin biosynthetic genes within the microbiome have also been reported across different human populations (19). Experimental studies that assess the ability of gut bacteria to synthesize vitamins, on the other hand, are much more limited. The existing information is also revealing the limitations of genomic studies to predict metabolic capacities as shown by discrepancies between *in silico* and *in vitro* data (7, 15, 21). Among butyrate-producing bacteria, only the vitamin requirements of Faecalibacterium prausnitzii have been investigated experimentally (21, 22), and it has been hypothesized that F. prausnitzii uses riboflavin as a redox mediator (23).

Amino acid requirements by gut microbiota are also largely unexplored. Studies have addressed amino acid catabolism by bacteria of the human large intestine (reviewed in references 24 to 26), but amino acid production has mainly been investigated in bacterial species from the rumen (27–30). Studies of monogastric animals, including humans, have provided evidence of the contribution of amino acids of microbial origin to the pool of essential amino acids of the animal host (31–33) but do not give details of the species producing and/or excreting them. The study by Heinken et al. (21) is, to our knowledge, the only one assessing amino acid synthesis by a human butyrate-producing bacterium, *F. prausnitzii*, by metabolic modeling and *in vitro* culture.

In this study, the needs for two types of growth factors, eight B vitamins and 20 amino acids, in 15 butyrate-producing gut bacterial strains were explored by combining *in silico* analyses of biosynthetic pathways with *in vitro* growth tests. For two vitamins (folate and thiamine) whose absence resulted in impaired growth in several strains, growth behaviors were established in response to a range of vitamin concentrations spanning those estimated to be present in the colon, and cross-feeding of vitamins between prototrophic and auxotrophic bacteria was demonstrated.

## RESULTS

### Investigation of vitamin and amino acid pathways in microbial genomes.

**FIG 1:**
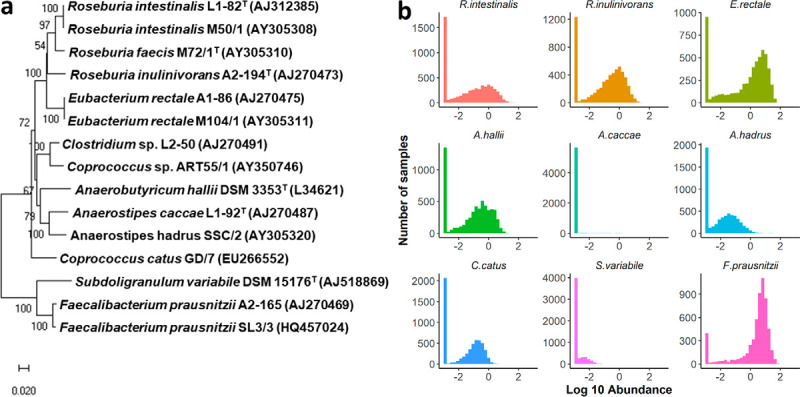
Strains used in this study. (a) 16S rRNA gene-based phylogenetic tree of the 15 butyrate-producing strains of this study. Prealigned sequences (identified by their accession number in brackets) were downloaded from the Ribosomal Database project (http://rdp.cme.msu.edu/index.jsp) (64) and a neighbor-joining tree generated with standard settings with MEGA X (65). The percentage of replicate trees in which the associated taxa clustered together in the bootstrap test (500 replicates) are shown next to the branches. The scale bar represents the number of base substitutions per site. Names provided as per the NCBI taxonomy website (https://www.ncbi.nlm.nih.gov/taxonomy). *Clostridium* sp. L2-50 shares 96% 16S rRNA gene sequence identity with *Coprococcus* sp. ART55/1 and likely belongs to the genus *Coprococcus* (66). (b) Abundance distribution of selected species in human metagenomes in fecal samples. Data were retrieved from curatedMetagenomicData (https://waldronlab.github.io/curatedMetagenomicData/; accessed 2018; *n* = 5,848), which provides uniformly processed human metagenomics data sets (67), showing a variability of distribution. Abundance distributions were visualized with R v 3.5.2. using a logarithmic scale.

The butyrate-producing human gut isolates investigated here comprised ten *Lachnospiraceae* species and two *Ruminococcaceae* species, and for some species, several strains were included to assess whether nutrient requirements differed within species. Based on metagenomic analysis, the strains encompassed both highly prevalent and/or abundant species (e.g., *F. prausnitzii*, Eubacterium rectale) and species not detected in many individuals and/or of low abundance (e.g., Anaerostipes caccae) (Fig. 1). The *in silico* analysis of metabolite pathways comprised the interrogation of several annotated genome databases followed by manual BLASTP analysis for selected genes as detailed in Materials and Methods.

**FIG 2:**
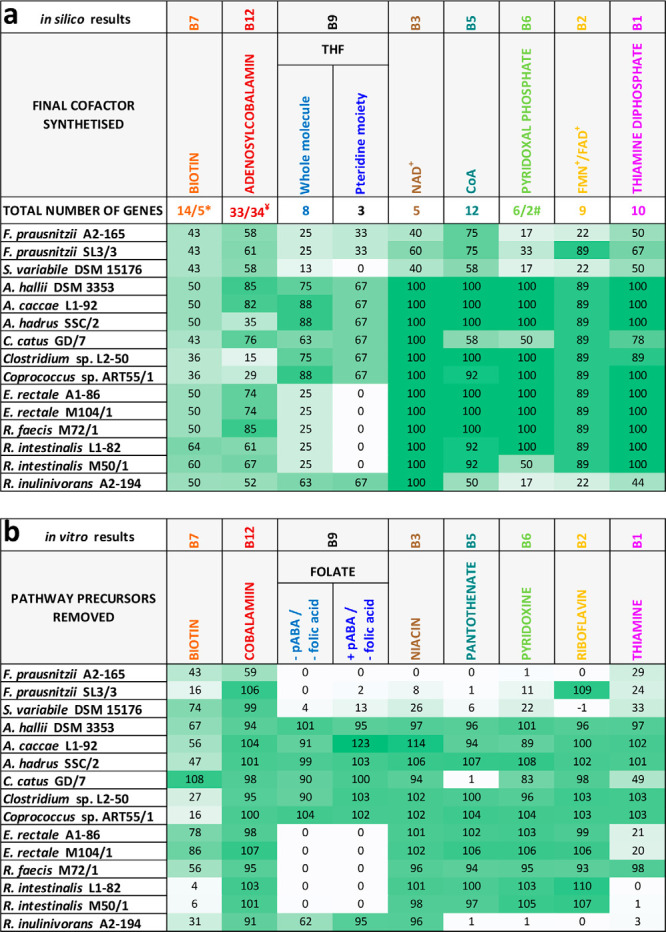
Overview of the *in silico* and *in vitro* results generated for eight vitamin pathways in 15 strains of butyrate-producing bacteria. (a) *In silico* results. The percentage of gene presence in the *de novo* biosynthetic pathways is shown (when different routes of vitamin biosynthesis are present, only the highest percentage of genes is displayed; for details, see Fig. S1 and Table S1). *, 14 genes in the main route (all strains except *R. intestinalis* M50/1) and five genes in the alternative route (*R. intestinalis* M50/1); ¥, either one or two genes for step 13; #, six genes in the main route (*F. prausnitzii*, *S. variabile* DSM 15176, *R. inulinivorans* A2-194) and two genes in the alternative route (rest of the strains). (b) *In vitro* results. The percentage of growth in the absence of the respective vitamin or precursor relative to the positive control with all vitamins present is shown (significant differences are given in Fig. 3). pABA, *p*-aminobenzoic acid. Color gradients reflect percentage (0% [white] to the maximum percentage [dark green]).

The complete analysis for all vitamins investigated is provided in Fig. S1 and Table S1 in the supplemental material, and an overview of the results is given in Fig. 2a. Most of the examined strains harbored all or most of the genes for the biosynthesis of the cofactors related to niacin, pantothenate, pyridoxine, riboflavin, and thiamine. Biosynthetic pathways of biotin, cobalamin, and folate, on the other hand, were partially or completely absent for many of the strains investigated. *F. prausnitzii* strains and Subdoligranulum variabile, belonging to the family *Ruminococcaceae*, lacked more genes than most of the bacteria belonging to the family *Lachnospiraceae*. Within the *Lachnospiraceae*, most of the *Roseburia* spp. and E. rectale strains did not harbor pathways for folate biosynthesis.

10.1128/mBio.00886-20.1 FIG S1 KEGG maps showing the pathways for the synthesis of eight B vitamins. The main, alternative, and salvage routes or the different sections of complex routes are highlighted in different colors. Download FIG S1, PDF file, 0.5 MB. Copyright © 2020 Soto-Martin et al. 2020 Soto-Martin et al. This content is distributed under the terms of the Creative Commons Attribution 4.0 International license.

10.1128/mBio.00886-20.5 TABLE S1 List of genes for the synthesis of eight B vitamins found in the genomes of the 15 strains of the study. The NCBI Protein IDs were retrieved from the PATRIC and KEGG databases and also obtained via BLASTP. Download Table S1, XLSX file, 0.1 MB. Copyright © 2020 Soto-Martin et al. 2020 Soto-Martin et al. This content is distributed under the terms of the Creative Commons Attribution 4.0 International license.

A visual inspection of 20 amino acid pathways (Fig. S2) in the PATRIC database revealed that most were present in the genomes of the strains of this study, and some missing genes were identified by manual BLASTP analysis (Table S2). In a few strains, single genes could not be identified for the following amino acids: asparagine, threonine, lysine, histidine, tyrosine, and the central pathway branch of aromatic amino acids to chorismate. For *Coprococcus* sp. strain ART55/1, three genes for chorismate could not be identified and *F. prausnitzii* strains, *Subdoligranulum variabile* DSM 15176, *Roseburia inulinivorans* A2-194, and *Coprococcus catus* GD/7 appeared to lack several genes for tryptophan biosynthesis (Table S2). For alanine and methionine synthesis, several aminotransferases were not present in certain strains, but other aminotransferases with homologous domains were found by manual BLASTP (Table S2).

10.1128/mBio.00886-20.2 FIG S2 KEGG maps showing the pathways for the synthesis of the 20 proteinogenic amino acids. The enzymes of predefined KEGG modules are marked in pink. Alternative colors have been used to highlight the enzymes of modules defined by the authors based on the scientific literature. Download FIG S2, PDF file, 1.1 MB. Copyright © 2020 Soto-Martin et al. 2020 Soto-Martin et al. This content is distributed under the terms of the Creative Commons Attribution 4.0 International license.

10.1128/mBio.00886-20.6 TABLE S2 Overview of the *in silico* results generated for 20 amino acid pathways in 15 strains of butyrate-producing bacteria. Results are expressed as the number of genes absent in an amino acid pathway. When different routes of amino acid biosynthesis are possible (see Fig. S2), only the route with the highest number of genes present is displayed. Results from manual blastp analyses for genes not found in the PATRIC and KEGG databases are provided in separate tabs. Download Table S2, XLSX file, 0.1 MB. Copyright © 2020 Soto-Martin et al. 2020 Soto-Martin et al. This content is distributed under the terms of the Creative Commons Attribution 4.0 International license.

### Validation of auxotrophic and prototrophic strains *in vitro*.

**FIG 3:**
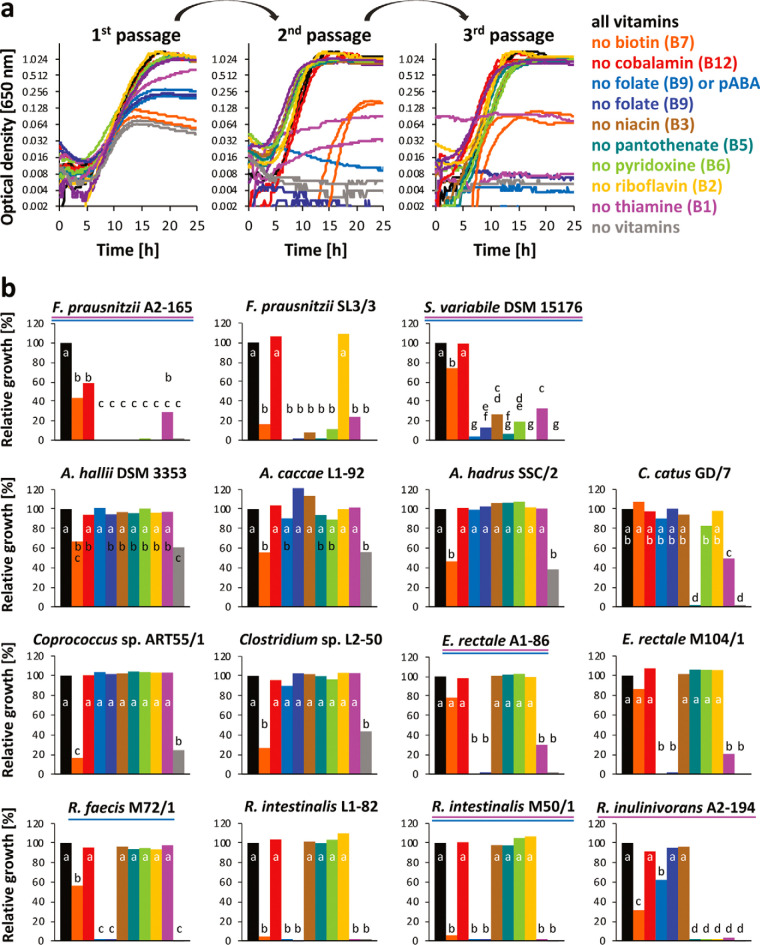
Growth of butyrate-producing bacteria in the absence of vitamins. (a) Growth of *R. intestinalis* M50/1 in 96-well plates during three passages in CAH-CDM lacking individual vitamins and their respective precursors in comparison to a positive control (black lines) with all vitamins and a negative control (gray lines) with none of the vitamins present. (b) Final optical densities relative to the positive control of the third passage of 15 different strains. *F. prausnitzii* strains and C. catus GD/7 were grown in Hungate tubes. All other strains were grown in 96-well plates. The final OD values reached by the control with all vitamins ranged from 0.392 to 1.372 for the different strains. All growth curves are given in Fig. S3 in the supplemental material. The pooled standard deviation ranged between 2.2 and 14.7 per strain. Letters refer to the Tukey test results. Treatments with a letter in common are not significantly different (*P* value of <0.05). Strains used as thiamine and folate auxotrophs in synthetic community cross-feeding experiments are underlined in pink and blue, respectively.

In order to assess auxotrophies *in vitro*, completely defined vitamin-free or amino acid-free media were developed. Vitamin-free casein acid hydrolysate or individual amino acids were used to replace Bacto Casitone and yeast extract for vitamin and amino acid auxotrophy tests, respectively, in addition to other micronutrients and trace elements. Most *Lachnospiraceae* species grew well in the defined media, but the two *Ruminococcaceae* species exhibited poorer growth, and of four different strains of *F. prausnitzii* initially tested (A2-165, SL3/3, M21/1, and L2-6), only two (A2-165 and SL3/3) could be grown in fully defined media (data not shown) and were included for further analyses. All strains were grown in the absence of one of the eight vitamin-cofactors and their precursors to validate the *in silico* findings. Grown cultures were subcultured twice to ensure that carryover of residual vitamins from the preculture containing all vitamins did not affect the results. Representative growth curves are shown for Roseburia intestinalis M50/1 in Fig. 3a (all growth curves are shown in Fig. S3), and the final growth achieved during the third passage in the absence of individual vitamins relative to the positive control for all strains is shown in Fig. 3b. Most bacterial strains could grow in the absence of cobalamin, niacin, pantothenate, pyridoxine, and riboflavin, whereas several bacteria showed suboptimal or no growth in the absence of biotin, folate, and thiamine. Bacterial strains with the highest number of auxotrophies were *F. prausnitzii* A2-165 and SL3/3, S. variabile DSM 15176, and R. inulinivorans A2-194. Most strains belonging to the *Roseburia/*E. rectale group showed no growth in the absence of only folic acid or both tetrahydrofolate (THF) precursors (*p*-aminobenzoic acid and folic acid). Figure 2b presents an overview of the *in vitro* results in comparison to *in silico* data (Fig. 2a), which shows that growth experiments were in good agreement with the genomic predictions for folate, niacin, pantothenate, pyridoxine, and riboflavin. However, *in vitro* and *in silico* data showed some disagreements for biotin, cobalamin, and thiamine.

**FIG 4:**
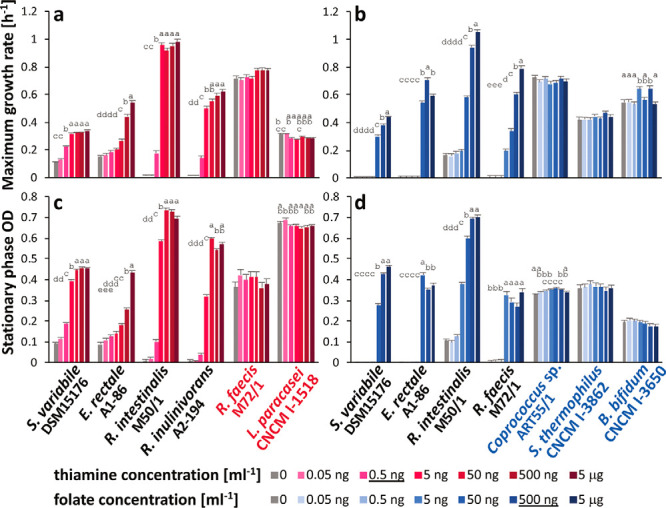
Pure culture growth parameters of strains grown in the presence of increasing concentrations of thiamine (red) or folate (blue). (a to d) Graphs show maximum growth rate achieved in exponential phase (a and b) and show the optical density reached in stationary phase (c and d). Auxotrophic strains are in black font, and thiamine and folate prototrophic strains are in red and blue font, respectively. The corresponding growth curves are shown in Fig. S3d. The concentrations closest to the estimated *in vivo* concentrations (34) are underlined in the legend. Letters refer to analysis of variance test; treatments with different letters are significantly different (*P* value of <0.05; no letters implies no significant difference).

10.1128/mBio.00886-20.3 FIG S3 Growth data of vitamin experiments in pure and mixed culture. (a) Number of replicates for vitamin auxotrophy screen to establish growth relative to the positive control in the presence of all vitamins. Vitamin concentrations are given in Table S5 apart from strains A2-165, SL3/3, DSM 15176, L1-92, A1-86, M104/1, and A2-194, which were grown at 50× higher levels. (b) Growth curves for vitamin auxotrophy screen at a single vitamin concentration. Gray lines indicate data used for Fig. 3 (thin lines show growth in Hungate tubes and a single OD reading, and thick lines show growth in 96-well plate and average data over 1 h). (c) Number of replicates for pure culture growth (stationary OD/growth rate) in the presence of increasing concentrations of folate or thiamine. (d) Growth curves for all vitamin conditions and strains tested. Gray lines indicate data used for Fig. 4c and d. Download FIG S3, PDF file, 1.2 MB. Copyright © 2020 Soto-Martin et al. 2020 Soto-Martin et al. This content is distributed under the terms of the Creative Commons Attribution 4.0 International license.

As the *in silico* investigation of amino acid biosynthetic pathways revealed the presence of complete pathways for most strains and amino acids examined, *in vitro* experiments were conducted only with selected strains. All strains displayed levels of growth similar to that of the positive control in AA-CDM (chemically defined medium with a mix of free amino acids [see Materials and Methods]) lacking alanine, asparagine, threonine, lysine, or histidine (Fig. S4), indicating that the absence of single genes is likely due to poor genome annotation and that genes that could be found by manual BLASTP analysis were correctly identified. The absence of methionine resulted in a reduction both in the level of growth and the maximum growth rate (μ, h^−1^) in Anaerostipes caccae L1-92 over both the positive control with all amino acids and the negative control with no amino acids (apart from cysteine) present (Fig. S4; μ = 0.68 ± 0.02 for all amino acids, 0.37 ± 0.02 for no amino acids, 0.10 ± 0.01 for absence of methionine only, *P* < 0.05). *F. prausnitzii* SL3/3, *S. variabile* DSM 15176, and R. inulinivorans A2-194, which had genes missing for tryptophan biosynthesis from chorismate, showed no growth in the absence of tryptophan (Fig. S4). *Coprococcus* sp. ART55/1 and *S. variabile* DSM 15176 had missing genes in the central pathway for aromatic amino acid biosynthesis to chorismate, but *Coprococcus* sp. ART55/1 was able to grow in the absence of all aromatic amino acids, whereas *S. variabile* DSM 15176 failed to grow (Fig. S4). Growth patterns in the absence of different combinations of all three aromatic amino acids agreed with *Coprococcus* sp. ART55/1 being prototrophic for all and *S. variabile* DSM 15176 being auxotrophic only for tryptophan (data not shown), indicating that the genes lacking in the chorismate pathway for these two strains are due to incomplete genome information. Overall, the results of the selected growth tests suggest that the bacteria investigated here are largely prototrophic for all amino acids apart from tryptophan.

10.1128/mBio.00886-20.4 FIG S4 Relative growth of several butyrate-producing strains in the third passage of culture in AA-CDM lacking certain amino acids compared to the positive control with all amino acids present. (a) Overview of strains and amino acids tested in comparison to the positive control in the presence of all amino acids. The growth of strains tested in the absence of specific amino acids is highlighted. Numbers in gray refer to *in silico* data as per Table S2. (b) Growth curves (left) and stationary OD relative to positive control (right) for all amino acid conditions and strains tested. Gray lines indicate data used for stationary OD data. The pooled standard deviations ranged between 3.9 and 30.6 per strain. Letters refer to the Tukey test results. Treatments with different letters in common are not significantly different (*P* value < 0.05). Download FIG S4, PDF file, 0.6 MB. Copyright © 2020 Soto-Martin et al. 2020 Soto-Martin et al. This content is distributed under the terms of the Creative Commons Attribution 4.0 International license.

### Cross-feeding between vitamin auxotrophs and prototrophs in synthetic communities.

The question arises whether the auxotrophies identified here are likely to influence bacterial growth characteristics *in vivo*. We therefore examined the growth behavior of selected auxotrophic strains for the two vitamins that several auxotrophies were identified for, thiamine and folate. Colonic quantities of those vitamins were estimated by O’Keefe et al. (34), who found that folate levels were high (approximately 0.46 μg/g colonic effluent; for comparison, 1× CDM contains 0.05 μg/ml, 0.11 μM). Colonic thiamine concentrations on the other hand were low (approximately 0.001 μg/g; 1× CDM = 0.05 μg/ml, 0.15 μM). Five auxotrophic strains per vitamin were grown in the presence of different thiamine and folate concentrations spanning the estimated *in vivo* concentrations (0.05 ng/ml to 5 μg/ml). We also examined whether prototrophic strains were able to stimulate growth of auxotrophic strains, which would demonstrate their ability to cross-feed vitamins (in the absence of external vitamins) or other growth factors (in the presence of external vitamins). The prototrophic strains tested comprised some of the butyrate producers investigated here (Roseburia faecis M72/1 for thiamine, *Coprococcus* sp. ART55/1 for folate) as well as non-butyrate-producing bacteria (Lactobacillus paracasei CNCM I-1518 for thiamine, Streptococcus thermophilus CNCM I-3862 and Bifidobacterium bifidum CNCM I-3650 for folate). After growth in vitamin-containing media, all strains were washed to remove medium vitamins before growth experiments in pure or mixed culture were carried out. *F. prausnitzii* A2-165 did not show any growth in pure culture in CDM after this procedure, but a parallel culture in M2GSC medium (35) grew, revealing that the cells were still viable after the washing procedure (data not shown). All other auxotrophic strains grew in CDM (Fig. 4). Auxotrophic strains displayed growth that depended on vitamin concentration, while prototrophic strains displayed similar growth characteristics independent of the vitamin concentration (Fig. 4 and Fig. S3).

**FIG 5:**
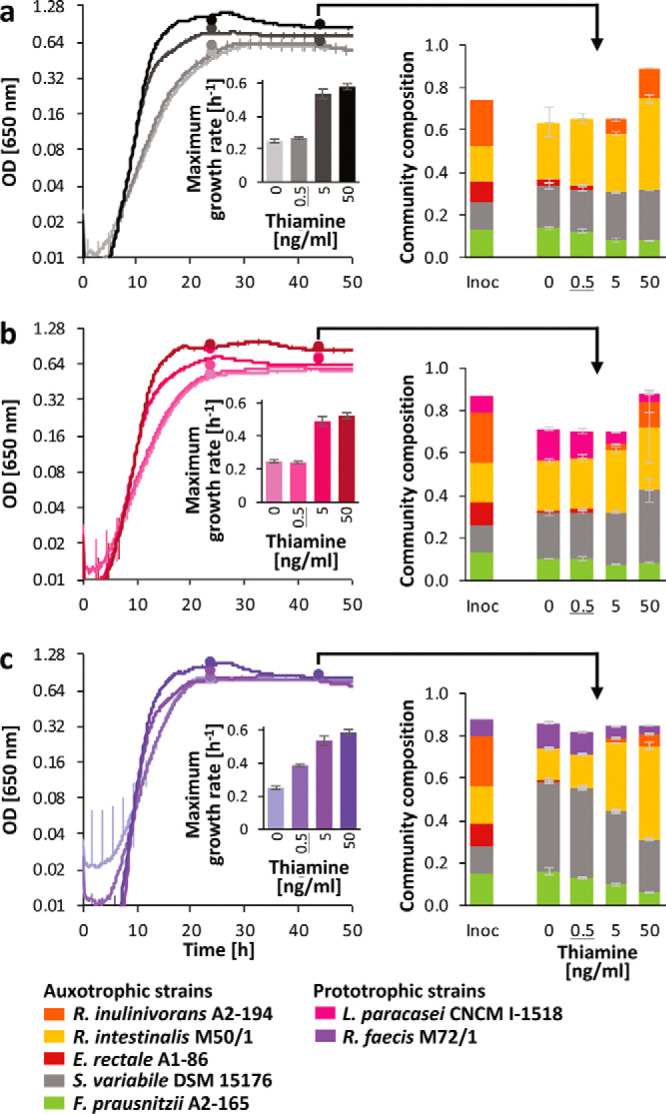
Coculture growth in 96-well plates in the presence of increasing thiamine concentrations. (a to c) Growth curves and their corresponding maximum growth rates are shown for a five-membered auxotrophic community (a) or the same community with a thiamine prototrophic strain (*L. paracasei* CNCM I-1518 [b] or *R. faecis* M72/1 [c]). Error bars are means of 12 individual time points to facilitate visibility of the curves. Colored stacked bar graphs show the relative community composition of the inoculum (Inoc) (scaled to 100× relative to grown cultures for visibility) and after 44 h of community growth in a replicate plate set up for sampling as indicated by the arrows (individual data points on the growth curves show optical density of the replicate plate after 24 and 44 h of incubation). The sum of the percentage of all strains is scaled to the OD of the original sample. The thiamine concentration closest to the estimated *in vivo* concentrations (34) is underlined.

**(i) Thiamine.** The thiamine auxotrophs *S. variabile* DSM 15176, Eubacterium rectale A1-86, R. intestinalis M50/1, and R. inulinivorans A2-194 showed optimum growth only with 5 ng/ml thiamine or higher levels, which is above the estimated *in vivo* concentration (34). E. rectale A1-86 was most severely affected by lower thiamine concentrations and exhibited reduced growth at all concentrations below 5 μg/ml (Fig. 4a and c). Thiamine cocultures with the auxotrophic strains including *F. prausnitzii* A2-165 plus either L. paracasei CNCM I-1518 or R. faecis M72/1 as prototrophs were grown in the absence of thiamine and over the range of 0.5 to 50 ng/ml thiamine (Fig. 5). Analysis of variance revealed that community composition, thiamine concentration, and their interaction had a significant effect (*P* < 0.05) on growth rate, overall stationary optical density (OD), and the abundance of all strains apart from *S. variabile* DSM 15176, for which the thiamine concentration did not reach significance. Growth was observed under all conditions in the five-membered coculture containing only auxotrophic strains, but stationary-phase OD was significantly lower at 0, 0.5, and 5 ng/ml thiamine compared to 50 ng/ml thiamine (*P* < 0.0001), and the maximum growth rate was significantly reduced (*P* < 0.0001) at the two lower thiamine concentrations compared to the two higher ones (Fig. 5a). The microbial community composition was dominated by *R. intestinalis* M50/1 and *S. variabile* DSM 15176. *F. prausnitzii* A2-165 was also present with higher abundance at the two lower vitamin concentrations (*P* < 0.0001). Thus, despite not being able to grow in pure culture under the experimental conditions, it appears that the presence of the other bacteria enabled growth of this strain. *R. inulinivorans* A2-194 was present only at higher thiamine concentrations, whereas E. rectale A1-86 showed little growth and was only detected at the lower thiamine concentrations (Fig. 5a). In the presence of the prototrophic strain L. paracasei CNCM I-1518 (Fig. 5b), overall growth and community composition were similar to those of the respective auxotrophic cocultures, with significantly higher overall growth at the highest thiamine concentration (*P* < 0.0001), and the prototrophic strain achieved better relative growth with 0 and 0.5 ng/ml thiamine than with the higher thiamine concentrations (*P* < 0.0001; the percentage of *L. paracasei* CNCM I-1518 of the total community ranged from 21.4 ± 0.8 to 4.2 ± 0.7 across all thiamine concentrations). This suggests that this strain had little impact on the community and was not an efficient cross feeder of thiamine. In the presence of prototroph *R. faecis* M72/1 (Fig. 5c), on the other hand, stationary ODs were similar for all treatments, and the growth rate in the presence of 0.5 ng/ml thiamine was significantly higher than in the other two cocultures (auxotrophs only and auxotrophs plus *L. paracasei* CNCM I-1518, *P* < 0.0001). *S. variabile* DSM 15176 was most strongly affected by the presence of *R. faecis* M72/1 and achieved better growth in the absence of thiamine and at the two lower concentrations compared to the other two cocultures (*P* < 0.0001). Similar to prototrophic strain *L. paracasei* CNCM I-1518, *R. faecis* M72/1 competed better at the two lower thiamine levels (*P* < 0.0001, 14.2% ± 0.6% to 4.5% ± 0.2% of the total community across all thiamine concentrations).

**FIG 6:**
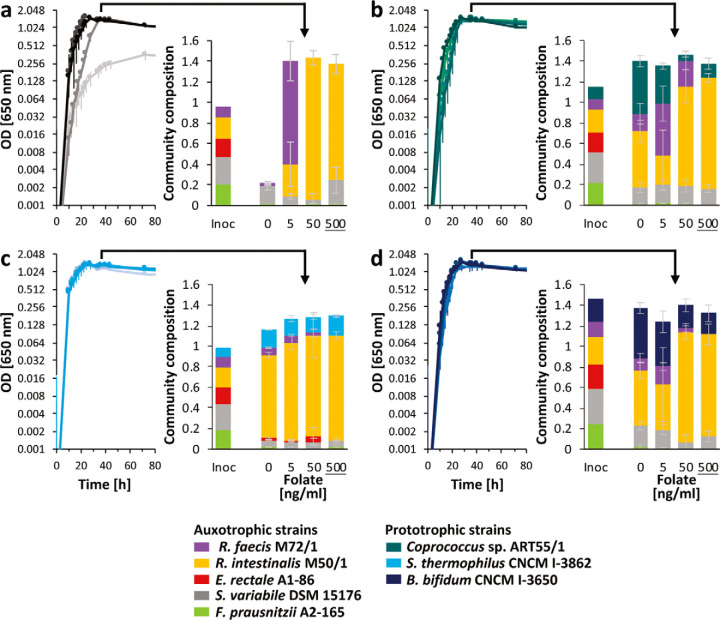
Coculture growth in Hungate tubes in the presence of increasing folate concentrations. (a to d) Growth curves (increasing darkness with increasing folate concentrations) are shown for a five-membered auxotrophic community (a) or the same community with a folate prototrophic strain (*Coprococcus* sp. ART55/1 [b], S. thermophilus CNCM I-3862 [c], or *B. bifidum* CNCM I-3650 [d]). Colored stacked bar graphs show the relative community composition of the inoculum (Inoc) (scaled to 300× relative to grown cultures for visibility) and after 36 h of growth in stationary phase as indicated by the arrows. The sum of the percentage of all strains is scaled to the OD of the original sample. The folate concentration closest to the estimated *in vivo* concentrations (34) is underlined.

**(ii) Folate.** The folate auxotrophic strains S. variabile DSM 15176, E. rectale A1-86, R. intestinalis M50/1, and R. faecis M72/1 mostly showed reasonable growth in the presence of at least 50 ng/ml folate (approximately 10-fold lower than estimated under *in vivo* conditions [34]) in pure culture (Fig. 4d), although the growth rates were significantly lower at all but the highest folate concentration of 5 μg/ml for most strains (Fig. 4b). Concentrations below 5 to 50 ng/ml resulted in a complete absence of growth for most auxotrophs. Extended lag phases were observed for *S. variabile* DSM 15176, E. rectale A1-86, and *R. faecis* M72/1, even at close to estimated *in vivo* folate concentrations of 500 ng/ml for some strains (Fig. S3d). Folate cocultures were carried out with the above strains and *F. prausnitzii* A2-165 in Hungate tubes to provide more optimal growth conditions for *F. prausnitzii* A2-165, as it tended to show poorer growth in the anaerobic cabinet in 96-well plates than in Hungate tubes (data not shown). A higher concentration range (up to 500 ng/ml) was chosen than for thiamine to reflect the estimated *in vivo* availability of folate. Analysis of variance revealed a significant effect (*P* < 0.05) of community composition, folate concentration, and their interaction on stationary OD and abundance of all strains apart from E. rectale, which was significantly affected only by community composition. The five-membered auxotrophic microbial community showed very limited growth in the absence of folate, with *S. variabile* DSM 15176 being the main bacterium detected. At 5 ng/ml folate, *R. faecis* M72/1 became the dominant strain, followed by *R. intestinalis* M50/1, which became the dominant strain at the two highest folate levels (Fig. 6a). Three different prototrophic bacteria were tested for their cross-feeding behaviors to the same auxotrophic community, *Coprococcus* sp. ART55/1 (Fig. 6b), S. thermophilus CNCM I-3862 1 (Fig. 6c), and B. bifidum CNCM I-3650 1 (Fig. 6d). Overall community growth was similar in the absence and presence of folate with all prototrophic strains, with *Coprococcus* sp. ART55/1 and *B. bifidum* CNCM I-3650 constituting 36.4% ± 3.8% and 35.3% ± 3.4% of the community, respectively, in the absence of folate. S. thermophilus CNCM I-3862, on the other hand, mainly boosted the growth of the auxotrophic strains, in particular *R. intestinalis* M50/1, and contributed only 14.9% ± 0.4% to the whole community (Fig. 6). Furthermore, in the presence of S. thermophilus CNCM I-3862, the community profiles in the absence of folate were similar to those of the high-folate cultures, and E. rectale A1-86 became detectable (up to 2.3% at the two lower folate concentrations) (Fig. 6c).

## DISCUSSION

Vitamin and amino acid concentrations in the large intestine are the result of a balance between the influx from the small intestine, the utilization and production by the gut microbiota, and host absorption (36, 37). Differences in the concentrations of B vitamins have been observed in colonic contents in healthy individuals (34), and the levels may be affected by dietary habits or certain disease states, which could have an impact on the gut microbial community (38). Here, we combined *in silico* analysis of bacterial genome sequences with *in vitro* growth tests to establish the biosynthetic capacity for vitamins and amino acids in 15 butyrate-producing bacteria of the human large intestine. An initial comparison of genomic data based on the web platform PATRIC and growth data showed certain divergences, but further manual searches revealed that some missing genes were in fact present. This highlights both the relevance of *in vitro* testing and the limitations of the genomic information deposited in public databases. A recent study of vitamin biosynthetic pathways in genomes of more than 2,000 bacteria included all but one (*R. faecis* M72/1) of the strains investigated here (18). Our gene presence percentages are based on complete pathways, whereas Rodionov et al. (18) defined a subsection of signature genes as indicators for *de novo* biosynthesis by ignoring genes not detected in all experimentally verified prototrophs. When taking only those signature genes into consideration, our data are in agreement with their predictions for biotin, folate, niacin, pantothenate, pyridoxine, riboflavin, and thiamine, but for most strains designated as prototrophic for cobalamin by Rodionov et al. (18), not all signature genes for cobalamin biosynthesis could be identified here (see Table S1 in the supplemental material).

Due to the limitations in genome annotations (7), it is important to confirm *in silico* results with *in vitro* growth tests. Human gut bacteria, especially some species belonging to the *Firmicutes*, can be difficult to grow, and complex medium ingredients are routinely used. Completely defined media had to be established to test micronutrient requirements, and to our knowledge, the majority of the strains examined here have not been grown in such media before. For most of the *Lachnospiraceae* species, good growth was achieved in the two media used here, but the *Ruminococcaceae* proved more fastidious, and two strains of *F. prausnitzii* (M21/1 and L2-6) failed to grow even after including medium ingredients reported by others to enable growth of this species (21). With regard to amino acid biosynthesis, our results indicate that the investigated strains are mostly prototrophic, which is in agreement with previous studies (39). An inability to grow was found only in the absence of tryptophan for three strains, in accordance with these strains lacking several genes in the tryptophan biosynthetic pathway. Other gene absences (mostly single genes) were not confirmed *in vitro* and are likely due to inaccuracies in the genome annotations or missing sequence data. The level of completeness of the different genomes analyzed here varied considerably, ranging from complete genomes to 179 nonscaffolded contigs (Table S3). Thus, the absence of three genes for chorismate biosynthesis in *Coprococcus* sp. ART55/1 (total assembly gap length, 235 kb), which would not be confirmed by growth tests, may be due to missing sequence information. Furthermore, aminotransferases for alanine and methionine biosynthesis tend to have overlapping substrate specificities (40) and may not be correctly assigned.

10.1128/mBio.00886-20.7 TABLE S3 Bacterial strains of the study and genome information. Download Table S3, PDF file, 0.2 MB. Copyright © 2020 Soto-Martin et al. 2020 Soto-Martin et al. This content is distributed under the terms of the Creative Commons Attribution 4.0 International license.

Recently, Tramontano et al. (7) evaluated the inhibitory effect of amino acids on the growth of certain bacterial strains. Bacteroides clarus, Bacteroides xylanisolvens, and Parabacteroides merdae were sensitive to the presence of aromatic amino acids. Here, we mostly did not observe major differences in growth rate in different media. However, a large reduction in growth rate was found for *A. caccae* L1-92 when only methionine was removed from the medium compared to the other conditions tested, including the negative control lacking all amino acids except cysteine. This indicates that the absence of methionine in the presence of other amino acids may influence the regulation of amino acid biosynthetic pathways.

The species with the highest number of vitamin auxotrophies were *F. prausnitzii*, *S. variabile*, and the E. rectale/*Roseburia* group. Previous studies have found that increased consumption of B vitamins (in particular riboflavin, cobalamin, folate, and pyridoxine) is associated with a higher abundance of these gut bacteria (41, 42). Intriguingly, these species are among the most prevalent and abundant species in the human gut microbiota (Fig. 1), but the auxotrophies observed for B vitamins may make them more sensitive to environmental variation. The growth tests were in good agreement with the *in silico* predictions for most of the vitamins, but discrepancies were found for thiamine, biotin, and cobalamin. While both E. rectale and *R. intestinalis* strains investigated had all the genes needed for the synthesis of thiamine, they were unable to grow in the absence of thiamine. It is not clear whether synthesis genes are not expressed or not functional in these bacteria, as they may be adapted to salvage the precursor from the environment, or whether molecules present in the medium could be repressing certain genes. Three of the four signature genes for biotin biosynthesis defined by Rodionov et al. (18) (BioF, BioA, and BioD) were also found to be absent in most of our strains; however, growth was not completely curtailed in the majority of strains in the absence of biotin even after three passages in biotin-free media. Biotin is a cofactor of different carboxylase enzymes involved in fatty acid biosynthesis, amino acid metabolism, and the tricarboxylic acid cycle (43, 44). Some studies carried out in the 1940s and 1950s indicated that biotin needs are determined by certain anabolic processes such as aspartate production in certain species of lactic acid bacteria, which had lower biotin needs when aspartate was supplied (45, 46). Therefore, the presence of amino acids could lower biotin requirements. There is little information about the levels of vitamins required by gut bacteria, but certain studies have estimated the intracellular concentrations of this cofactor in other bacteria to be quite high (40 μM biotin in the cytoplasm of Escherichia coli [47]) compared to other vitamins (15).

None of the strains investigated here had all the genes required for vitamin B_12_ coenzyme synthesis. Rodionov et al. (18) and Shelton et al. (48) reported that cobalamin biosynthetic pathways are often incomplete even in strains with verified prototrophic status. When assessing the signature genes reported in their studies, only *R. faecis* M72/1 contained all 18 genes identified by Rodionov et al. (18), and the seven genes defined by Shelton et al. (48) were found here in only six of our strains (Table S1). For most other strains, more than 70% of the signature genes from either study were present, but Anaerostipes hadrus SSC/2, *Clostridium* sp. strain L2-50, and *Coprococcus* sp. ART55/1 contained hardly any cobalamin biosynthetic genes and are likely bona fide auxotrophs. Our *in vitro* tests however showed good growth in the absence of cobalamin for all strains except *F. prausnitzii* A2-165. Some gut microbes may not require cobalamin, as they have B_12_-independent enzymes (11). BLASTP analyses of the 15 genomes of this project indicate the presence of the enzyme MetE (a B_12_-independent alternative enzyme to the B_12_-dependent MetH involved in methionine metabolism) in some of the strains, including the three strains not harboring the cobalamin biosynthetic pathway (Table S4). Vitamin B_12_ is also required for tRNA synthesis, but a B_12_-independent enzyme, epoxyqueuosine reductase (QueH) has been reported (48, 49). QueH was identified in all of our strains apart from *R. intestinalis* M50/1 and *Coprococcus* sp. ART55/1 (Table S4), which may be due to missing data in the draft genomes (Table S3). Nucleotide biosynthesis also contains B_12_-dependent steps (48). However, the B_12_-independent enzyme variant NrdD (anaerobic ribonucleoside-triphosphate reductase class III) appears to be present in the strains examined here (Table S4) and nucleotides were provided in our medium, therefore, external cobalamin would not be required in our experimental setup to satisfy nucleotide requirements. Overall, our data suggest that some of the strains studied here are genuine cobalamin auxotrophs, but the presence of methionine and nucleotides in the medium and the carriage of B_12_-independent genes enabled their growth in the absence of B_12_. However, we cannot exclude the possibility that the medium contained traces of cobalamin or cobalamin precursors.

10.1128/mBio.00886-20.8 TABLE S4 Strains that encode the B_12_-independent enzymes MetE, QueH, and NrdD in their genome. Details of the BLASTP analyses are provided: query sequence, matches, percentages of query coverage, and percentage identity. Download Table S4, XLSX file, 0.03 MB. Copyright © 2020 Soto-Martin et al. 2020 Soto-Martin et al. This content is distributed under the terms of the Creative Commons Attribution 4.0 International license.

Next, we wanted to explore whether the identified auxotrophic strains would benefit from the presence of prototrophic strains. For this, we established synthetic gut microbial communities and monitored their response to different prototrophs at different concentrations of vitamins. Synthetic communities offer the opportunity to study bacterial interactions in a controlled system (50) and have been used to dissect microbial interactions to guide the development of mathematical models (51, 52). Here, we used five-membered synthetic communities to dissect the response of thiamine and folate auxotrophic bacteria to vitamins and prototrophic strains. Pure culture growth of the thiamine auxotrophs showed growth impairment even under thiamine concentrations in excess of the estimated *in vivo* concentrations, which indicates that thiamine could be a growth-limiting factor *in vivo*. Better growth was seen in coculture in the absence of thiamine, which may be due to cross-feeding of other nutrients that can partially alleviate the auxotrophy or cell lysis and liberation of thiamine by some strains. There were large differences in the relative abundance of different strains after coculture growth in the presence of vitamins, with *R. intestinalis* M50/1 dominating most communities, followed by *S. variabile* DSM 15176. This suggests differences in the ability of different strains to compete for nutrients or intrinsic differences in growth characteristics (such as maximum growth rates or gene regulatory differences). In the absence of vitamins, the relative abundance of auxotrophic strains differed, in particular for folate. Prototrophic strains had little impact on community composition and constituted only a small proportion of the whole community under vitamin-replete conditions but mostly became more abundant in the absence of vitamins, which shows their enhanced ability to compete under those conditions. However, they showed variable and strain-specific impact on auxotrophic strains in the absence of vitamins and differed in their ability to restore community composition to that seen under vitamin-replete conditions, which indicates differences in their capacity to share vitamins with other community members. Interestingly, the presence of *R. faecis* M72/1 under thiamine deficiency lead to an increase of *S. variabile* DSM 15176 at the expense of *R. intestinalis* M50/1, which may be due to stronger competition between the two *Roseburia* strains. The folate cocultures, which contained both *Roseburia* strains as auxotrophs, also indicate strong competition between them at low folate concentrations, with *R. intestinalis* M50/1 showing superior growth at high folate conditions. Stable coexistence of closely related species has mainly been investigated in *Bacteroidetes* to date and focused on carbohydrate metabolism (53), but our data indicate that vitamin requirements and potential differences in vitamin availability in gut microenvironments (54) should also be taken into consideration. However, other types of antagonistic interactions, such as bacteriocin production, may also contribute to competition between related strains. *F. prausnitzii* A2-165 showed no growth in synthetic media after a single wash to remove excess vitamins, but it was stimulated by the auxotrophic community, suggesting that it requires other growth factors provided by community members, and a strong dependency on other microbes in line with other studies (51, 52).

In conclusion, this is the first study in which the vitamin and amino acid requirements of a range of butyrate-producing bacterial species of the human gut have been assessed by a direct comparison of *in silico* and *in vitro* approaches. Interestingly, the *Ruminococcaceae* species (*F. prausnitzii* and *S. variabile*) exhibited a larger number of auxotrophies than those belonging to the *Lachnospiracea*e. Only a limited number of *Ruminococcaceae* were investigated here, and it remains to be confirmed whether this also applies to other species within this *Firmicutes* family. In our proof of concept coculture study, we provide evidence of cross-feeding between auxotrophic and prototrophic strains on vitamins B_1_ (thiamine) and B_9_ (folate). While the current experiments provide first insights into the capacity of prototrophic strains to stimulate the growth of poorly studied butyrate producers, further longitudinal experiments combining detailed tracking of each member of the community and vitamin are warranted. Auxotrophic and prototrophic bacteria could be affected differently in certain health conditions and under differing vitamin availabilities, both from the diet and via cross-feeding within the microbiota, which could be an important factor in determining microbiota composition and function as recently discussed by Steinert et al. (38). A better understanding of microbial vitamin requirements and microbe-microbe interactions will help in the design of microbiota-based approaches to support beneficial microbes.

## MATERIALS AND METHODS

### Bacterial strains.

Fifteen strains belonging to 12 dominant and subdominant butyrate-producing species from the *Lachnospiraceae* and *Ruminococcaceae* families with publicly available genomes were assessed for vitamin and amino acid auxotrophies (Fig. 1; see also Table S3 in the supplemental material). Stocks were routinely kept in M2GSC medium (35) and subcultured twice in chemically defined medium (CDM) (see below) before the start of the experiments (1% inoculum). Bifidobacterium bifidum CNCM I-3650, Lactobacillus paracasei CNCM I-1518, and Streptococcus thermophilus CNCM I-3862 (Table S3) from the Danone strain collection were used for coculture experiments. They were maintained in medium M17 plus 1 g/liter l-cysteine (S. thermophilus CNCM I-3862) or MRS plus 1 g/liter l-cysteine (*B. bifidum* CNCM I-3650, *L. paracasei* CNCM I-1518) (media were purchased from Oxoid, Thermo Fisher Scientific, Waltham, MA, USA) and subcultured in CDM before coculture experiments.

### Genomic analyses.

All genome analyses were carried out on the web platform PATRIC (https://www.patricbrc.org/, National Institutes of Allergy and Infectious Diseases, National Institutes of Health, MD, USA; accessed April-June 2017). It contains consistent annotations across all sequenced bacterial species from GenBank, curates whole genomes, and maps the annotated genes in KEGG maps (https://www.genome.jp/kegg/, Kanehisa Labs, Japan) (55). A visual inspection of each vitamin and amino acid pathway was carried out. The *de novo* biosynthetic pathways of the eight vitamin-related cofactors investigated were described before (15, 16). An anaerobic pathway branch for cobalamin recently identified (56) was searched for by manual BLASTP analysis (see details below). The pathways, the main and alternative routes with their different sections, are shown in Fig. S1. The synthesis of the 20 proteinogenic amino acids was evaluated by examining eight pathway families. All predefined KEGG pathway modules were utilized for most of the amino acids, but for some amino acids, modules had to be defined based on the literature (57). Amino acid pathway modules for all amino acids are shown in Fig. S2. Selected genes were searched for on the KEGG platform (between April 2017 to January 2019) or by using the BLASTP algorithm (58) on the EnsemblBacteria website (https://bacteria.ensembl.org/index.html, EBI and Welcome Trust Sanger Institute, Cambridgeshire, UK) with a query sequence from a phylogenetically closely related species (between October 2018 and January 2020). The cutoff values used to accept a match as true were set at 50% query coverage and at 40% identity (59). All results were confirmed on the UniProt website (https:///www.uniprot.org/, EMBL-EBI, UK; Swiss Institute of Bioinformatics, Switzerland; Protein Information Resource, USA). Matches with less than 50% query coverage and/or less than 40% identity were also considered true if they had the correct annotation.

### Design of chemically defined medium.

The composition of chemically defined medium (CDM) (Table S5) is based on the semidefined medium YCFA (60) but with modifications to the nitrogen sources: Bacto Casitone (an enzymatic digest of casein) and yeast extract were replaced with vitamin-free casein acid hydrolysate (CAH) or a mix of free amino acids (AA). When casein acid hydrolysate was used, the medium was supplemented with certain amino acids that are likely to be affected by the acid hydrolysis process (l-tryptophan, l-serine, l-threonine, l-glutamine, and l-asparagine) (61). Additionally, solutions of different nucleotides, trace elements, and vitamins were added to compensate for the lack of yeast extract in CDM. The vitamin concentrations in Table S5 are designated as 1×, but larger amounts of vitamins were used to grow certain strains (F. prausnitzii A2-165 and SL3/3, S. variabile DSM 15176, A. caccae L1-92, E. rectale A1-86, and M104/1, and R. inulinivorans A2-194; 50× vitamins over basal level). The pH ranged from 6.3 to 6.8 as detailed in Table S5.

10.1128/mBio.00886-20.9 TABLE S5 Final concentrations of ingredients in chemically defined media (CAH-CDM and AA-CDM). Download Table S5, PDF file, 0.2 MB. Copyright © 2020 Soto-Martin et al. 2020 Soto-Martin et al. This content is distributed under the terms of the Creative Commons Attribution 4.0 International license.

### *In vitro* assessment of vitamin and amino acid auxotrophies.

The synthesis of the cofactors of the vitamins biotin (B_7_), cobalamin (B_12_), folate (B_9_), niacin (B_3_), pantothenate (B_5_), pyridoxine (B_6_), riboflavin (B_2_), and thiamine (B_1_) and 9 amino acids (alanine, asparagine, threonine, methionine, lysine, histidine, and the aromatic amino acids tryptophan, phenylalanine, and tyrosine) was evaluated in 15 butyrate-producing strains (Table S3) in medium CDM. To determine vitamin auxotrophies, all precursors and the cofactor for a given vitamin were removed from the medium except for folate, for which two experimental treatments were set up to investigate whether the strains have the capacity to synthesize different moieties of the molecule of tetrahydrofolate (THF) (the pteridin ring and *para*-aminobenzoic acid). To assess amino acid auxotrophies, the removal of a single amino acid at a time from medium AA-CDM was carried out. For *Coprococcus* sp. ART55/1 and *S. variabile* DSM 15176, the removal of different combinations of aromatic amino acids was also investigated.

All amino acid experiments and most of the vitamin experiments were carried out in flat-bottom 96-well plates (Costar; Corning Inc., NY, USA) sealed with air-tight optical tape incubated at 37°C and read at 650 nm in a microplate reader (Epoch 2, BioTek Instruments Inc., VT, USA) inside a Don Whitley Anaerobic Workstation (Bingley, UK) (80% N_2_, 10% CO_2_, and 10% H_2_). The plates were shaken (double orbital mode, frequency: 425 cpm [3 mm]) and read every 10 min throughout the duration of growth experiments. Coprococcus catus GD7 displayed aggregative growth regardless of the type of medium or level of vitamins used, and both *F. prausnitzii* strains presented very slow growth. Thus, the vitamin experiments for these strains were carried out in Hungate tubes (gas phase 100% CO_2_) either to allow for a good mixing of the culture before determining optical density or to avoid long kinetic experiments in the plate reader. Hungate tubes were incubated statically at 37°C, homogenized, and read at 650 nm in a Novaspec II visible spectrophotometer (Pharmacia LKB Biotechnology AB, Uppsala, Sweden). The headspace in 96-well plates and Hungate tubes was one third of the total volume (100 μl and 5 ml, respectively).

For all auxotrophy tests (data shown in Fig. 2 and 3), a positive control (including all vitamin precursors/cofactors or amino acids) plus a negative control (lacking all vitamin precursors/cofactors or amino acids, except l-cysteine-HCl) were assessed in parallel. The preculture used as inoculum was not prewashed to avoid exposing the bacterial cells to stress and minimize risk of contamination or exposure to oxygen. All experimental treatments and controls were grown at least until early stationary phase in three consecutive passages (1% of inoculum) to limit nutrient carryover from the original inoculum. For all experimental treatments and controls, two technical replicates (designated plate or tube replicates) were run in parallel in the same experiment. Experiments were carried out independently at least twice, but when data from independent experiments were not conclusive, further replicates were performed (total number of replicates are given in Fig. S3). The optical density at 650 nm (OD_650_) achieved by the cultures in early stationary phase of the third passage of growth was used to assess growth relative to the positive control: relative growth under experimental conditions = (OD_650_ of experimental treatment/OD_650_ of positive control) × 100.

Growth curves were also visually examined for differences in growth rate between treatments, but no major differences were detected except where presented. Growth rates were determined from blank-subtracted exponential-phase data by selecting a time interval from semilogarithmic plots ensuring linearity from a trend line (most data had >10 data points and an *R*^2^ of >0.99, and all data with less than 5 data points and/or *R*^2^ of <0.97 were removed) with an OD_650_ of <0.4 (62).

### Coculture experiments of synthetic microbial communities.

Coculture experiments were carried out in CAH-CDM containing all vitamins except the one under study (thiamine or folate), which was added at a range of concentrations as detailed in Results (Fig. 4). The *in vitro* growth results from this study (Fig. 3b) were used to select auxotrophic and prototrophic strains. A background consortium of five auxotrophic bacteria per vitamin (F. prausnitzii A2-165, S. variabile DSM 15176, E. rectale A1-86, and R. intestinalis M50/1 for both vitamins, R. inulinivorans A2-194 for thiamine, and R. faecis M72/1 for folate) was grown on its own or alongside one of the following vitamin prototrophic strains: *R. faecis* M72/1 or *L. paracasei* CNCM I-1518 as potential thiamine cross-feeders; *Coprococcus* sp. ART55/1, S. thermophilus CNCM I-3862, or *B. bifidum* CNCM I-3650 as potential folate cross-feeders. Following a screening process, the L. paracasei strain was selected for its putative ability to produce thiamine based on its genomic sequence, and S. thermophilus and B. bifidum strains were chosen for their ability to produce folate in milk (data not shown). For pure culture and coculture experiments with various thiamine and folate concentrations, all strains were precultured once in CAH-CDM to exponential phase and residual medium vitamins were removed by centrifugation of 10-ml culture (658 × *g*, 10 min, room temperature) (Jouan MR 18 22; Sartorius, Gottingen, Germany) and resuspension in 10 ml CAH-CDM without carbon sources and vitamins. Viability was confirmed by observation of growth in M2GSC medium. For coculture experiments, equal amounts of individual strains based on optical density measurements were combined to create the synthetic community inoculum (inoculation at 1%). Growth experiments were carried out either in 96-well plates (all pure culture data and thiamine cocultures) or Hungate tubes (folate cocultures) as detailed above. For thiamine experiments, one 96-well plate was incubated in the plate reader to follow growth, and a replicate plate was incubated in an Eppendorf Thermomixer (Hamburg, Germany) with orbital shaking at 450 rpm every 10 min in a Don Whitley Anaerobic Workstation (Bingley, UK) for sample collection. Samples were taken for optical density measurements with a BioTek PowerWave x 340 microplate reader (BioTek Instruments Inc., VT, USA) and DNA extraction for community composition analysis as detailed below.

### Molecular community analysis.

DNA extraction of samples taken at the beginning of the stationary phase was carried out with the FastDNA Spin kit for soil (MP Biomedicals, Eschwege, UK), and DNA was quantified using a Qubit 3.0 fluorometer (Thermo Fisher Scientific, Paisley, UK). Microbial community analysis was carried out by multiplex PCR with primers targeting each community member and an internal standard added to the reaction mixture (genomic DNA from Corynebacterium glutamicum [Cg] DSM 1412 [DSMZ, Braunschweig, Germany]). Primers (Table S6) were designed in single-copy genes using NCBI Primer BLAST (https://www.ncbi.nlm.nih.gov/tools/primer-blast/) (63) and have a universal tag sequence at the 5′ end allowing for the incorporation of a fluorescent label into all amplicons for fragment analysis using the Beckman Coulter CEQ 8000 GeXP Genetic Analysis system (Beckman Coulter, High Wycombe, UK). All primers were purchased in water from Sigma-Aldrich (Gillingham, Dorset, UK) and purified by desalting with the exception of the TAG-for primer which was purified by high performance liquid chromatography (HPLC). The forward universal tag primer was fluorescently labeled with Cyanine 5. Multiplex PCRs (25-μl final volume, 10 ng sample DNA, 5 ng Cg, bacterium-specific primers [0.02 μM], universal tag primers [2 μM]) were set up with 1× Qiagen Multiplex PCR kit reagent (Qiagen, Manchester, UK) in PCR plates (Starlab, Milton Keyes, UK) (sealed with plate seal; Bio-Rad, Hemel Hemsptead, UK). PCR cycling conditions were as follows: 15 min at 95°C; 18 cycles, with 1 cycle consisting of 30 s at 94°C, 90 s at 57°C, and 60 s at 72°C; a final cycle of 10 min at 72°C and then 10°C hold. To generate normalization response factors (Rf) for each strain, PCRs were set up as previously but with equal amounts (5 ng) of genomic DNA from each individual pure strain and Cg in the same reaction.

10.1128/mBio.00886-20.10 TABLE S6 Primers used for molecular community analysis. Download Table S6, PDF file, 0.1 MB. Copyright © 2020 Soto-Martin et al. 2020 Soto-Martin et al. This content is distributed under the terms of the Creative Commons Attribution 4.0 International license.

PCR products were prepared for fragment analysis using the Beckman Coulter CEQ 8000 GeXP Genetic Analysis system (Beckman Coulter, High Wycombe, UK). An aliquot of PCR was diluted 1:10 in water, and then 2 μl was added to 30 μl sample loading solution (AB Sciex, Warrington, UK) and 0.375 μl CEQ DNA Size Standard 400 (AB Sciex, Warrington, UK), giving a final dilution of 1:160. The samples were mixed, placed in a 96-well CEQ electrophoresis plate (Axygen, Corning, Flintshire, UK) overlaid with mineral oil (AB Sciex, Warrington, UK), and analyzed by capillary electrophoresis and fragment separation of amplicons with the GeXP Genetic Analysis System using conditions as follows: capillary temperature of 50°C, denaturation for 120 s at 90°C, injection for 30 s at 2.0 kV, and separation for 40 min at 6.0 kV. Raw data were processed and filtered using the default criteria of the fragment analysis module of the GenomeLab GeXP system software. Data were quality checked to ensure that they were within the linear range and that unexpected or comigrating peaks were absent. Peak heights (relative fluorescence units [rfu]) were used for subsequent data analysis.

Response factors for each strain were calculated from PCRs containing equal amounts of genomic DNA of the strain and Cg by taking the peak height (rfu) ratio of strain/Cg (= Rf). Peaks for each strain detected in the coculture sample were normalized to the Cg internal control peak within the sample and response factors for each strain were applied (strain rfu/(Cg rfu × strain Rf)). Normalized individual strain data are expressed as a percentage of the sum of all strains in the sample and scaled to the OD of the original sample.

### Statistical analysis.

All analyses were done using R 3.6 (R Foundation for Statistical Computing, Vienna, Austria). Data on vitamin-restricted growth and on amino acid-restricted growth were analyzed by linear mixed models with random effect terms for the experiment (plate) and replicate within experiment and a fixed effect term for treatment. Treatment groups were compared with *post hoc* tests and Tukey’s adjustment for multiple comparisons. The effect of various vitamin concentrations was analyzed by linear model analysis of variance (ANOVA) with terms for plate and concentration, with concentrations compared with *post hoc* tests and Tukey’s adjustment for multiple comparisons.

Bacterial compositions of coculture experiments were compared by multivariate ANOVA for compositional data using an isometric log ratio transform. The absolute amounts of individual strains scaled to the culture OD were compared using linear models with terms for consortium and concentration. Consortium within concentration and vice versa were compared with *post hoc* tests and Tukey’s adjustment for multiple comparisons.

Standard errors were obtained from the pooled estimates of variance where replication was low and from individual group variances when it appeared adequate. For all variables, histograms of residuals were examined for normality, and where these appeared skewed, analysis on a log scale was used for *P* values and *post hoc* comparisons.
