# Supplementary material for: Vitamin Biosynthesis by Human Gut Butyrate-Producing Bacteria and Cross-Feeding in Synthetic Microbial Communities
Source: mBio. 2020 Jul 14;11(4):e00886-20. doi: 10.1128/mBio.00886-20 (PMC7360928; doi:10.1128/mBio.00886-20)

GENERAL EXPLANATIONS

- Precursor in a route; or in a branch or section of a route 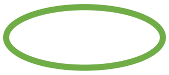
- Final product (cofactor) of the route 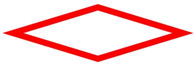
- Catalytic step 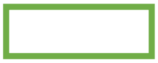

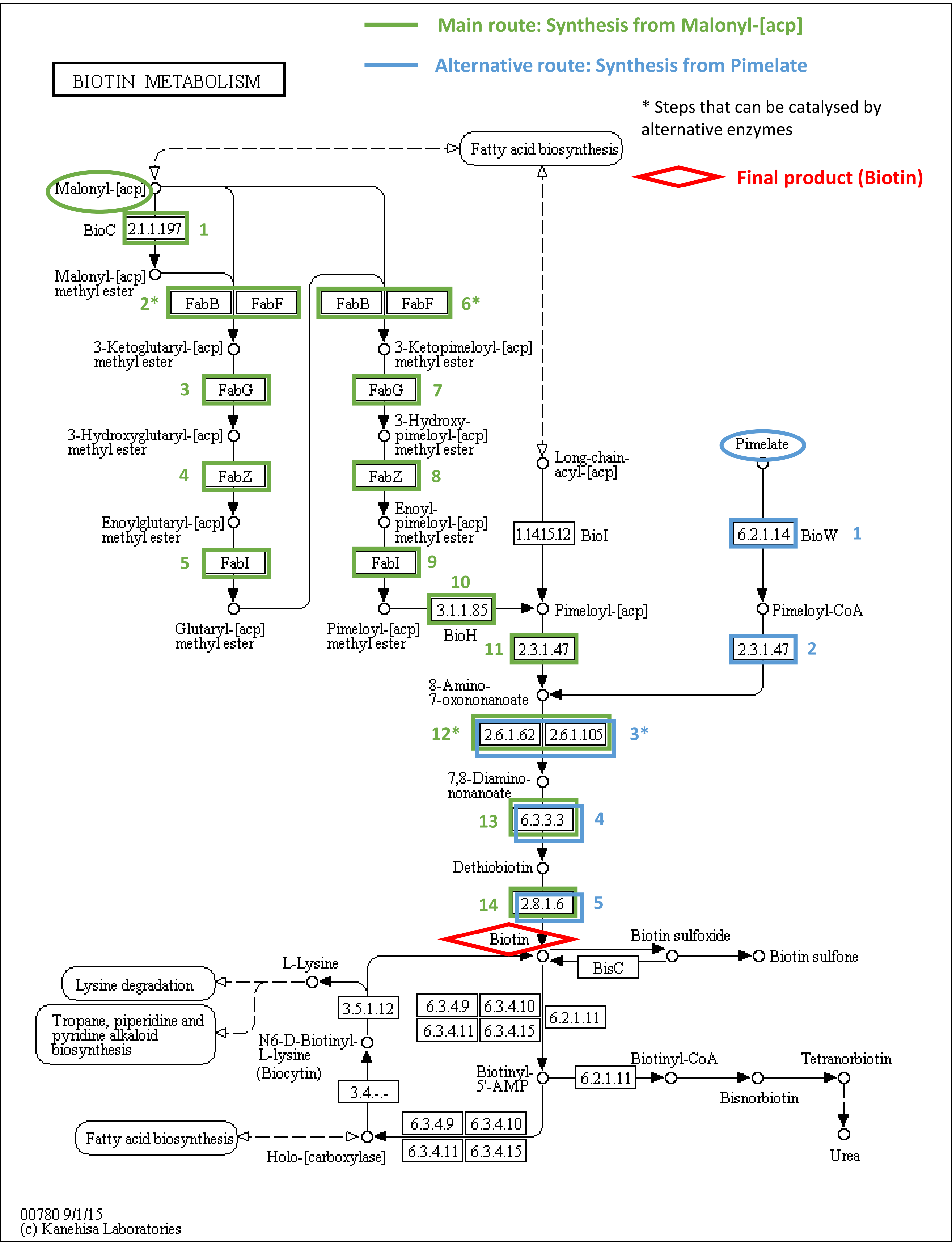
Main route: Synthesis from Malonyl-[acp]
Alternative route: Synthesis from Pimelate
BIOTIN METABOLISM
* Steps that can be catalysed by alternative enzymes
Final product (Biotin)
Fatty acid biosynthesis
Malonyl-[acp]
BioC
2.1.1.197
1
Malonyl-[acp] methyl ester
2*
FabB
FabF
FabB
FabF
6*
3-Ketoglutaryl-[acp] methyl ester
3-Ketopimeloyl-[acp] methyl ester
3
FabG
FabG
7
3-Hydroxyglutaryl-[acp] methyl ester
3-Hydroxy-pimeloyl-[acp] methyl ester
4
FabZ
FabZ
8
Long-chain-acyl-[acp]
Pimelate
Enoylglutaryl-[acp] methyl ester
Enoyl-pimeloyl-[acp] methyl ester
1.14.15.12
BioI
6.2.1.14
BioW
1
5
FabI
FabI
9
10
3.1.1.85
Pimeloyl-[acp]
Pimeloyl-CoA
Glutaryl-[acp] methyl ester
Pimeloyl-[acp] methyl ester
BioH
11
2.3.1.47
2.3.1.47
2
8-Amino-7-oxononanoate
12*
2.6.1.62
2.6.1.105
3*
7,8-Diamino-nonanoate
13
6.3.3.3
4
Dethiobiotin
14
2.8.1.6
5
Biotin
Biotin sulfoxide
Biotin sulfone
BisC
L-Lysine
Lysine degradation
Tropane, piperidine and pyridine alkaloid biosynthesis
3.5.1.12
6.3.4.9
6.3.4.10
6.3.4.11
6.3.4.15
6.2.1.11
N6-D-Biotinyl-L-lysine (Biocytin)
Biotinyl-5'-AMP
6.2.1.11
Biotinyl-CoA
Tetranorbiotin
Bisnorbiotin
Urea
3.4.-.-
Fatty acid biosynthesis
6.3.4.9
6.3.4.10
6.3.4.11
6.3.4.15
Holo-[carboxylase]
00780 9/1/15
(c) Kanehisa Laboratories

Branch 1: Synthesis of Adenosyl cobyrinate hexaamide

- Section 1: Formation of ALA
- Section 2: Formation of Adenosyl cobyrinate hexaamide

Branch 2: Transformation of Adenosyl cobyrinate hexaamide into Adenosine-GDP-cobinamide

- Section 1: Formation of Adenosyl cobinamide phosphate
- Section 2: Transformation of Adenosyl cobinamide phosphate to Adenosine-GDP-cobinamide

Branch 3: Synthesis of α-Ribazole from 5-Aminoimidazole ribotide (based on Hazra et al., PNAS 112: 10792 (2015)

Branch 4: Merge of Adenosine-GDP-cobinamide with α-Ribazole to form B12 coenzyme

Final product (Adenosylcobalamin – B12 coenzyme)

ADENOSYLCOBALAMIN PATHWAY

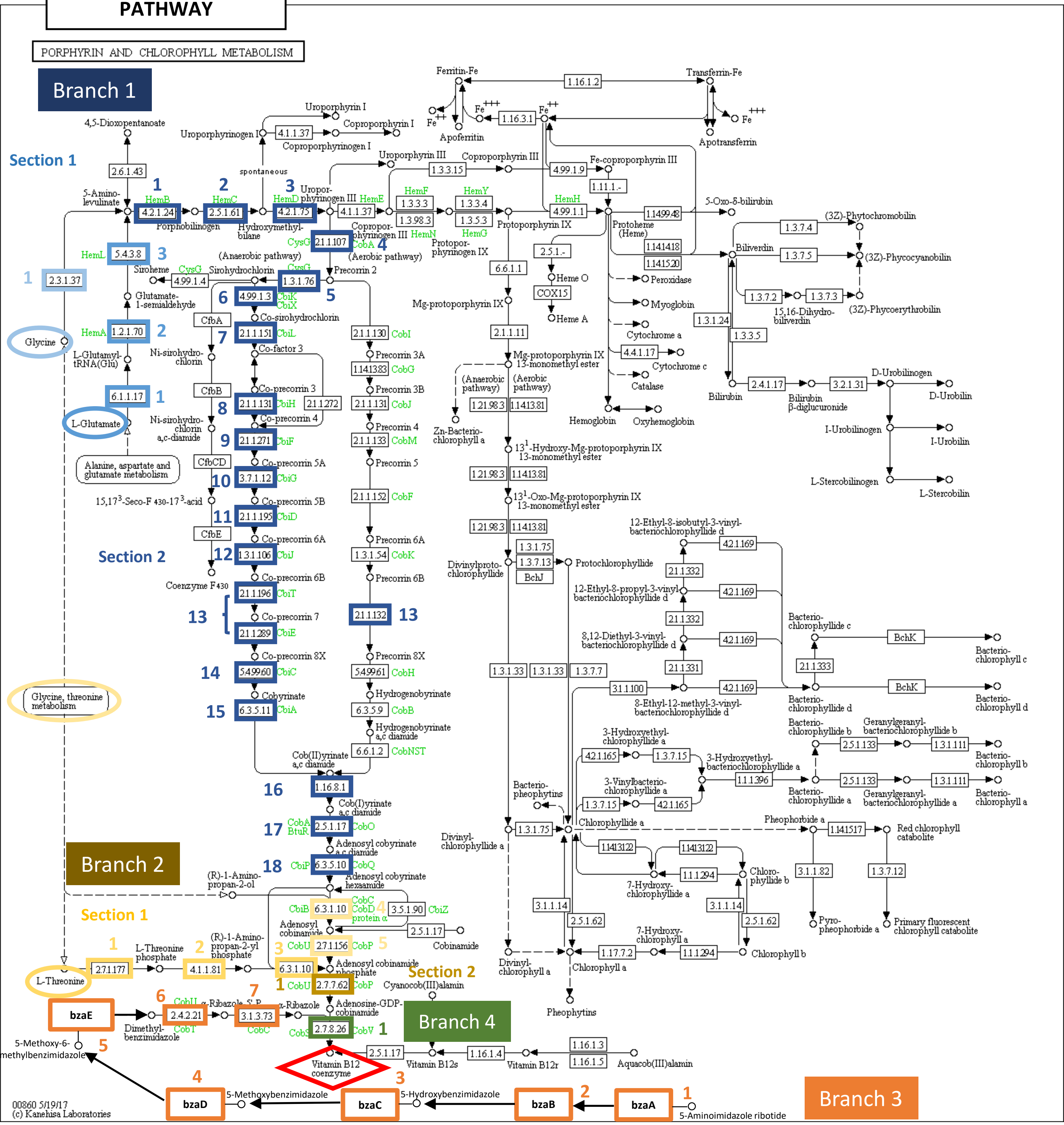

Branch 1: Synthesis of 2-Amino-4-hydroxyl-6-hydroxymethyl-7,8-dihydropteridine

Branch 2: Synthesis of 4-Aminobenzoate

Branch 3: Merge of final compounds from branches 1 and 2 and synthesis of DHF

Final product (THF)

* Steps that can be catalysed by alternative enzymes

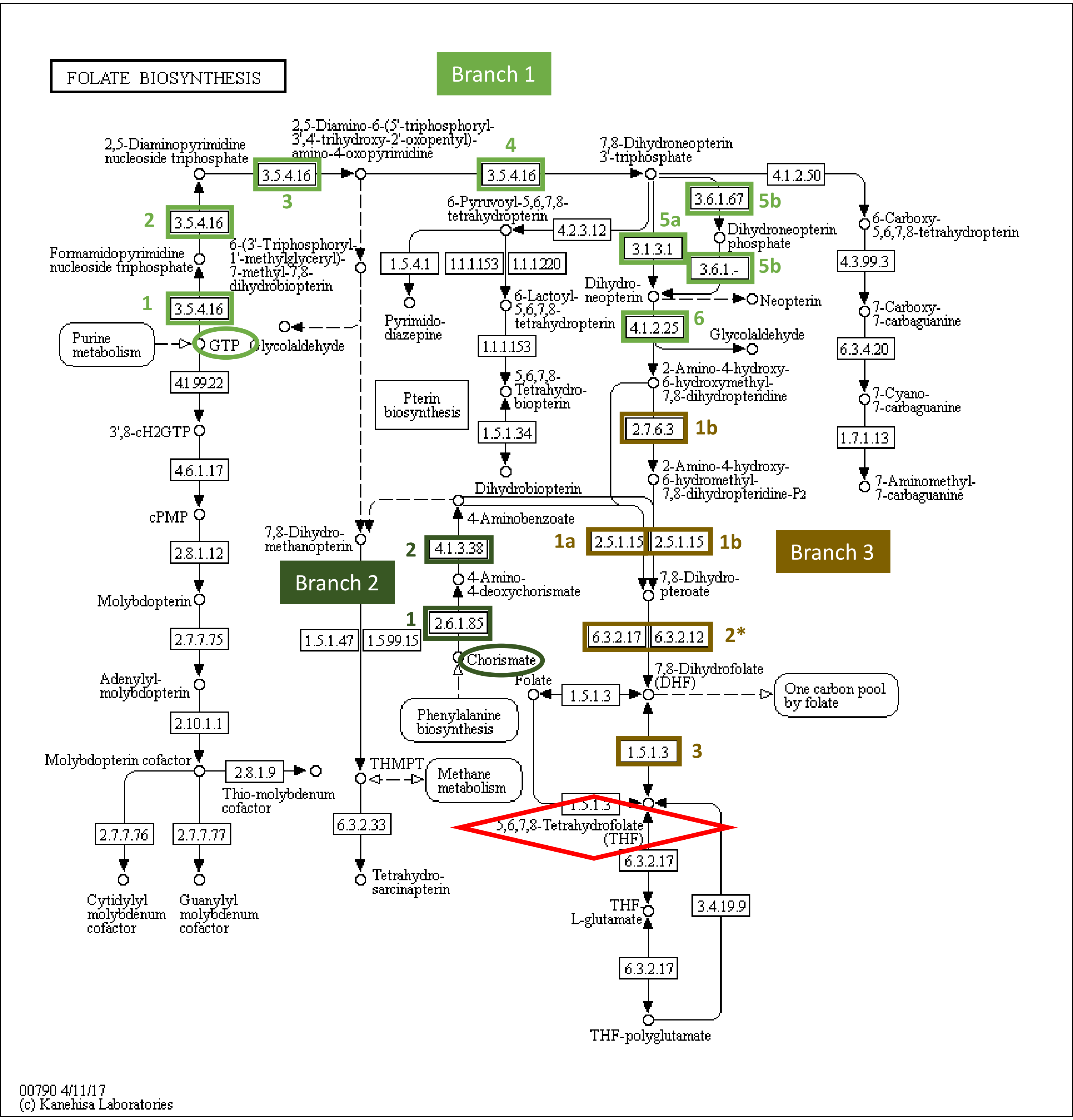

De novo route: Synthesis from L-Aspartate

Alternative route: Synthesis via Quinolinate produced from Tryptophan metabolism

Salvage route 1: Synthesis from Nicotinamide

Salvage route 2: Synthesis from Nicotinate

Final product (NAD+)

* Steps that can be catalysed by alternative enzymes

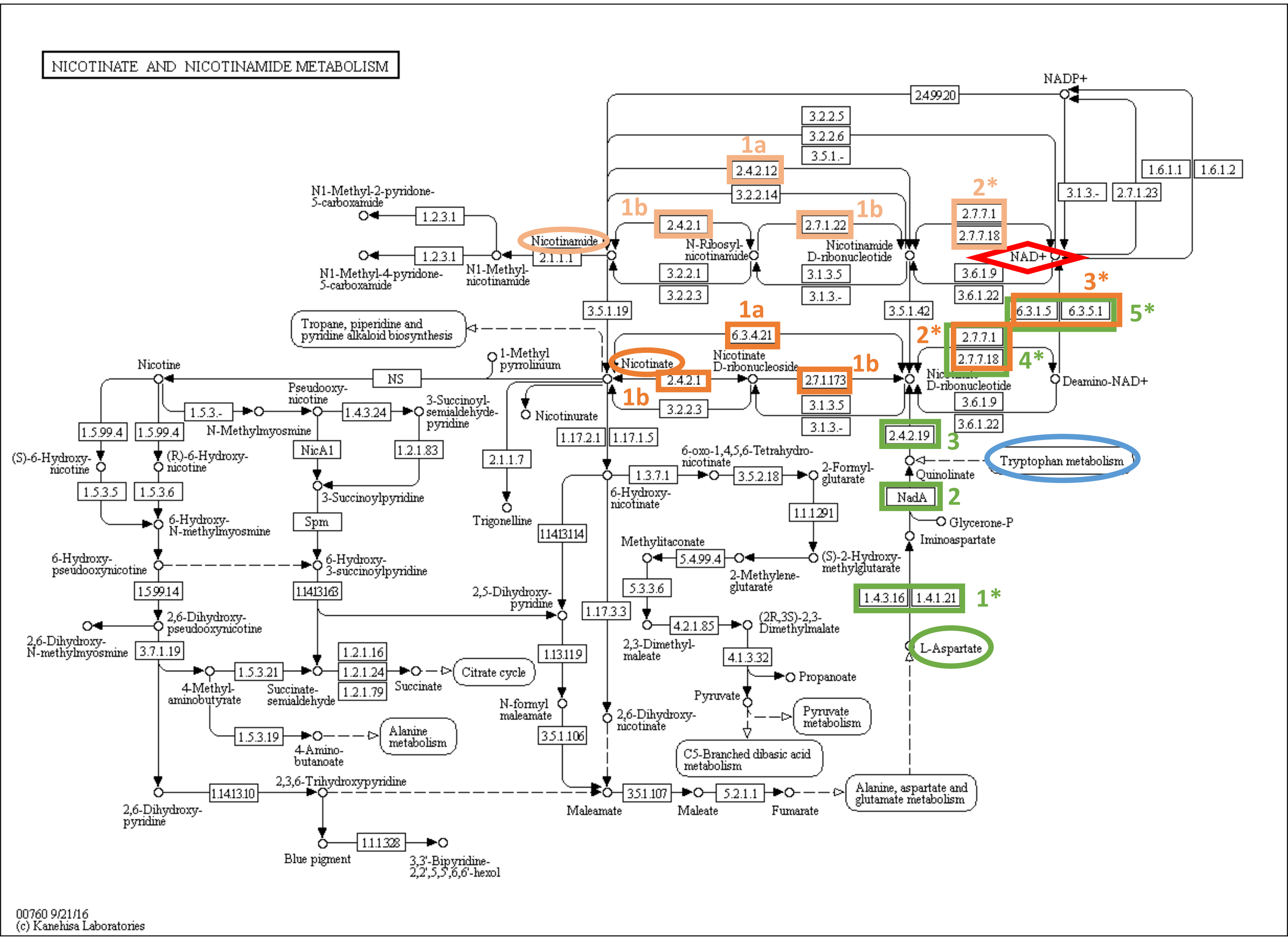

Branch 1: Synthesis of (R)-Pantoate

Section 1: Synthesis of 3-Methyl-2-oxobutanoate (from Pyruvate or from L-Valine)

Section 2: Conversion of 3-Methyl-2-oxobutanoate to (R)-Pantoate

Branch 2: Synthesis of β-Alanine

Alternative a: From L-Aspartate

Alternative b: From Uracil

Alternative c: From Propanoate metabolism

Branch 3: Merge of (R)-Pantoate and β-Alanine

Section 1: Synthesis of (R)-4'-Phospho-pantothenate

Section 2: Merge of D-4'-Phospho-pantothenate with L-Cysteine to produce CoA

Salvage route: Use of Pantetheine

Alternative A: Shortcut route through 4'-Phospho-pantetheine

Alternative B: Route through (R)-Pantothenate

Final product (Coenzyme A)

* Steps that can be catalysed by alternative enzymes

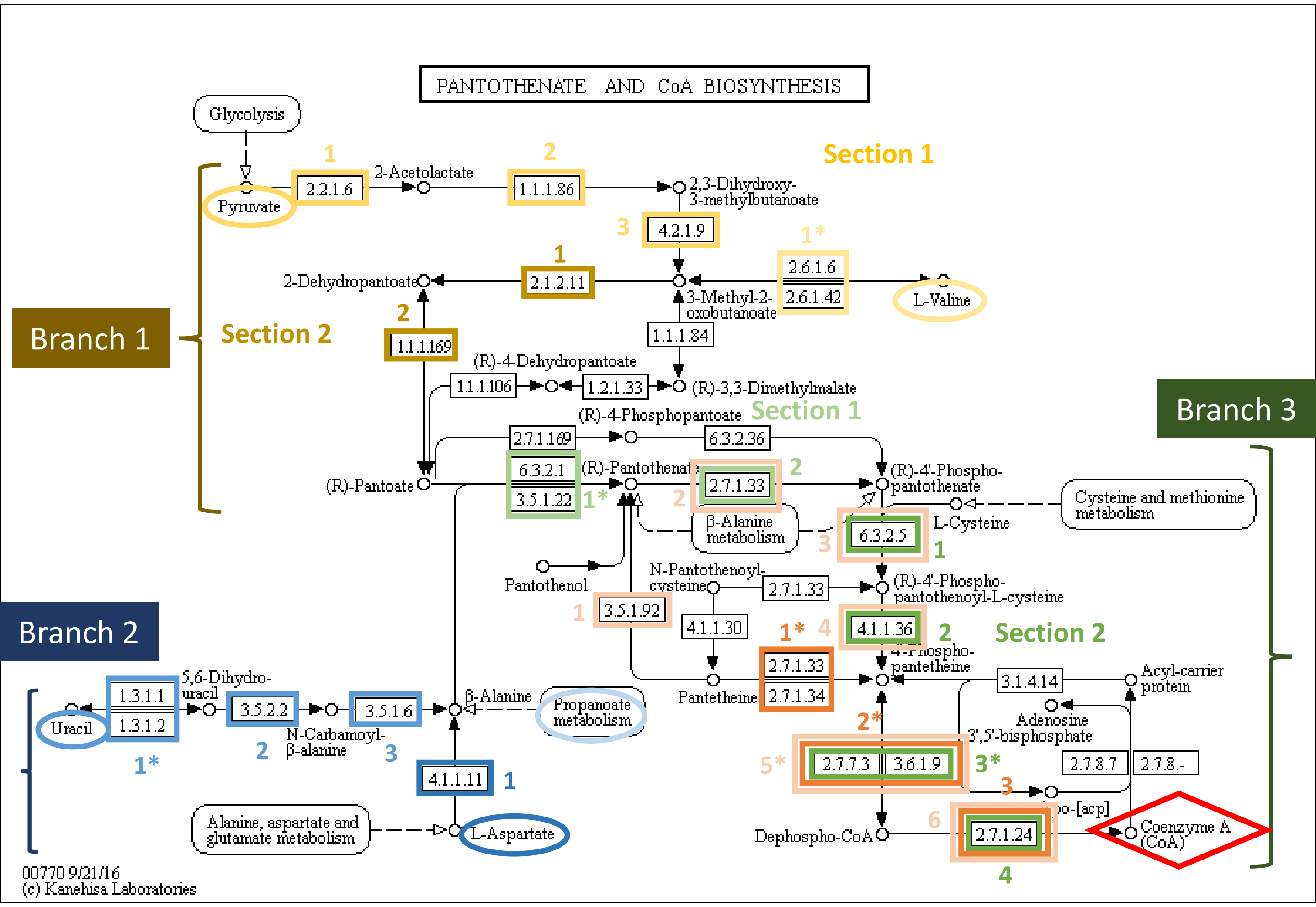

Main route: Synthesis from D-Erythrose 4-phosphate

Alternative route: Synthesis from Ribulose 5-phosphate + Glyceraldehyde 3-phosphate

Salvage route 1: Synthesis from Pyridoxamine

Salvage route 2: Synthesis from Pyridoxal

Salvage route 3: Synthesis from Pyridoxine

Final product (Pyridoxal 5-phosphate)

* Steps that can be catalysed by alternative enzymes

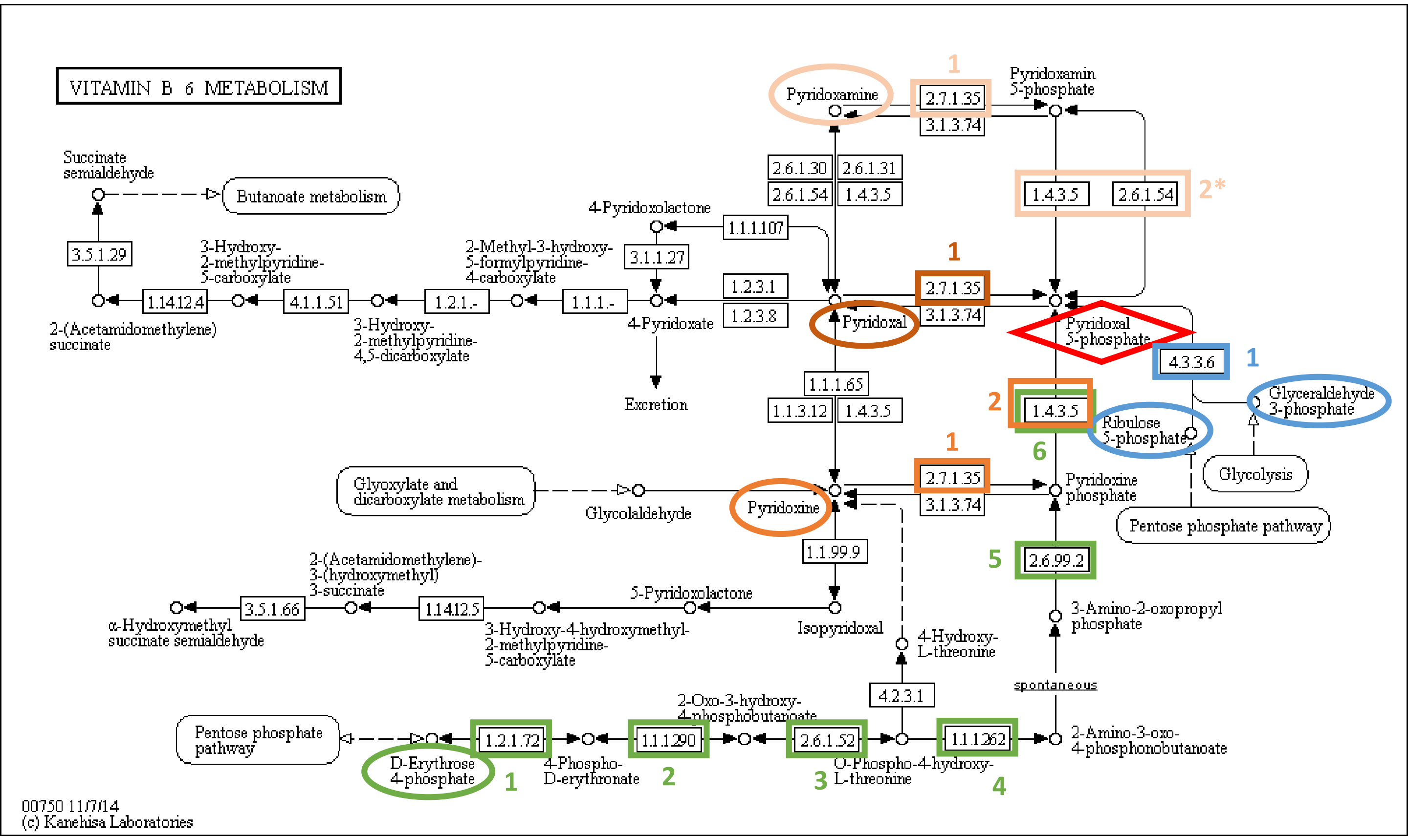

Branch 1: Synthesis of 5-Amino-6-(ribityl-amino)uracil

Branch 2: Synthesis of 3,4-Dihydroxy-2-butanone-4-phosphate

Branch 3: Merge of final compounds from branches 1 and 2 and synthesis of FMN^{+} and FAD^{+}

Final products (FMN^{+} and FAD^{+})

* Steps that can be catalysed by alternative enzymes

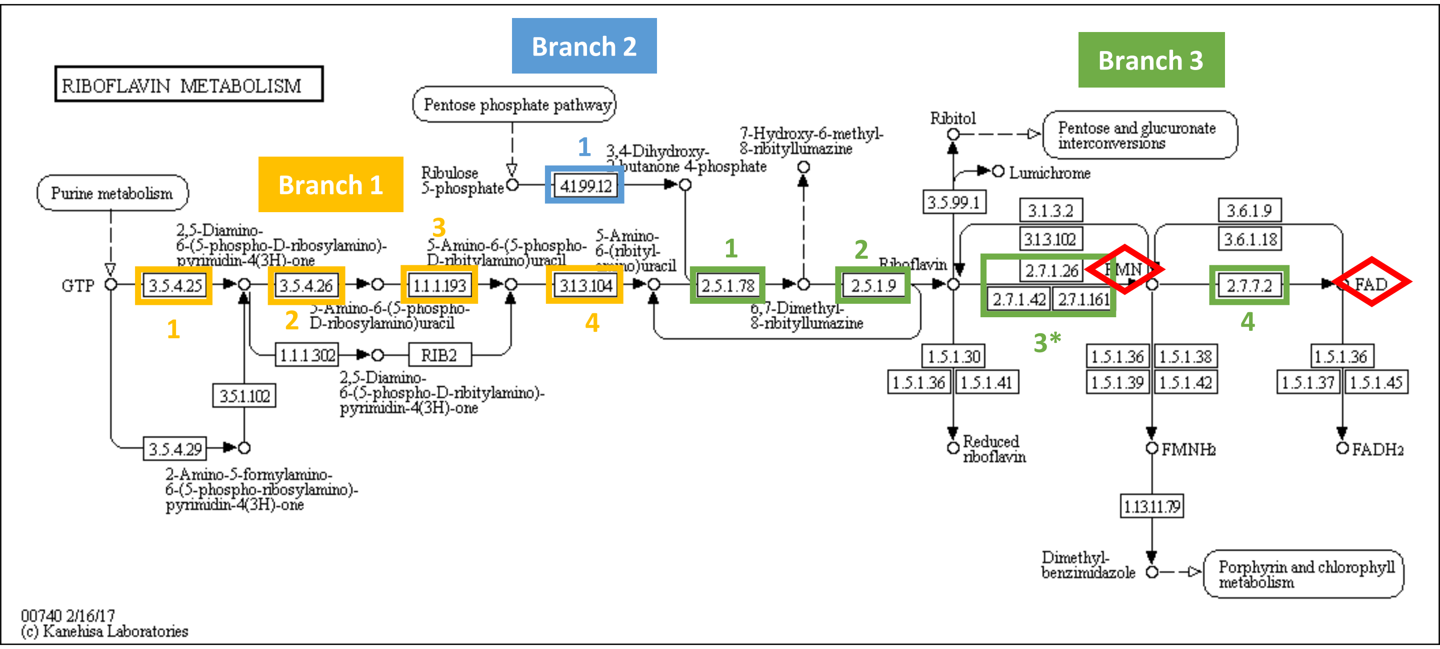

Branch 1: Synthesis of 4-Amino-5-hydroxymethyl-2-methylpyrimidine diphosphate

Branch 2: Synthesis of 2-(2-Carboxy-4-methylthiazol-5-yl)ethyl phosphate

Section 1: Synthesis of Thiocarboxy-[sulfur-carrier protein]

Section 2: Synthesis of 1-Deoxy-D-Xylulose 5-phosphate

Section 3: Synthesis of Iminoglycine

Section 4: Merge of final products from sections 1, 2 and 3 and synthesis of 2-(2-Carboxy-4-methylthiazol-5-yl)ethyl phosphate

Branch 3: Merge of final compounds from branches 1 and 2 and synthesis of Thiamine diphosphate

Final product (Thiamine diphosphate)

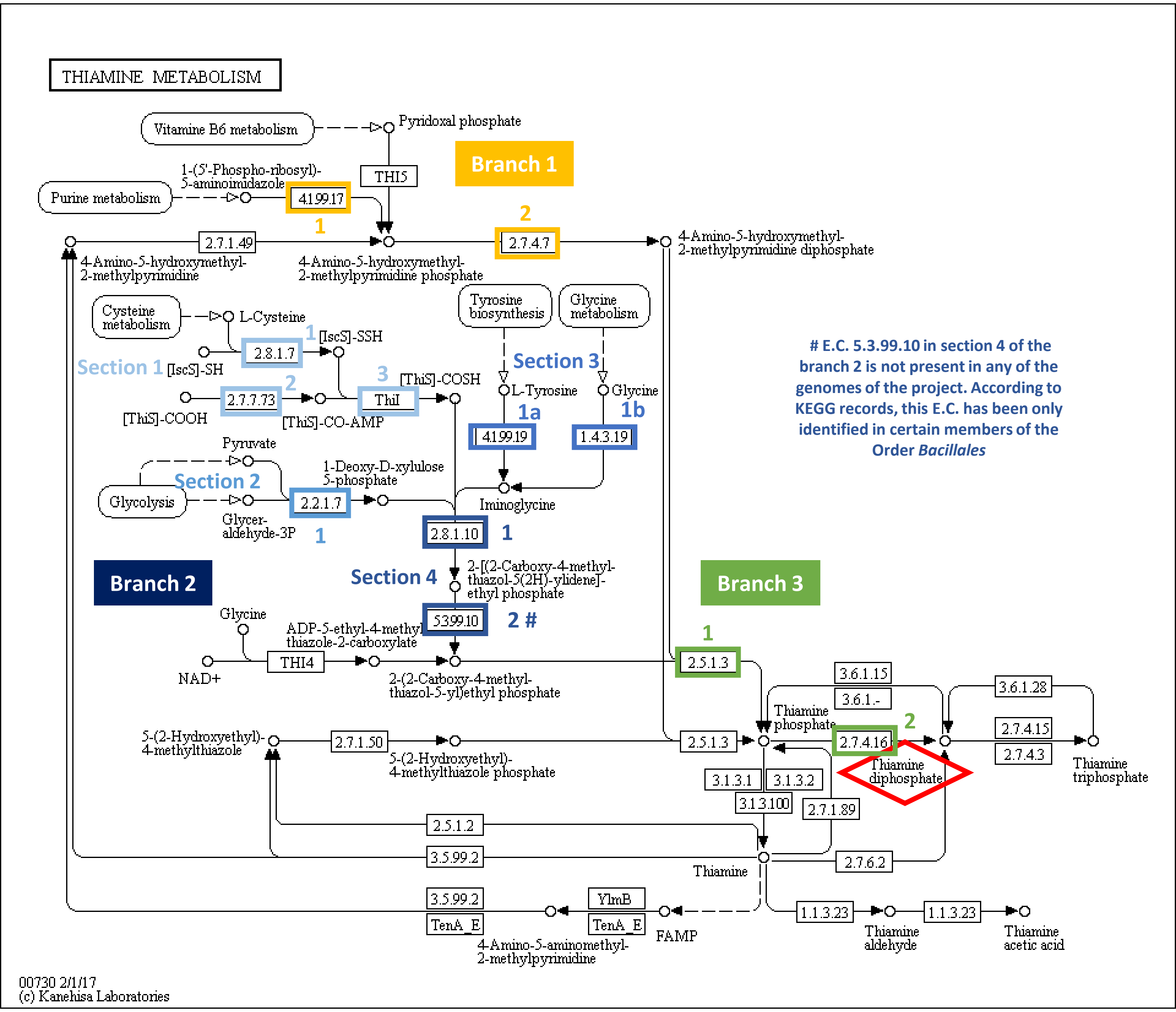

Supplement: FIG S1 [file mBio.00886-20-sf001.pdf]
