# Supplementary material for: Vitamin Biosynthesis by Human Gut Butyrate-Producing Bacteria and Cross-Feeding in Synthetic Microbial Communities
Source: mBio. 2020 Jul 14;11(4):e00886-20. doi: 10.1128/mBio.00886-20 (PMC7360928; doi:10.1128/mBio.00886-20)
Supplement: FIG S2 [file mBio.00886-20-sf002.pdf]

GENERAL EXPLANATIONS

- Enzymes with pink background belong to a pre-defined KEGG module.

- Enzymes highlighted with a frame of a non-pink colour (green, purple/violet, blue, orange/brown) belong to a module defined based on Wendisch VF (ed). *Amino acid biosynthesis – Pathways, regulation and metabolic engineering*. Microbiology Monographs. Springer-Verlag: Berlin Heidelberg, Germany, 2007.

- Final product (amino acid) of the route 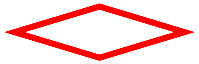

- Intermediate metabolite of the route 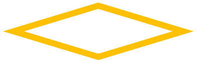

Alanine biosynthesis

* Aspartate -> alanine
* Pyruvate -> alanine (3 E.C. can do this conversion)

Aspartate biosynthesis

* Asparagine -> aspartate (3 E.C. can do this conversion)
* Oxaloacetate -> aspartate

Asparagine biosynthesis

* Aspartate -> asparagine (2 E.C. can do this conversion

Glutamate biosynthesis

* 2-Oxoglutarate -> glutamate (3 E.C. can do this conversion)
* Glutamine -> glutamate (2 E.C. can do this conversion)
* 2-Oxoglutarate + glutamine -> Glutamate (2 E.C. can do this conversion)

Glutamine biosynthesis

* Glutamate -> glutamine
* Glutamate + NH3 -> glutamine (2 E.C. can do this conversion)

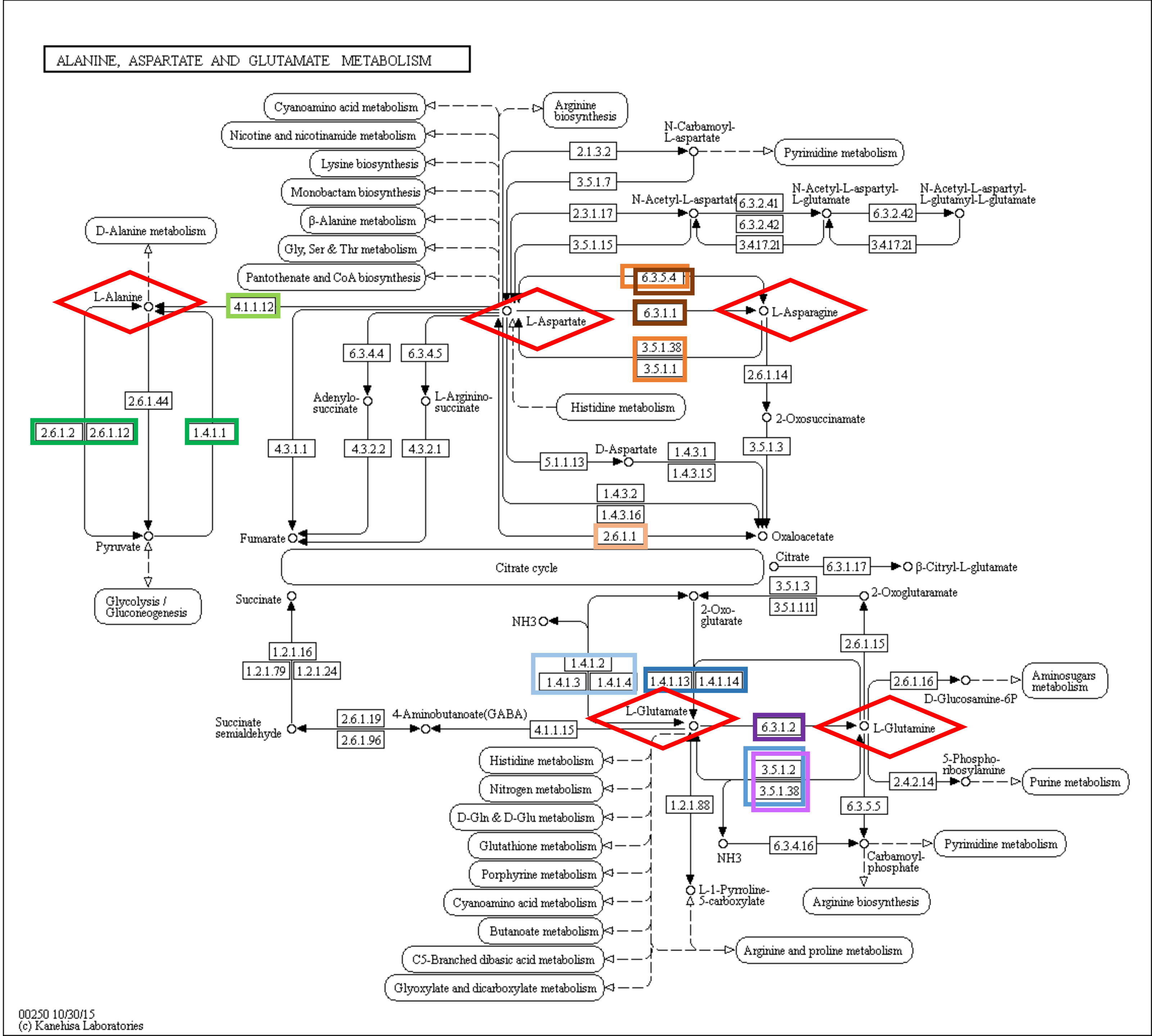

Glycine biosynthesis – 3 alternative modules:

Serine -> glycine

Threonine -> glycine

Choline -> glycine (unlikely route in bacteria)

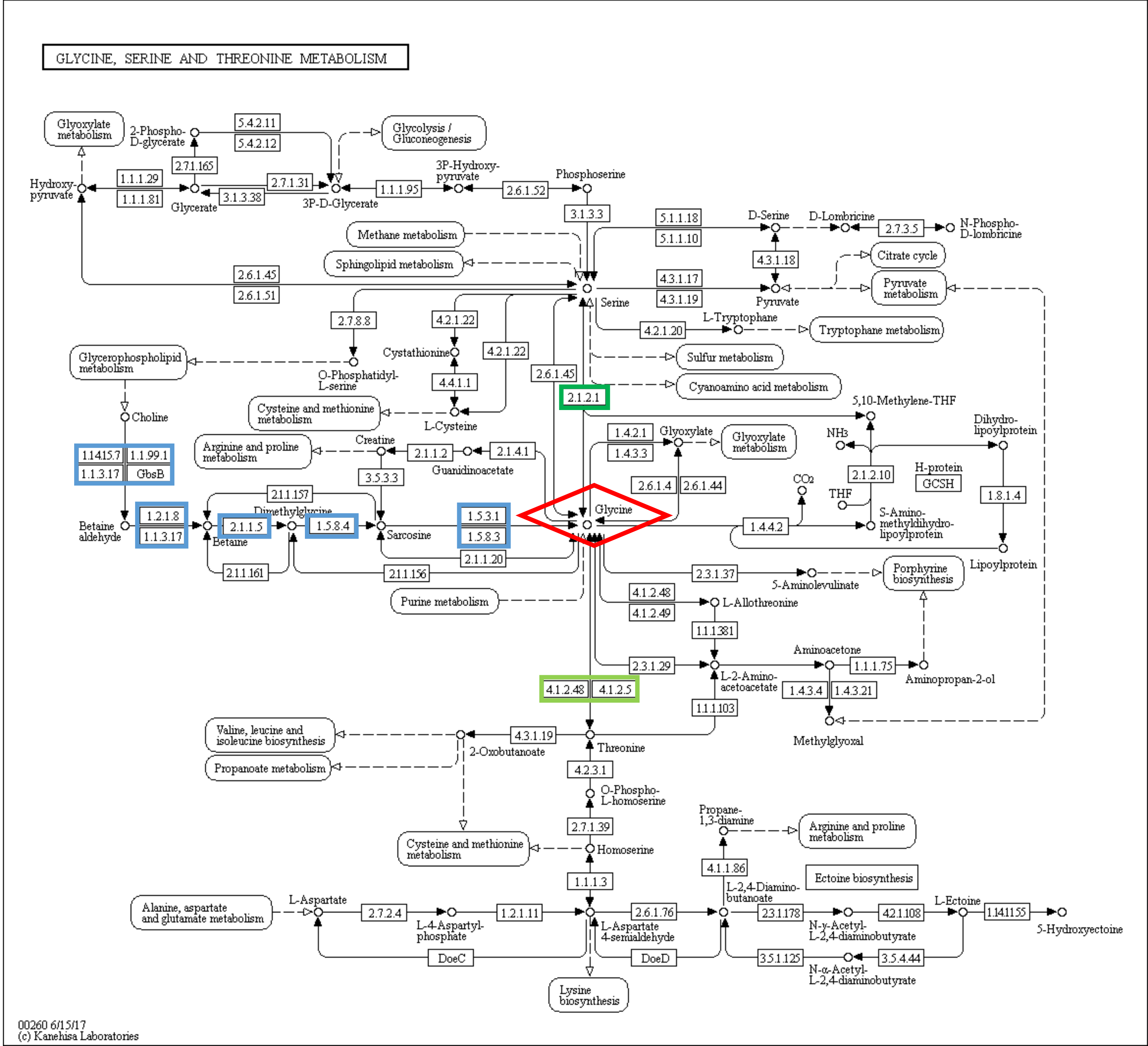

Serine biosynthesis - 2 alternative modules:

M00020: glycerate-3P => serine

pyruvate => serine

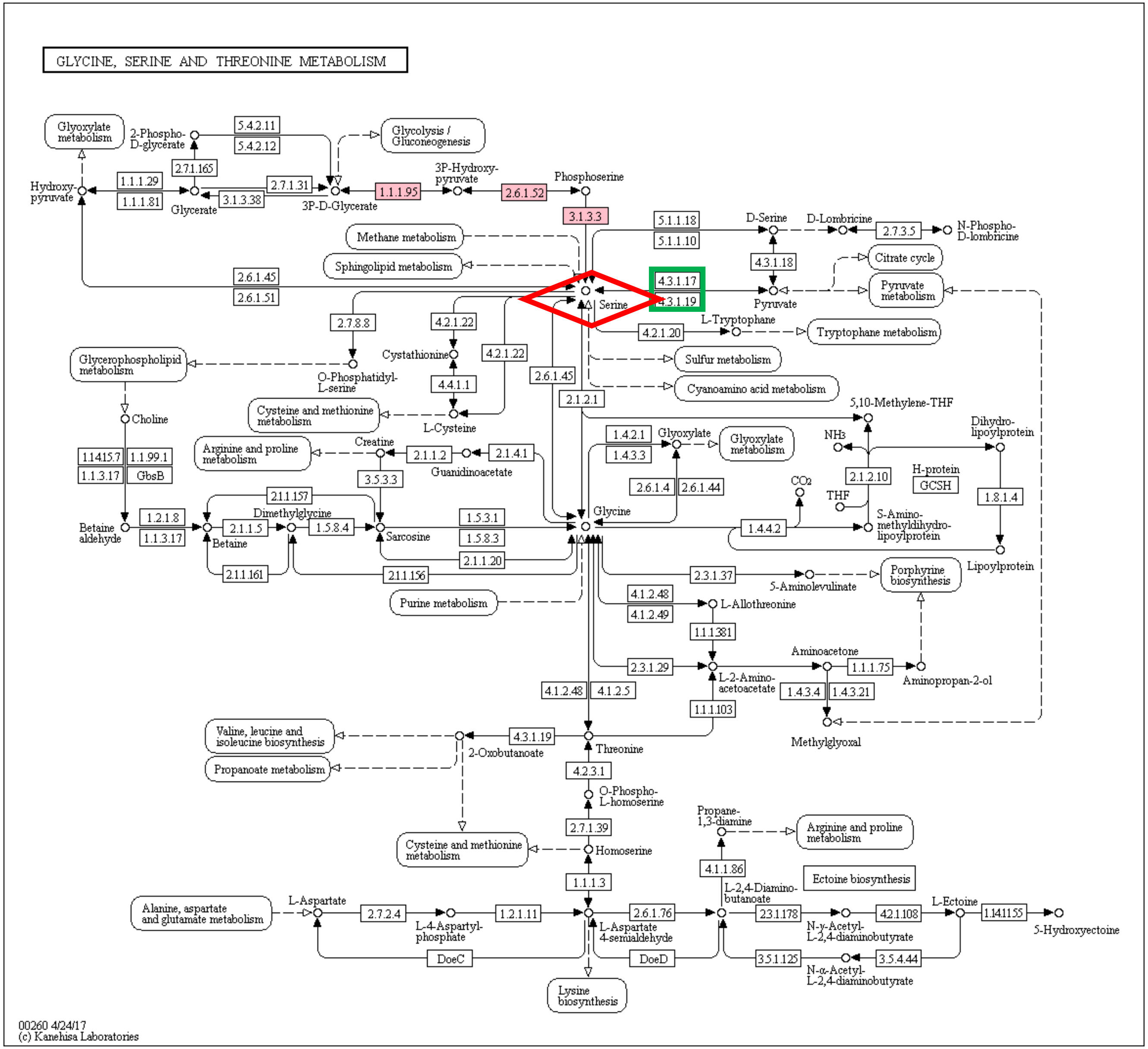

Threonine biosynthesis – 1 KEGG module

M00018: aspartate => homoserine => threonine

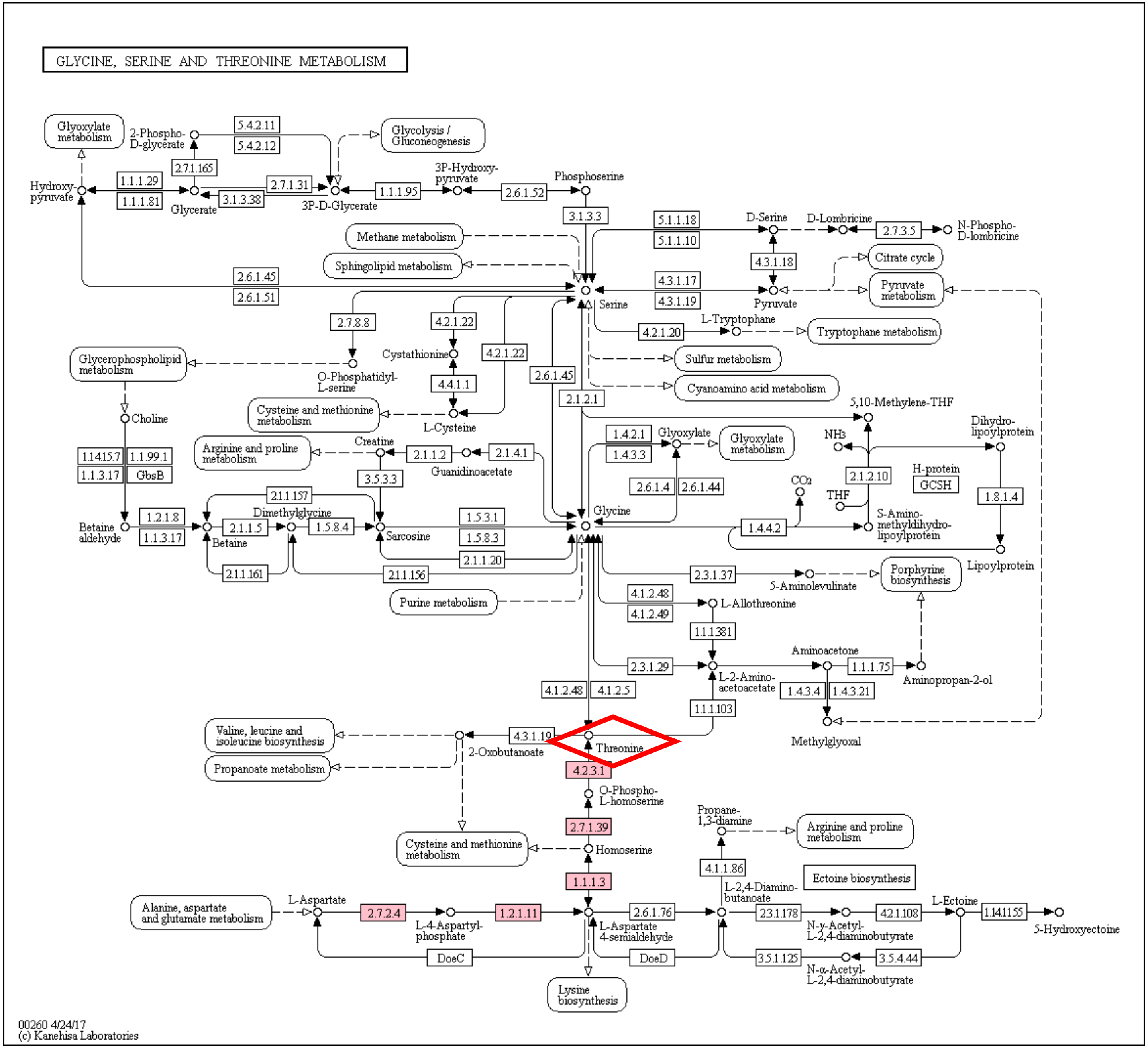

Cysteine biosynthesis – 3 alternative modules defined by KEGG

Module A

M00021: serine => cysteine

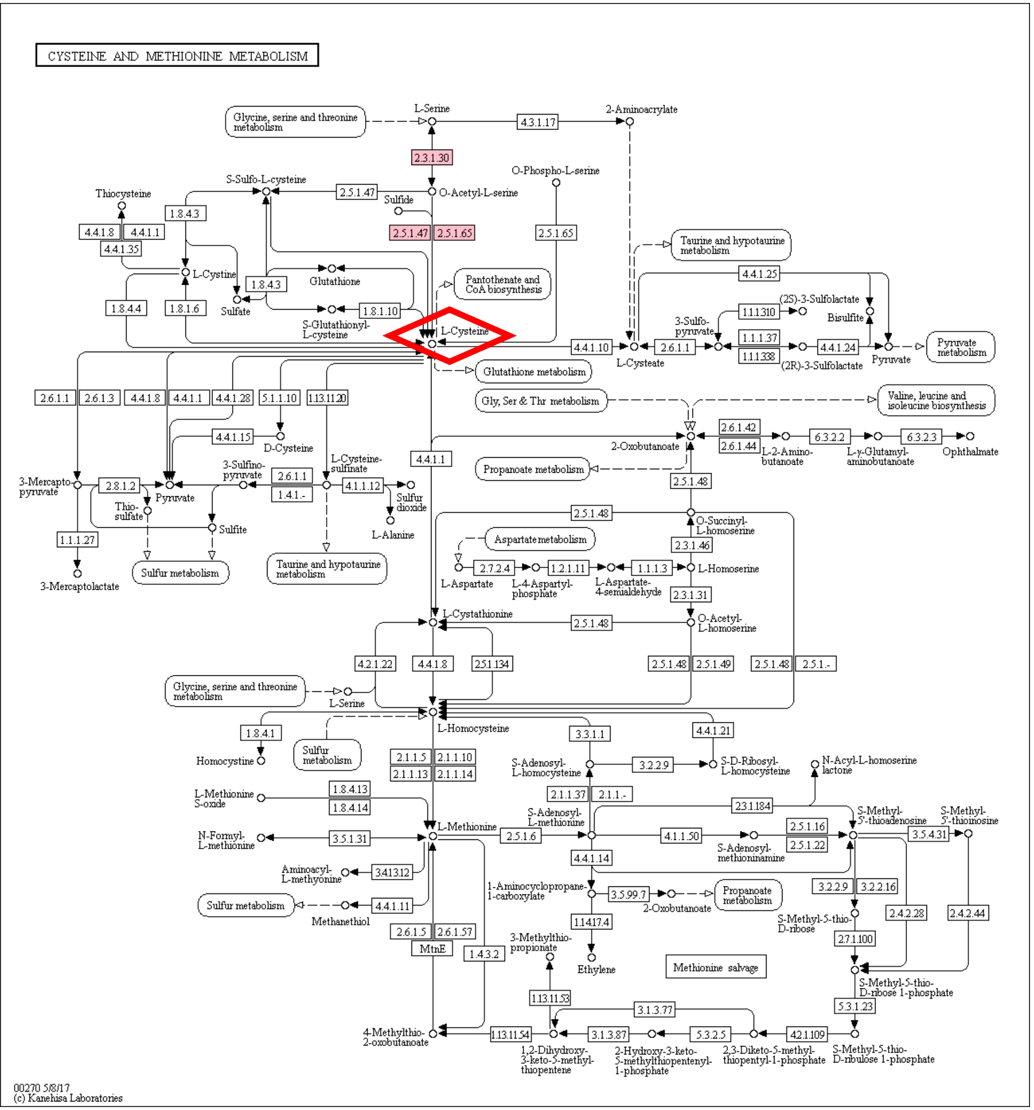

Module B

M00338: homocysteine + serine => cysteine

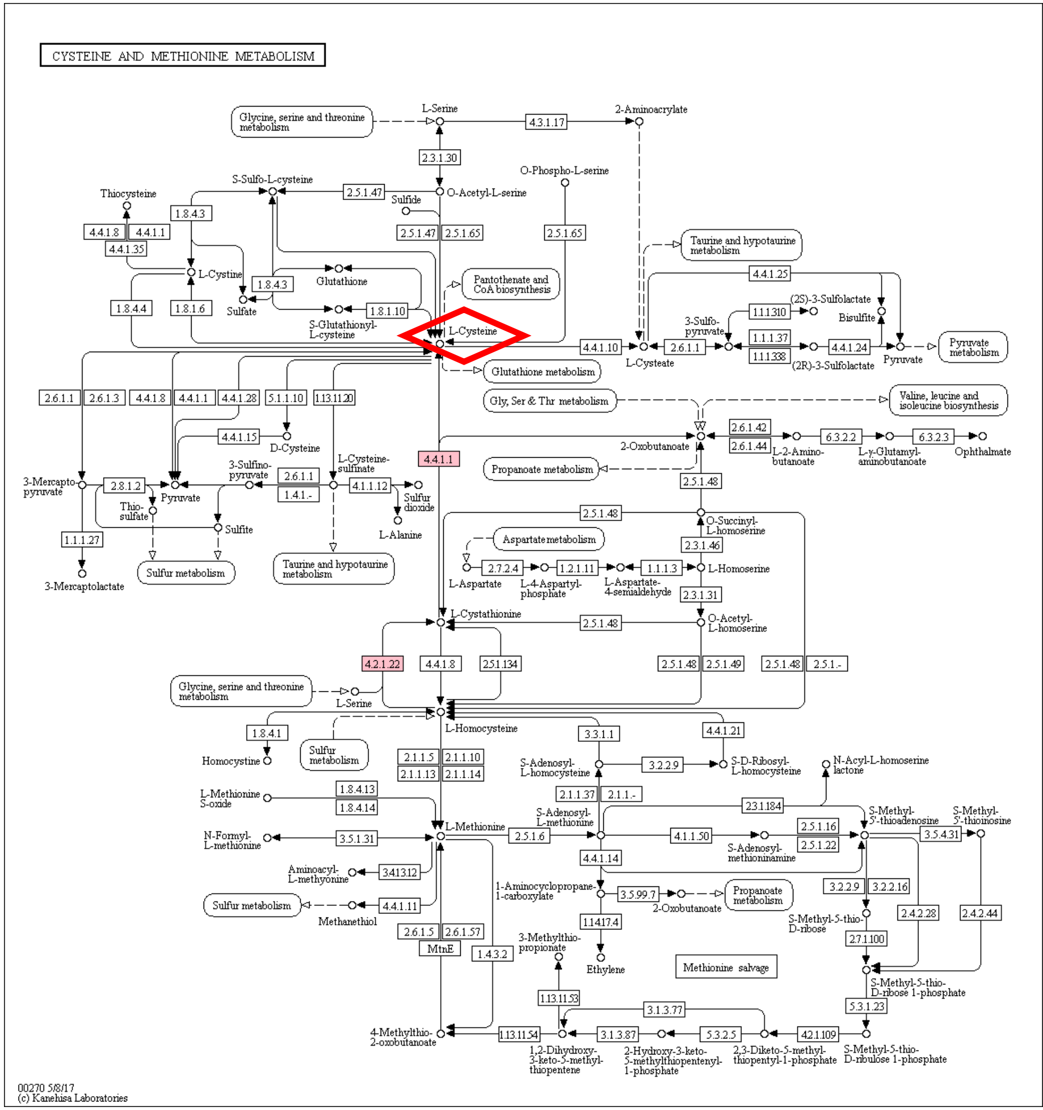

Cysteine biosynthesis – 3 alternative modules defined by KEGG

Module C

M00609: methionine => cysteine

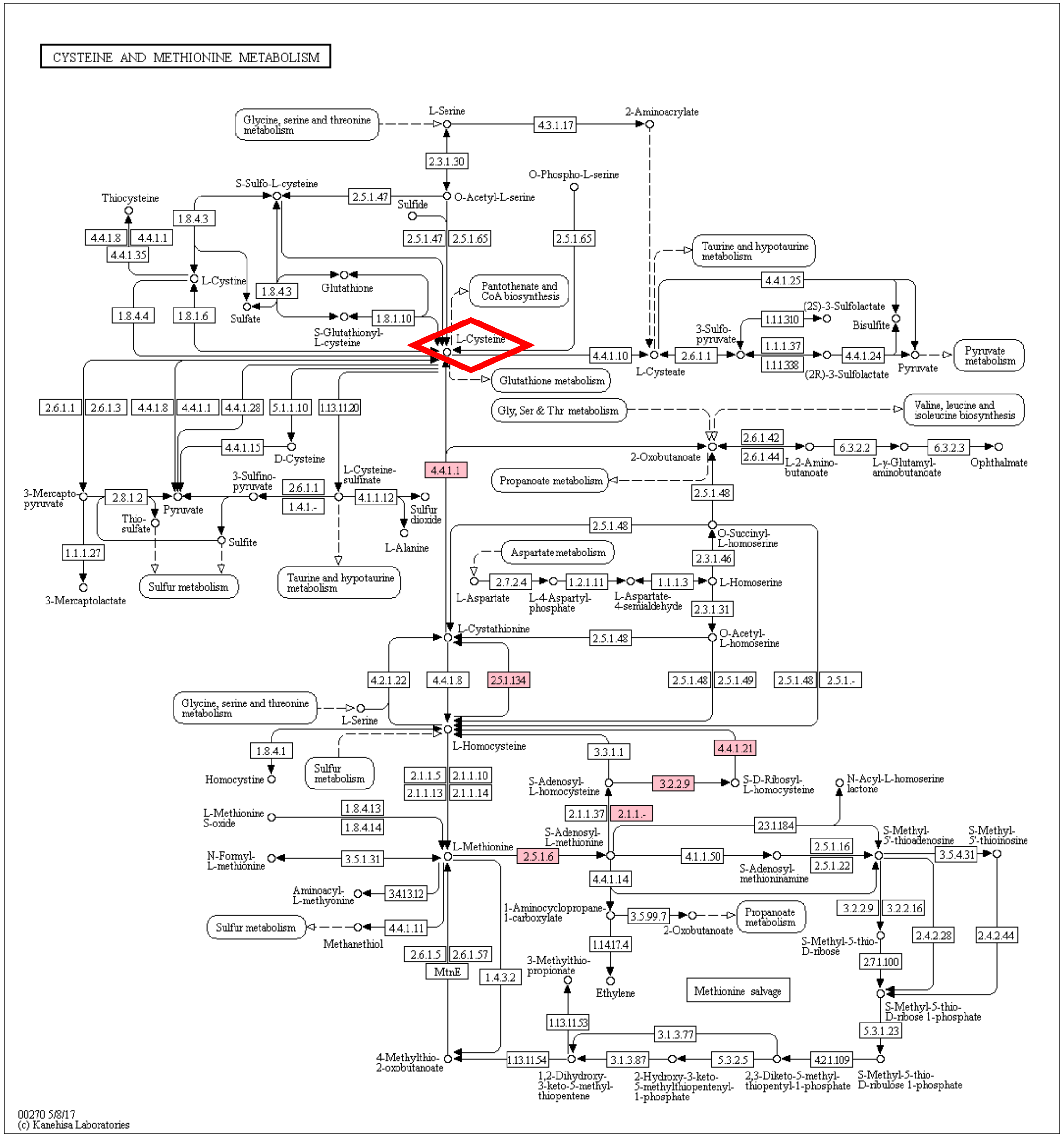

Methionine biosynthesis – 1 KEGG module

M0017: aspartate => homoserine => methionine

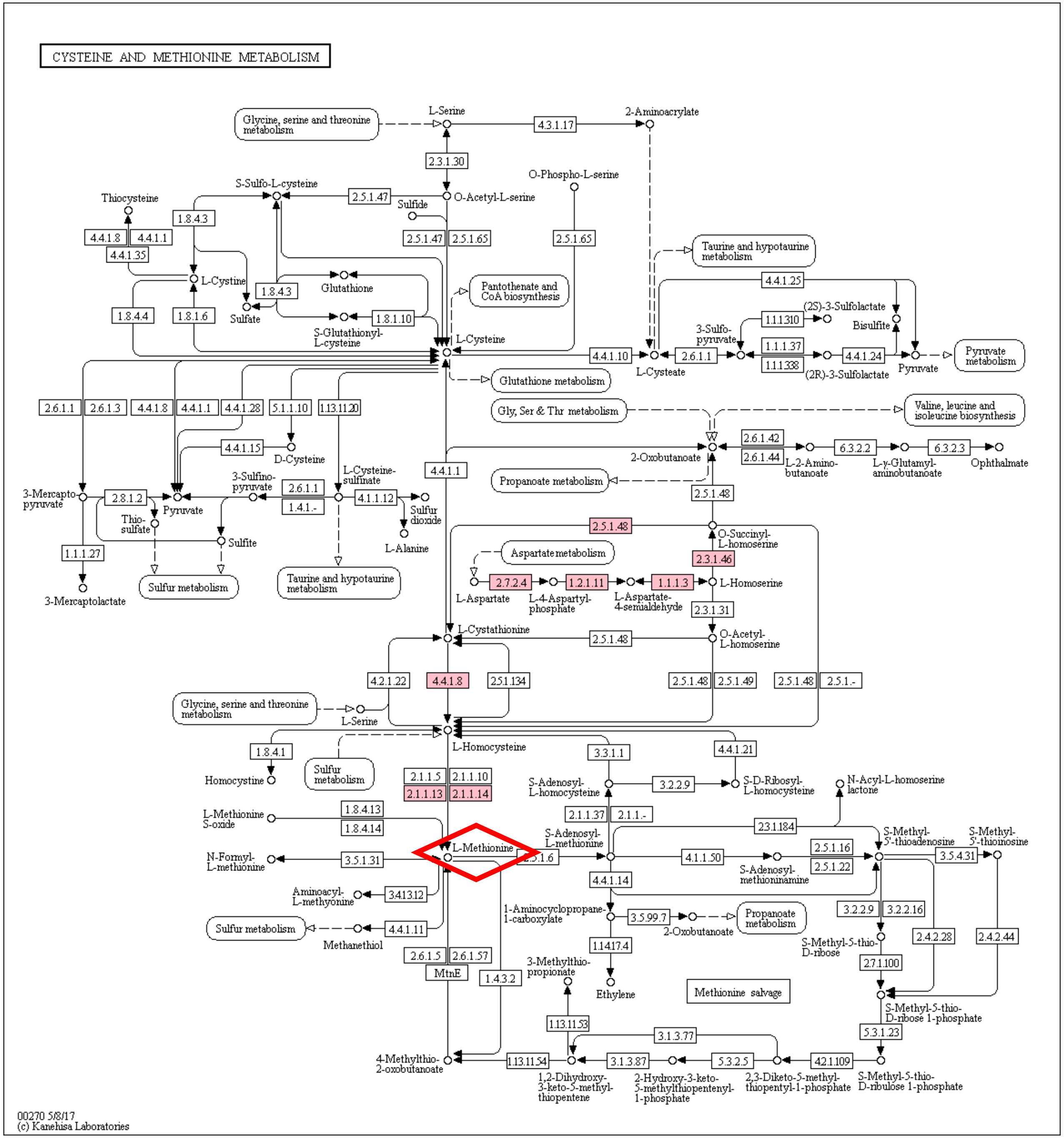

Valine biosynthesis – 1 KEGG module

M00019: pyruvate => valine

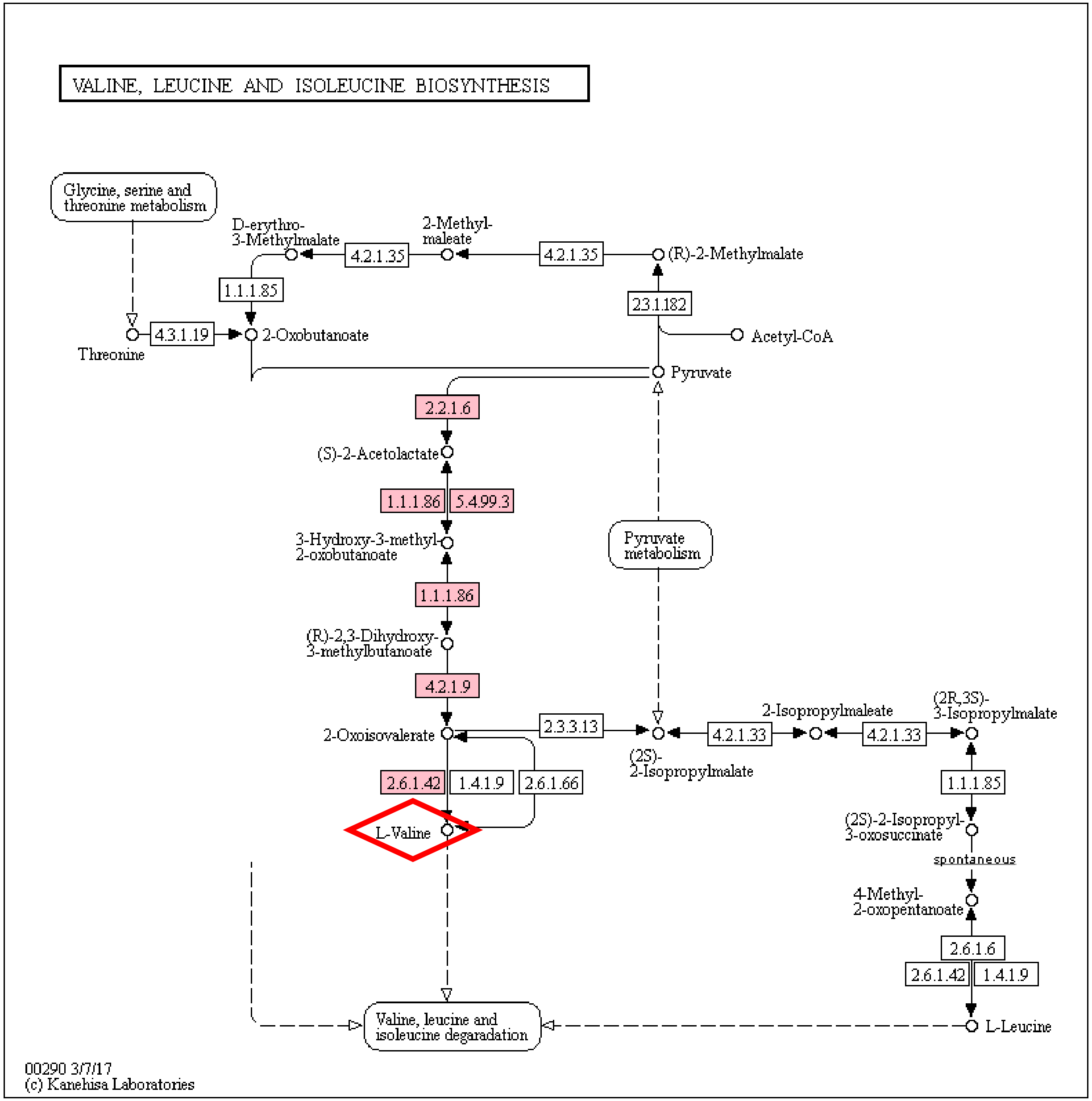

Leucine biosynthesis – 1 KEGG module

M00432: 2-oxoisovalerate => 2-oxoisocaproate (4-Methyl-2-oxopentanoate).

2-oxoisocaproate => L-leucine

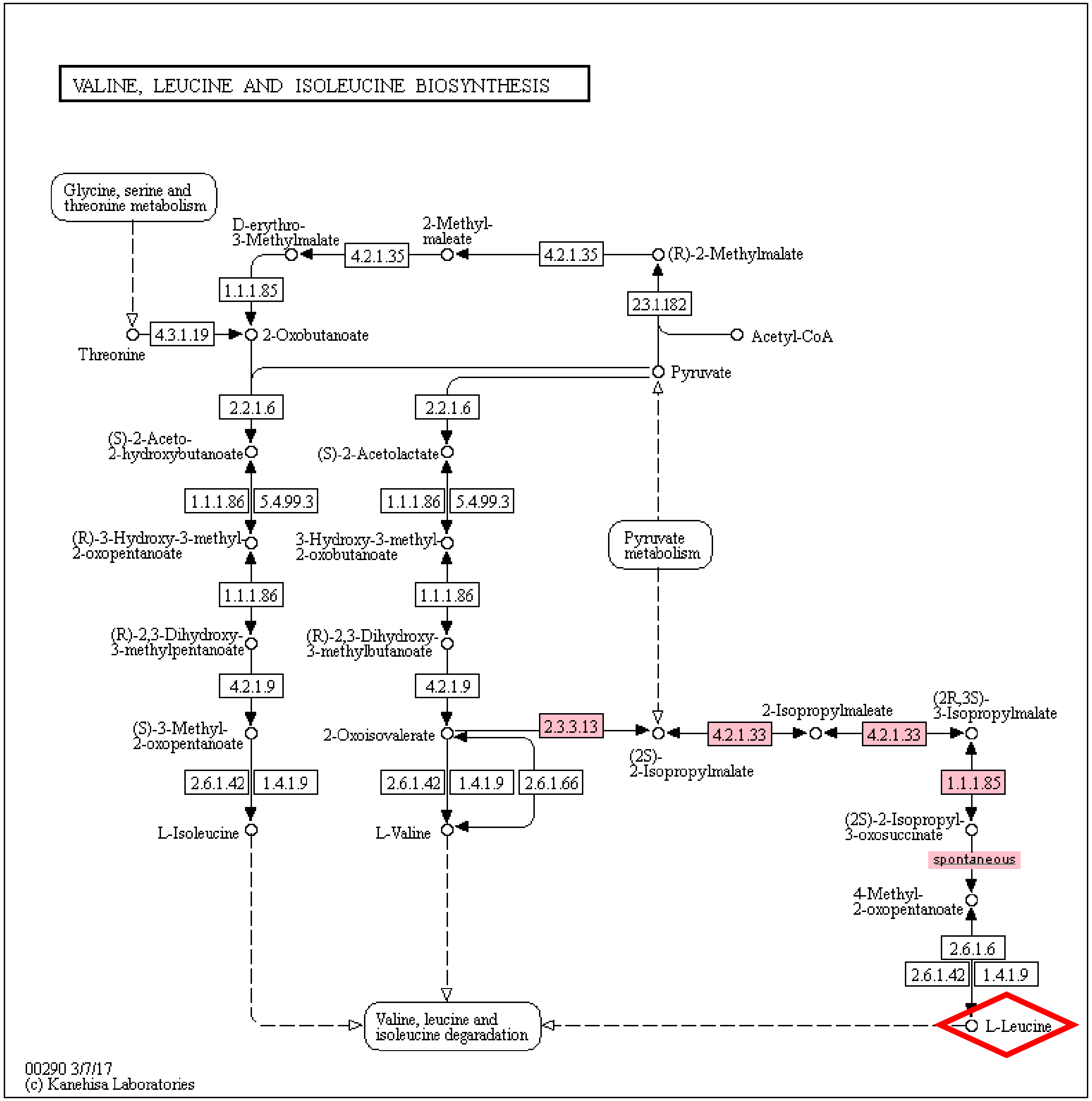

Isoleucine biosynthesis – 2 KEGG modules are needed

Module 1: two alternative ways to produce 2-oxobutanoate

A M00535: pyruvate => 2-oxobutanoate

B threonine => 2-oxobutanoate

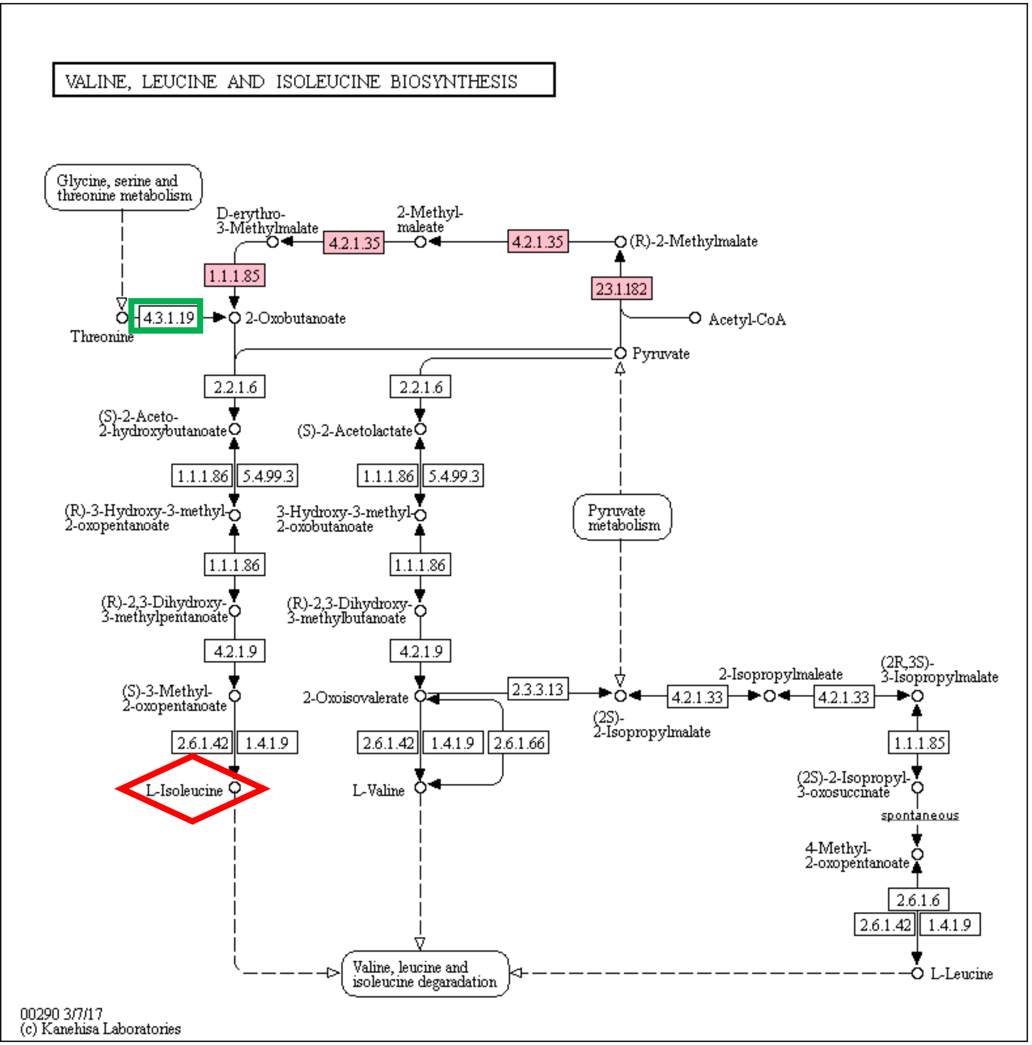

Module 2

M00570: 2-oxobutanoate => isoleucine

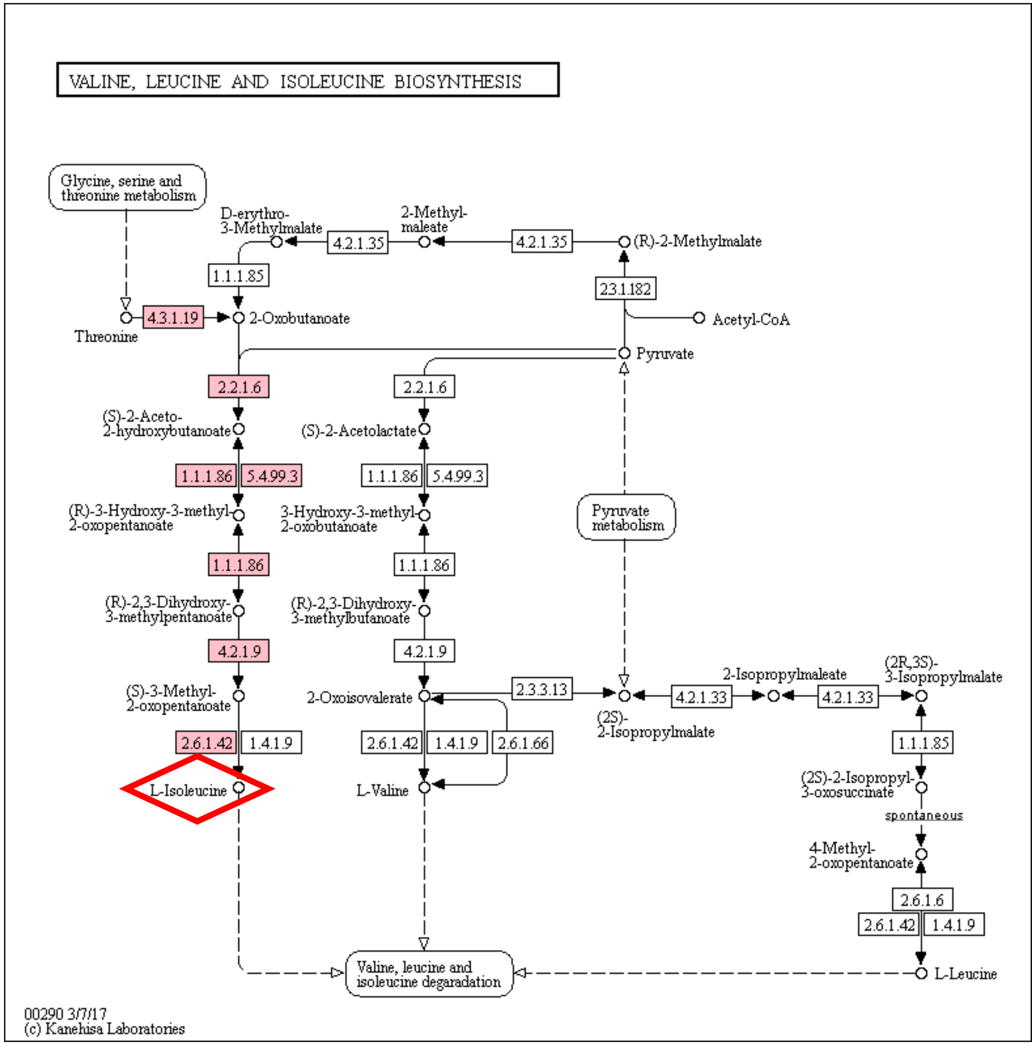

Lysine biosynthesis – 6 alternative modules defined by KEGG

Module B

M00525: acetyl-DAP pathway, aspartate => lysine

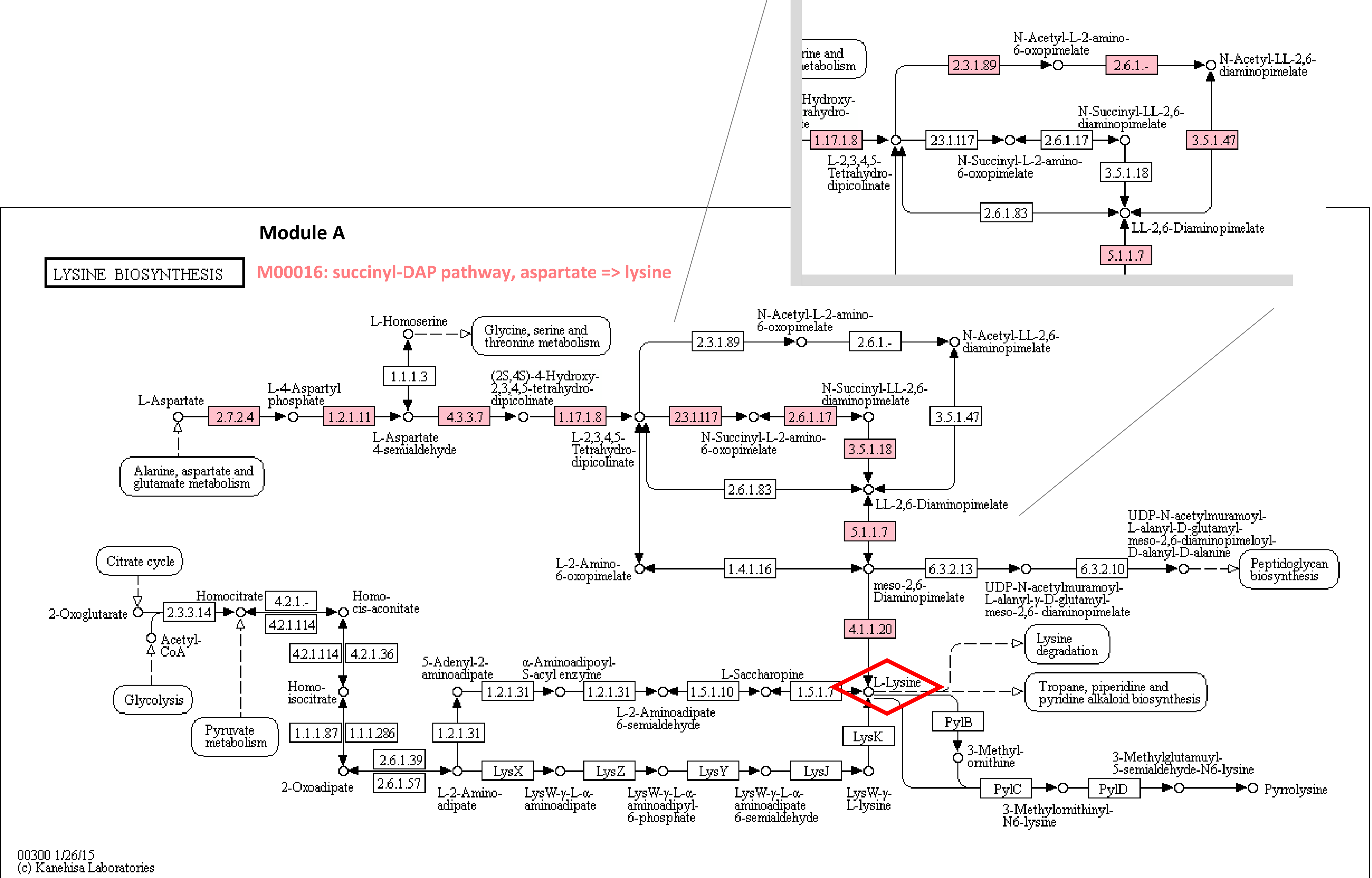

Lysine biosynthesis – 6 alternative modules defined by KEGG

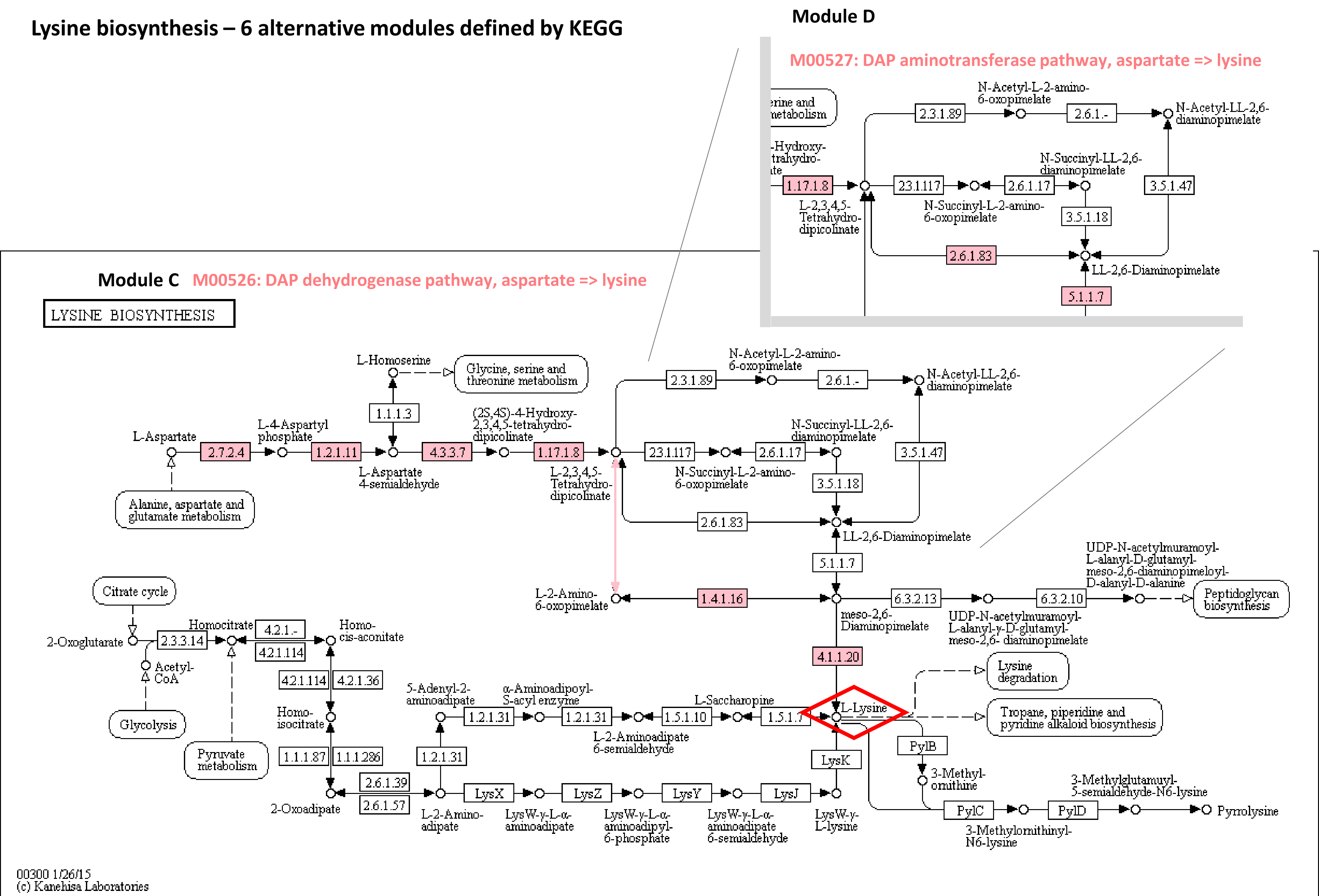

Lysine biosynthesis – 6 alternative modules defined by KEGG

Module E

M00030: AAA pathway, 2-oxoglutarate => 2-aminoadipate => lysine

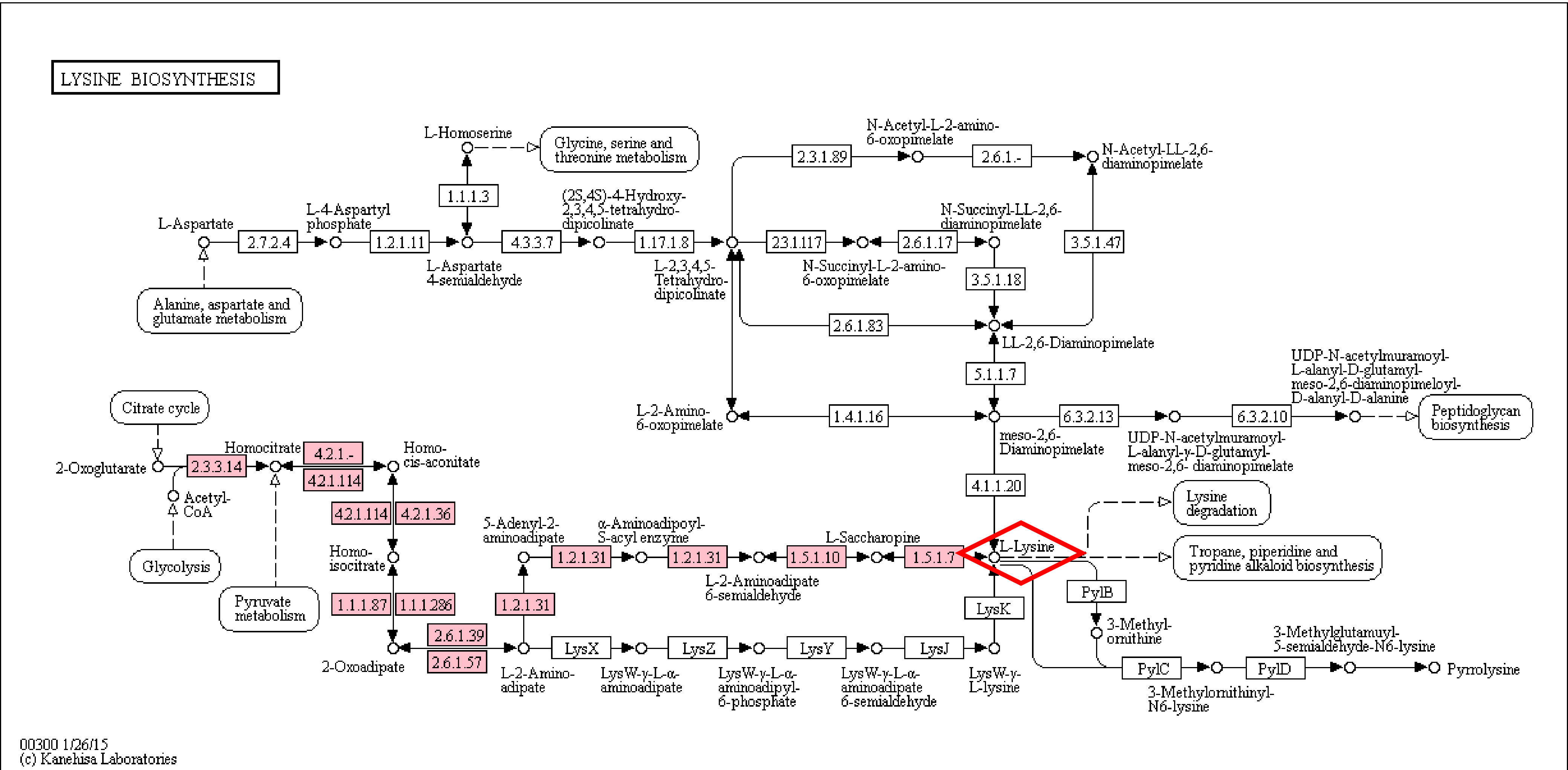

Lysine biosynthesis – 6 alternative modules defined by KEGG

Module F, first section

M00433: 2-oxoglutarate => 2-oxoadipate

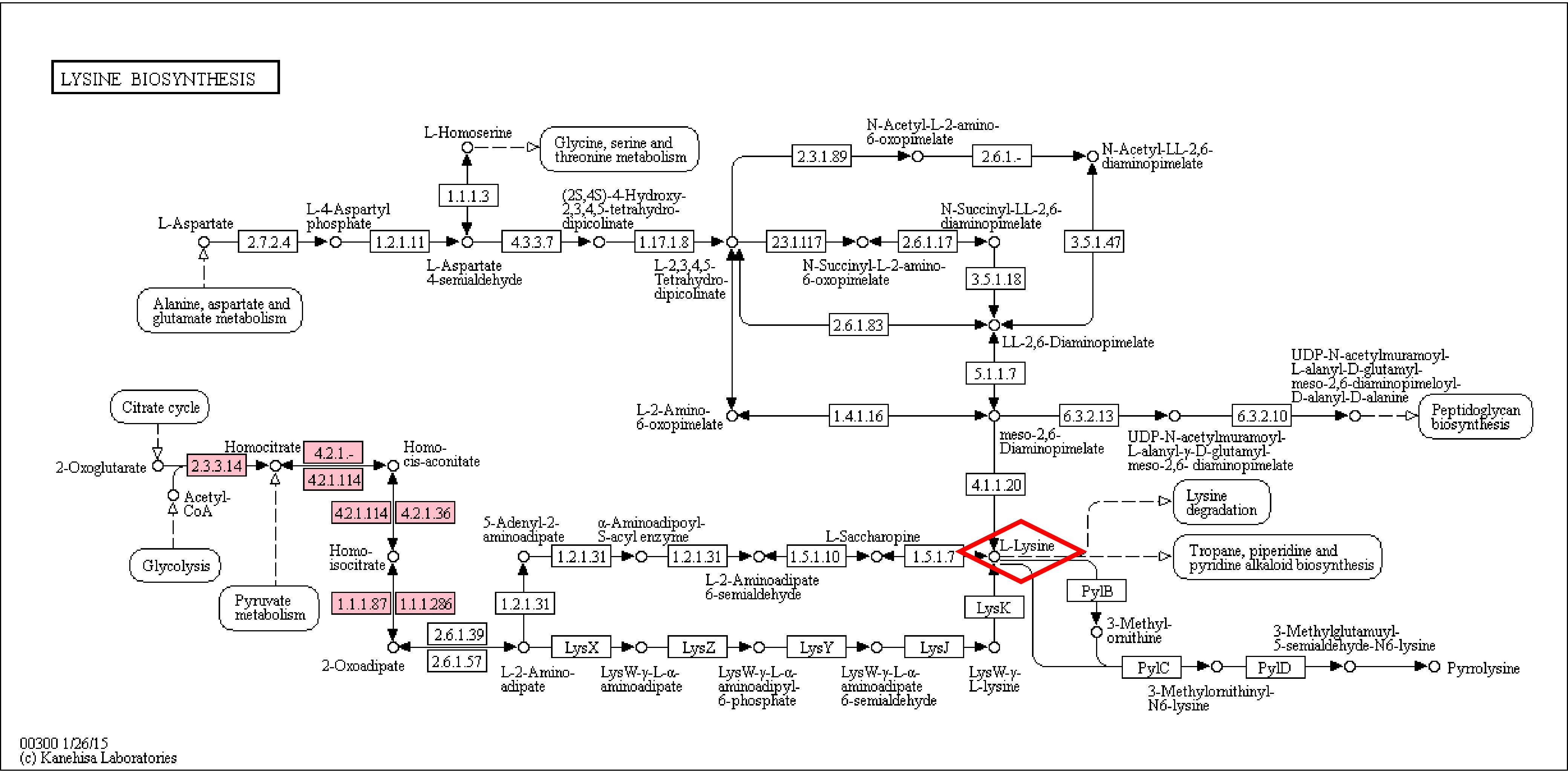

Lysine biosynthesis – 6 alternative modules defined by KEGG

Module F, second section

M00031: mediated by LysW, 2-aminoadipate => lysine

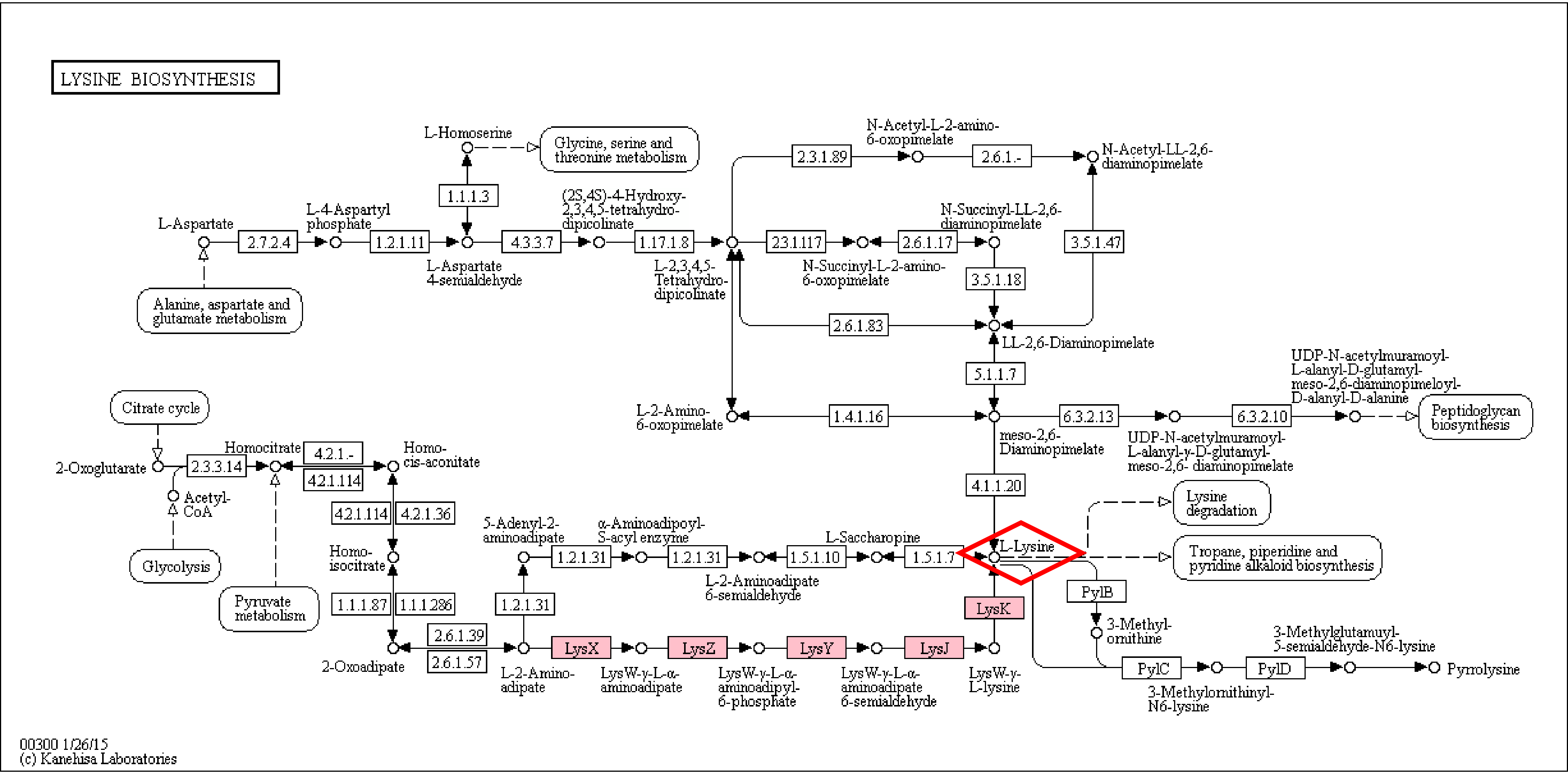

Arginine biosynthesis – KEGG module of ornithine, alternative A

M00028: glutamate => ornithine

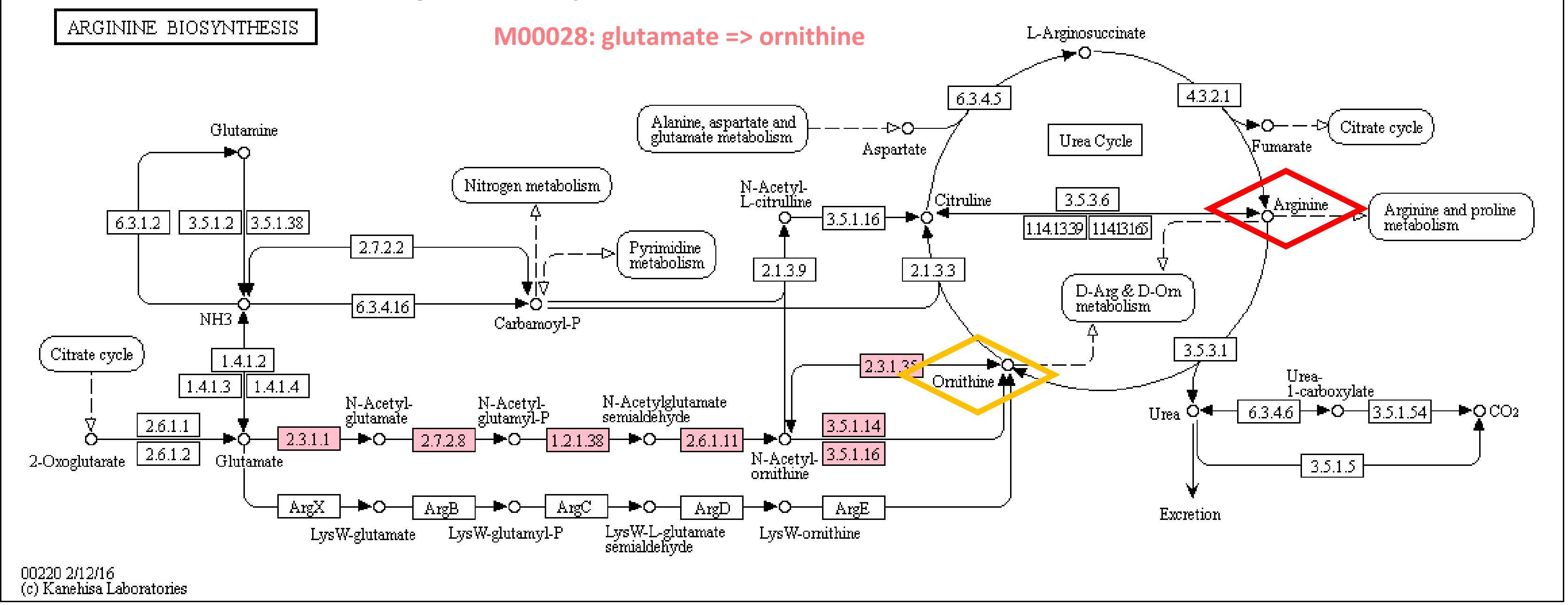

Arginine biosynthesis – KEGG module of ornithine, alternative B

M00763: mediated by LysW, glutamate => ornithine

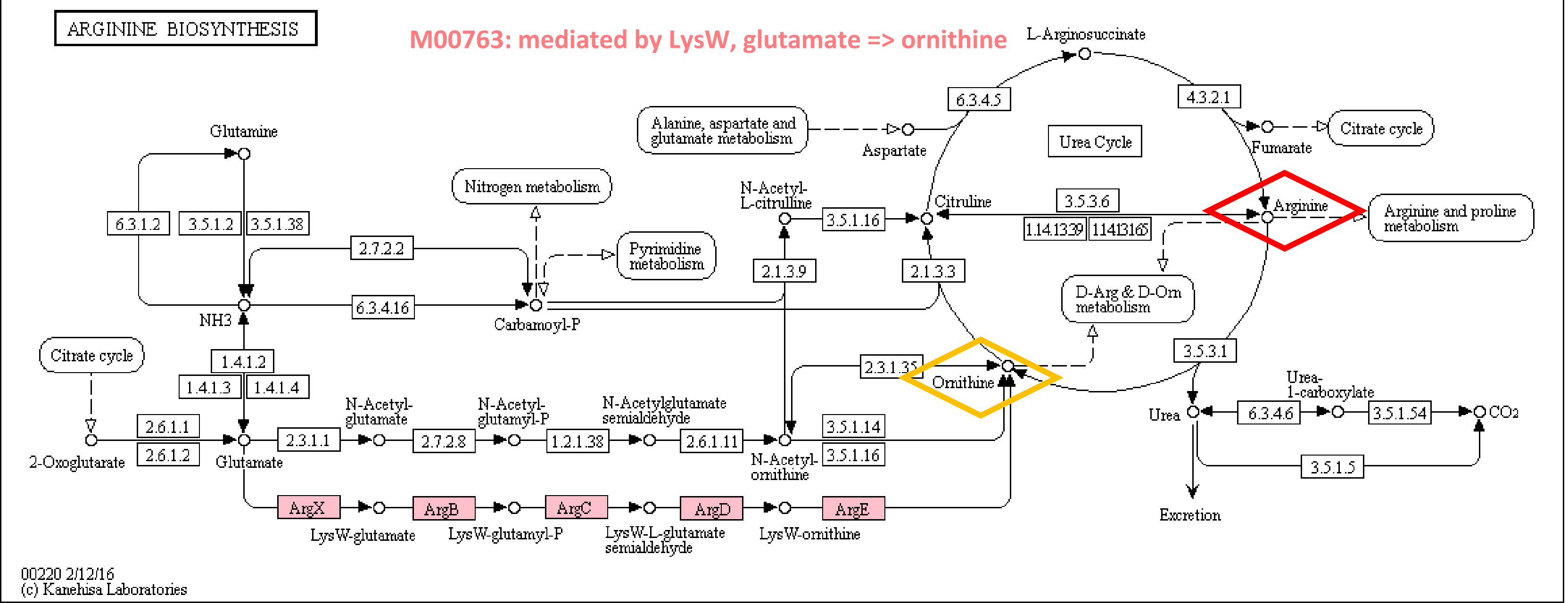

Arginine biosynthesis

Two alternative ways to produce ornithine

Arginine biosynthesis – KEGG module of urea cycle

M00029: Urea cycle

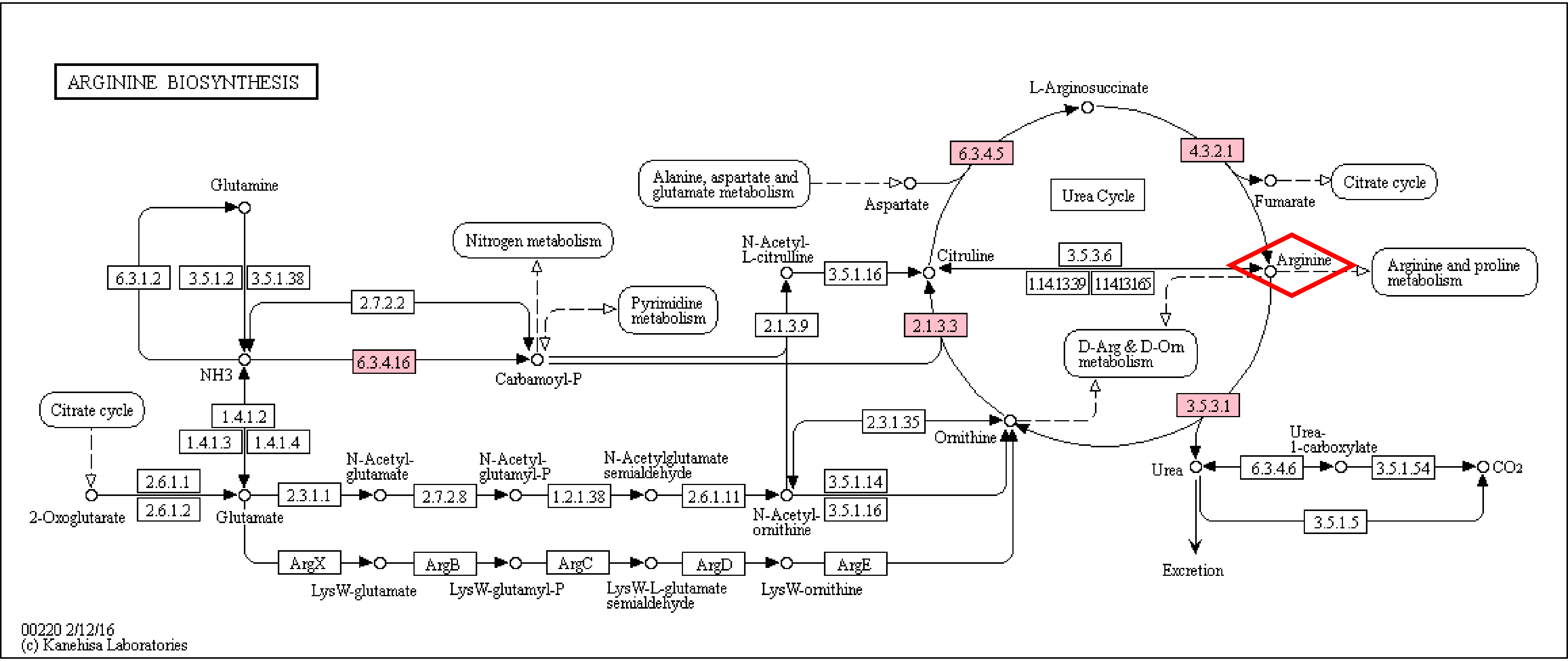

Proline biosynthesis – 1 KEGG module

M00015: glutamate => proline

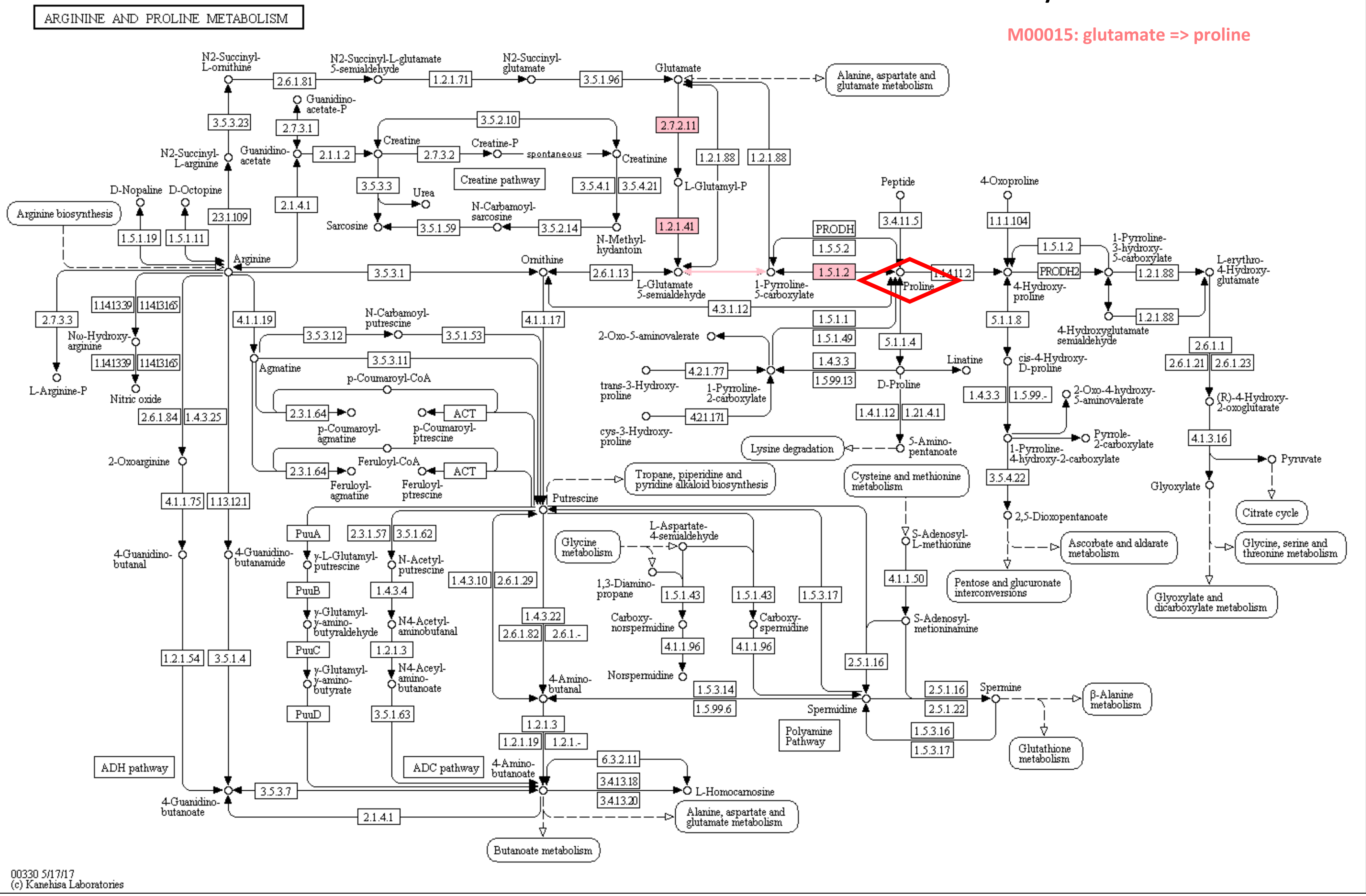

Histidine biosynthesis – 1 KEGG module

M00026: PRPP => histidine

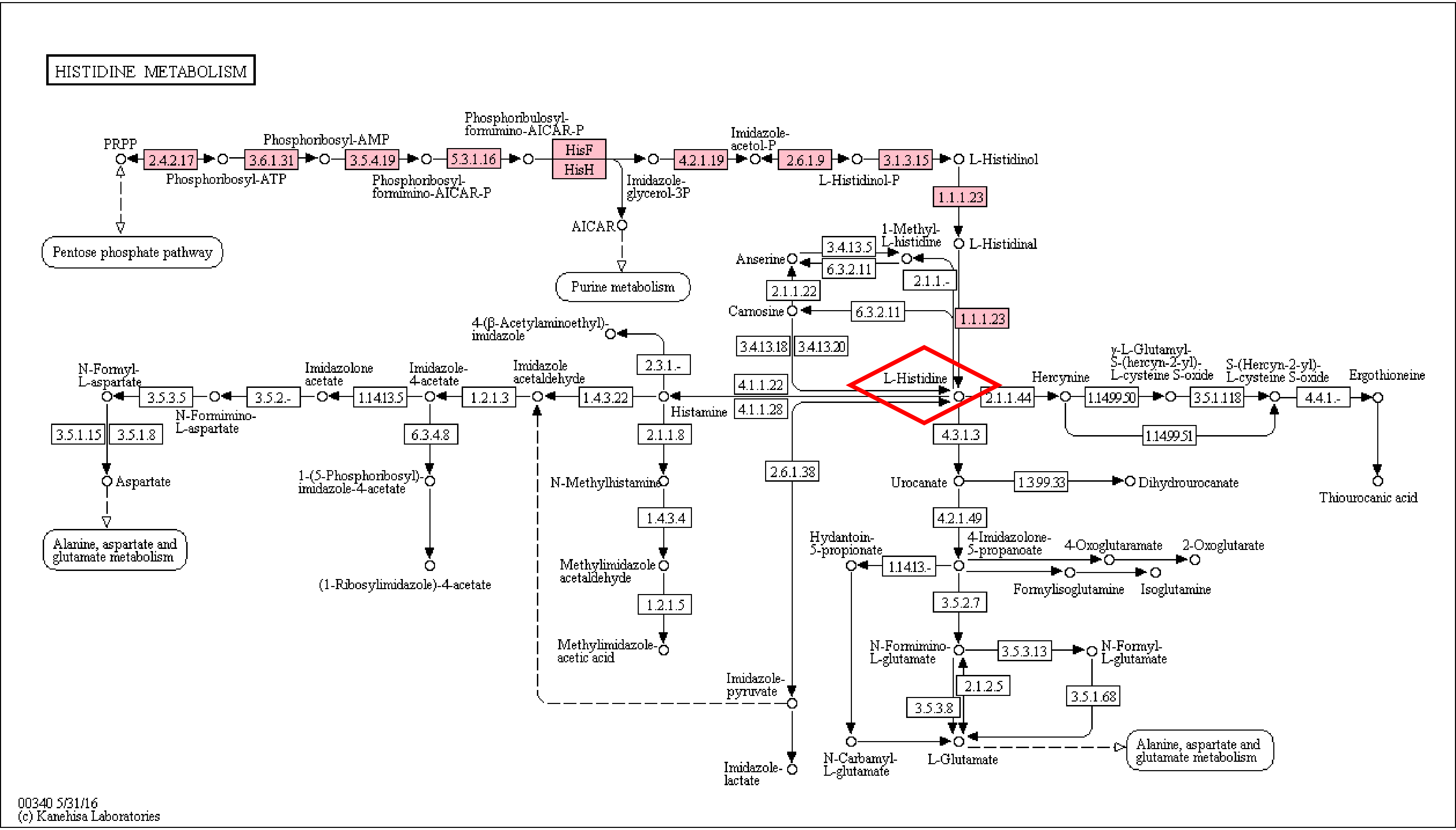

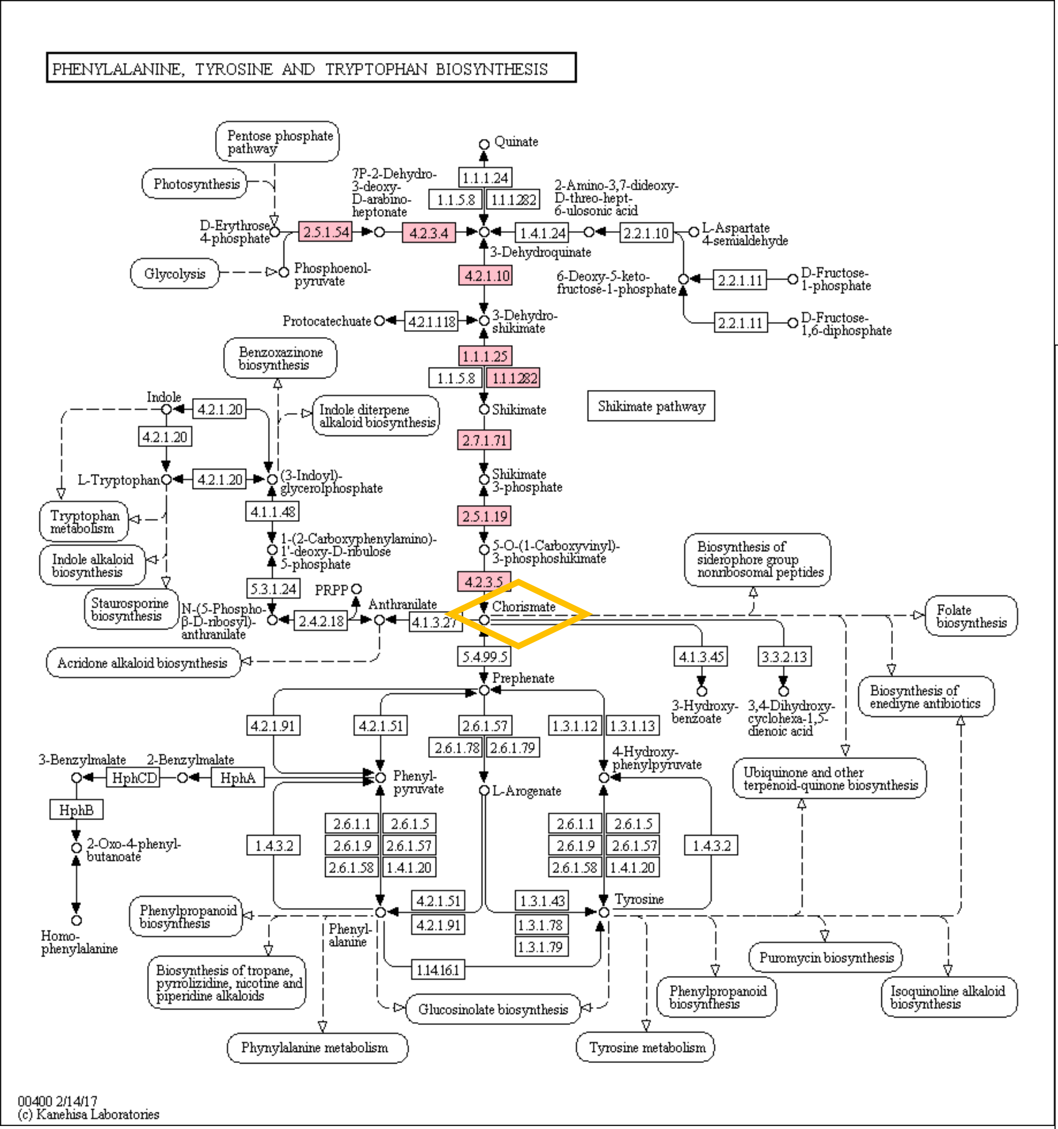

Phenylalanine biosynthesis – 2 KEGG modules are needed

Module 2: two alternative ways to produce phenylalanine

A M00024: chorismate => phenylalanine (via phenylpyruvate)

B chorismate => phenylalanine (via L-arogenate). This module is not defined by KEGG

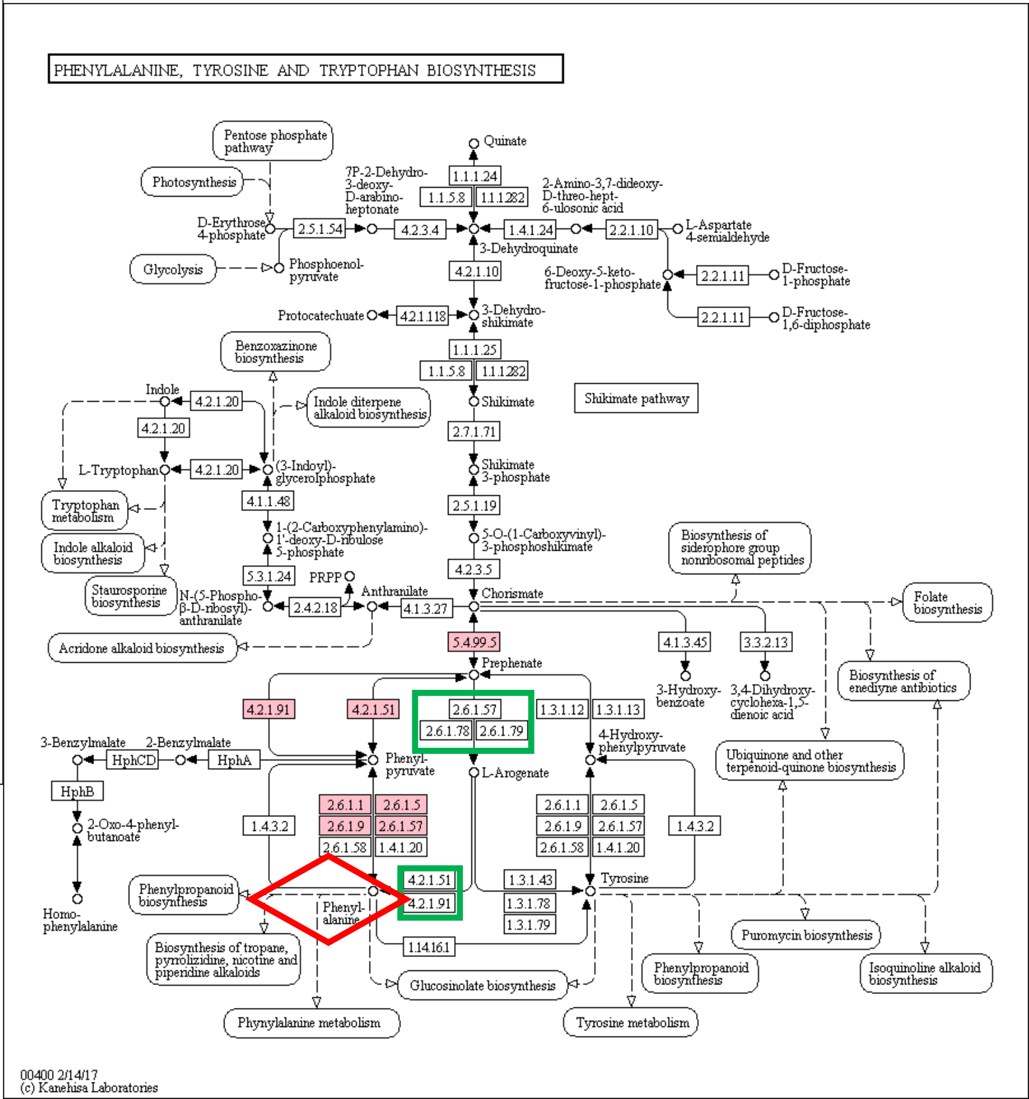

Module 1

M00022: phosphoenolpyruvate + erythrose-4P => chorismate

Tyrosine biosynthesis – 2 KEGG modules are needed

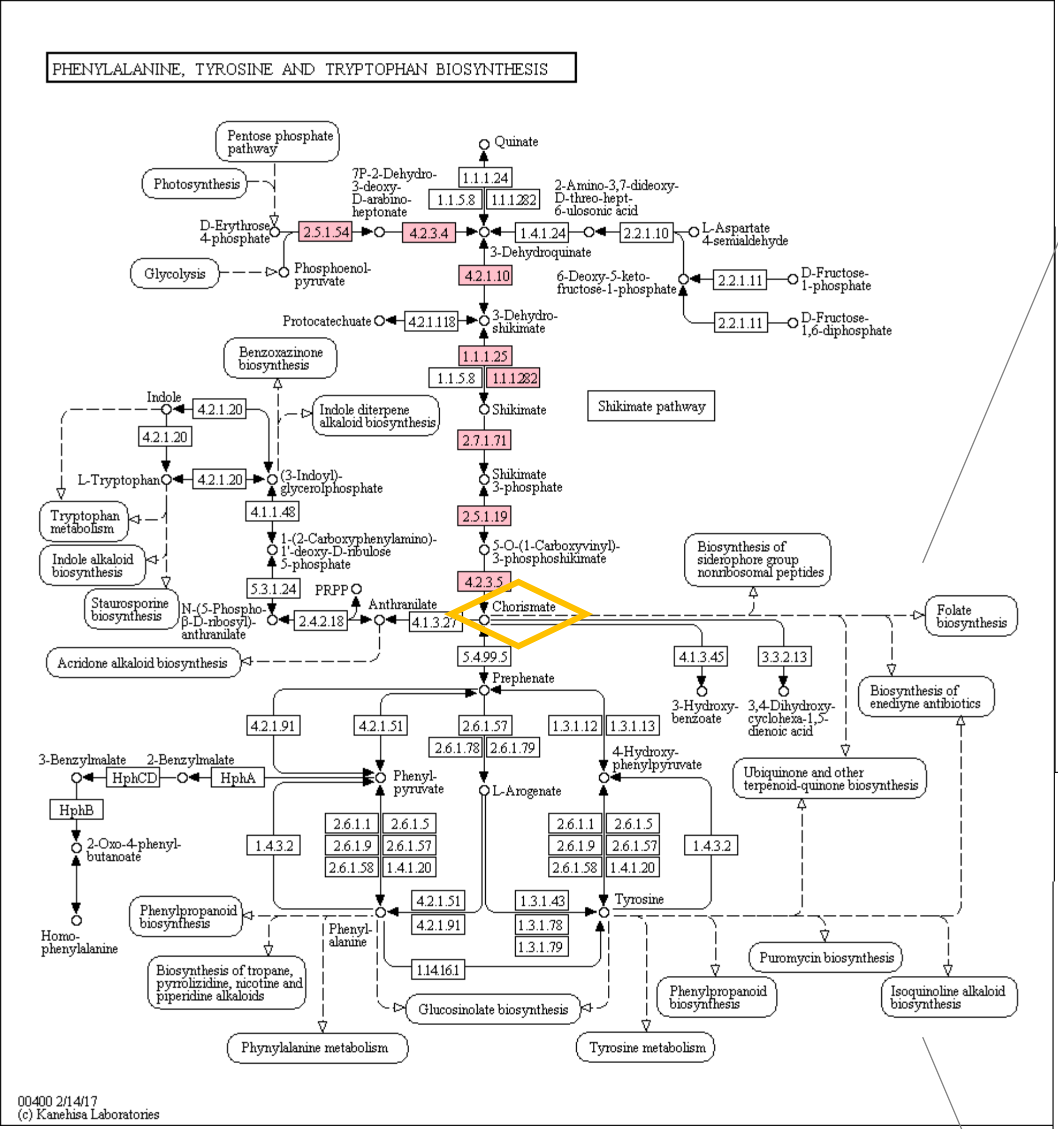

Module 1

M00022: phosphoenolpyruvate + erythrose-4P => chorismate

Module 2: two alternative ways to produce tyrosine

A M00025: chorismate => tyrosine

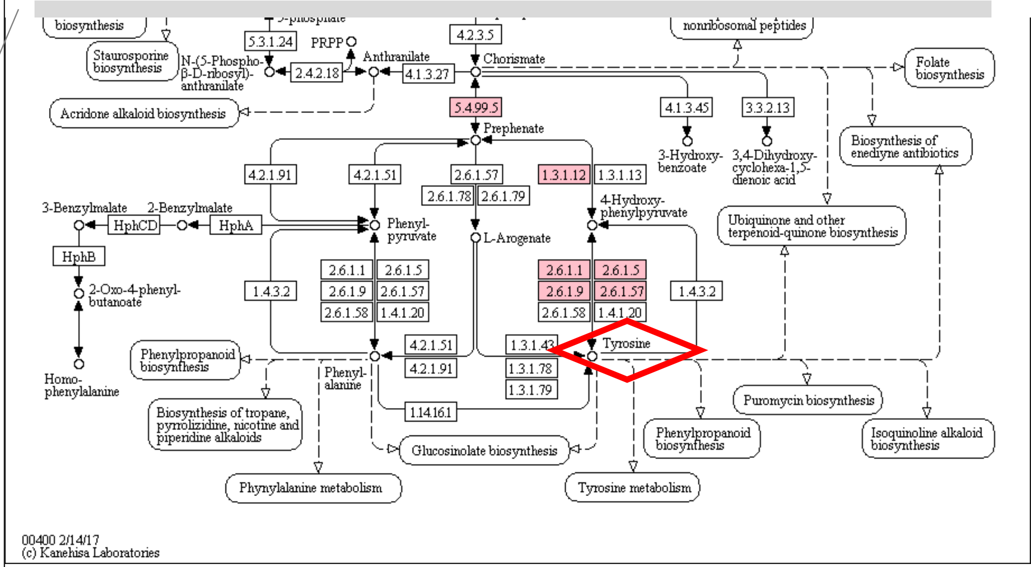

B M00040: prephenate => pretyrosine (L-arogenate)=> tyrosine

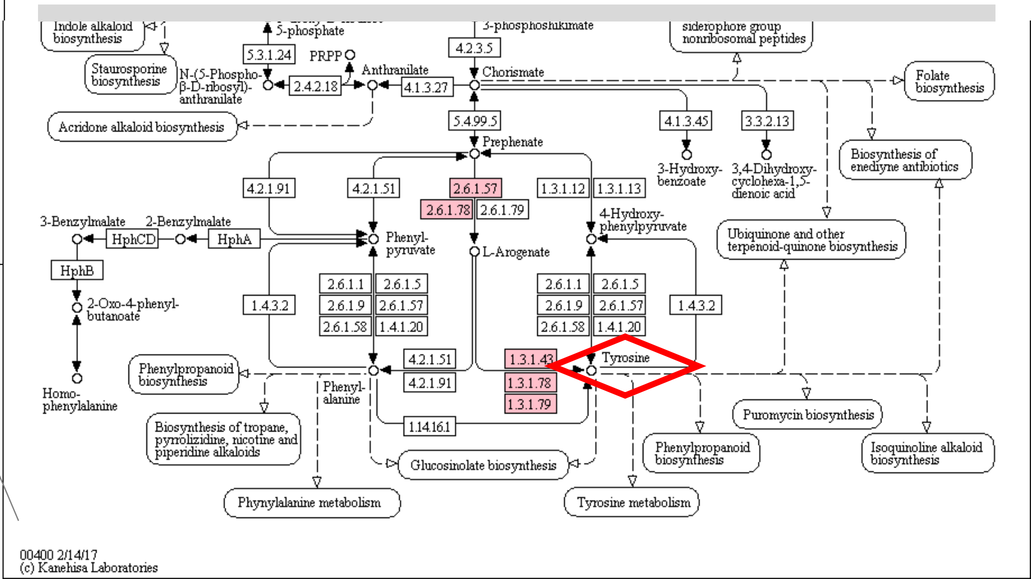

Tryptophan biosynthesis – 2 KEGG modules are needed

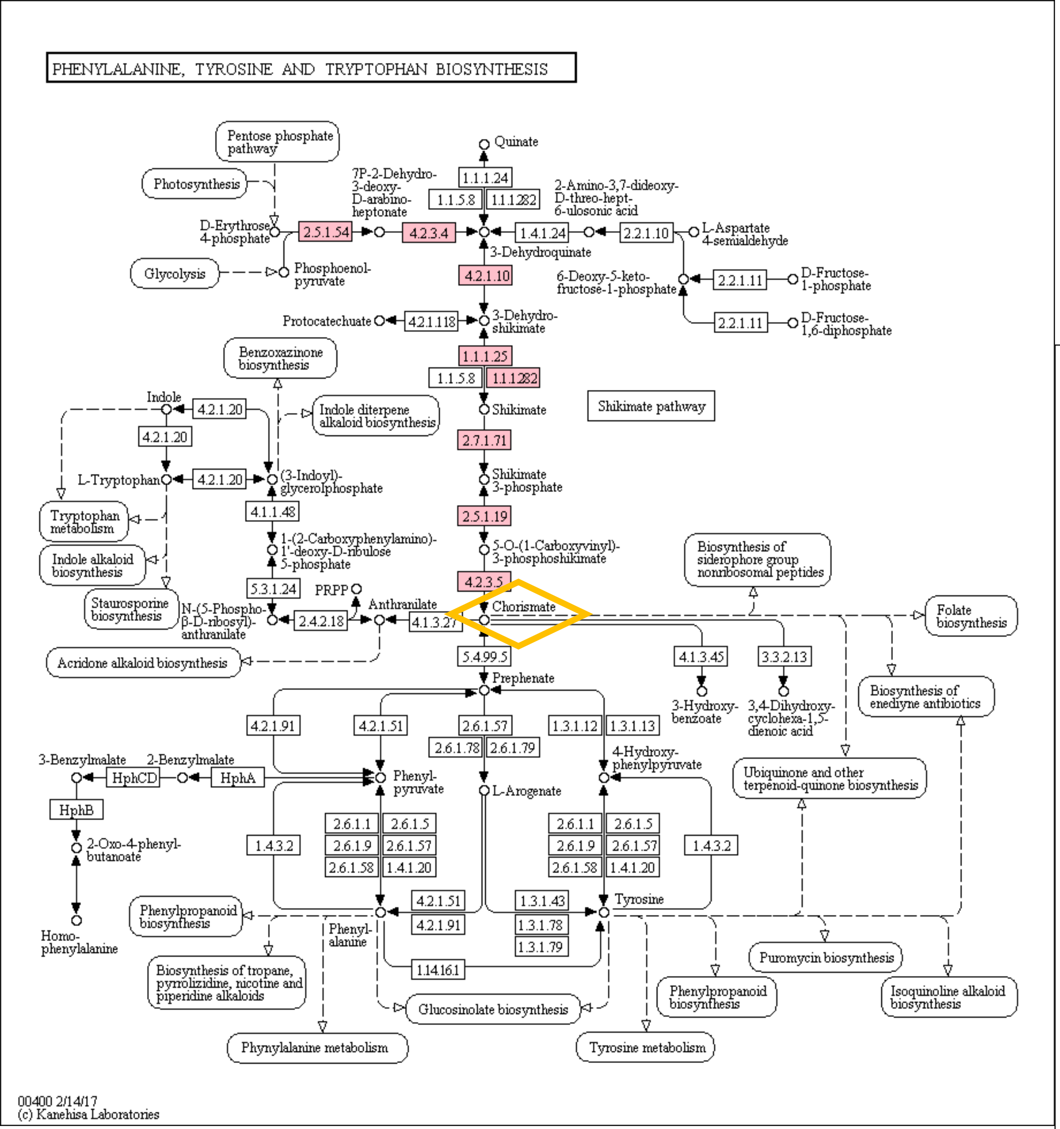

Module 2

M00023: chorismate => tryptophan

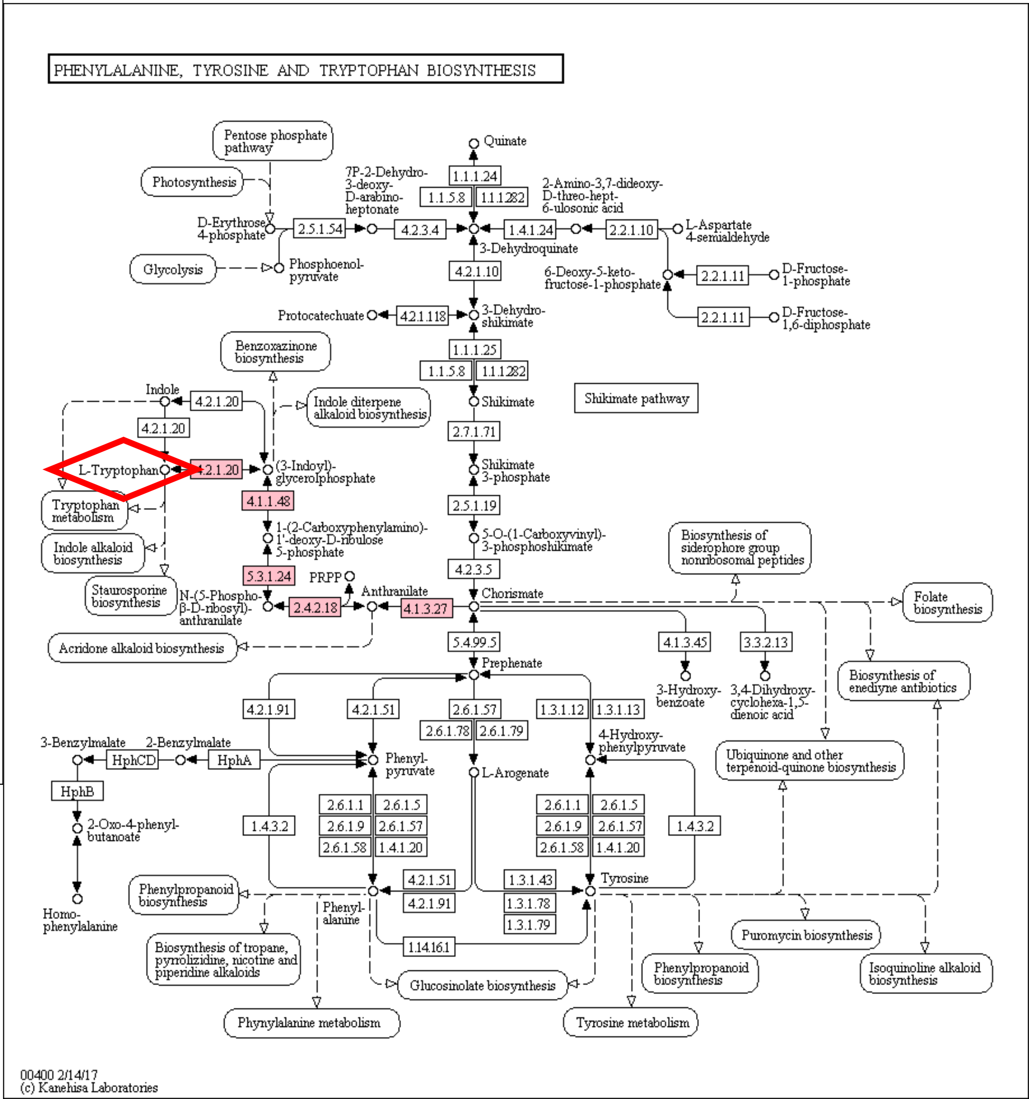

Module 1

M00022: phosphoenolpyruvate + erythrose-4P => chorismate
