# Supplementary material for: Vitamin Biosynthesis by Human Gut Butyrate-Producing Bacteria and Cross-Feeding in Synthetic Microbial Communities
Source: mBio. 2020 Jul 14;11(4):e00886-20. doi: 10.1128/mBio.00886-20 (PMC7360928; doi:10.1128/mBio.00886-20)
Supplement: FIG S3 [file mBio.00886-20-sf003.pdf]

a Number of replicates for vitamin auxotrophy screen to establish growth relative to positive control in the presence of all vitamins. Vitamin concentrations are given in Table S5 apart from strains A2-165, SL3/3, DSM 15176, L1-92, A1-86, M104/1 & A2-194, which were grown at 50X higher levels.

	A2-165	SL3/3	DSM 15176	DSM 3353	L1-92	SSC/2	GD/7	ART55/1	L2-50	A1-86	M104/1	M72/1	L1-82	M50/1	A2-194
**no biotin (B7)**	4	6	4	6	6	4	4	4	4	6	6	4	4	6	4
**no cobalamin (B12)**	4	6	4	6	4	4	4	4	3	4	6	4	4	6	4
**no folate (B9)**	4	6	4	10	6	4	4	4	8	6	6	4	4	6	4
**no folate (B9) or pABA**	4	5	4	12	4	4	4	4	11	4	5	4	4	6	4
**no niacin (B3)**	4	6	4	6	4	4	4	4	4	4	6	4	4	6	4
**no pantothenate (B5)**	4	6	4	6	4	4	4	4	4	4	6	4	4	6	4
**no pyridoxine (B6)**	4	6	3	6	4	4	4	4	4	4	6	4	4	6	4
**no riboflavin (B2)**	4	6	3	5	4	4	4	4	4	4	6	4	4	6	4
**no thiamine (B1)**	4	6	4	6	6	4	4	4	4	4	6	4	3	6	4
**no vitamins**	4	6	10	10	6	6	4	4	10	6	6	4	4	6	4

b Growth curves for vitamin auxotrophy screen at a single vitamin concentration. Gray lines indicate data used for Fig. 3 (thin line: Growth in Hungate tubes, single OD reading; thick line: growth in 96-well plate, average data over 1 h).

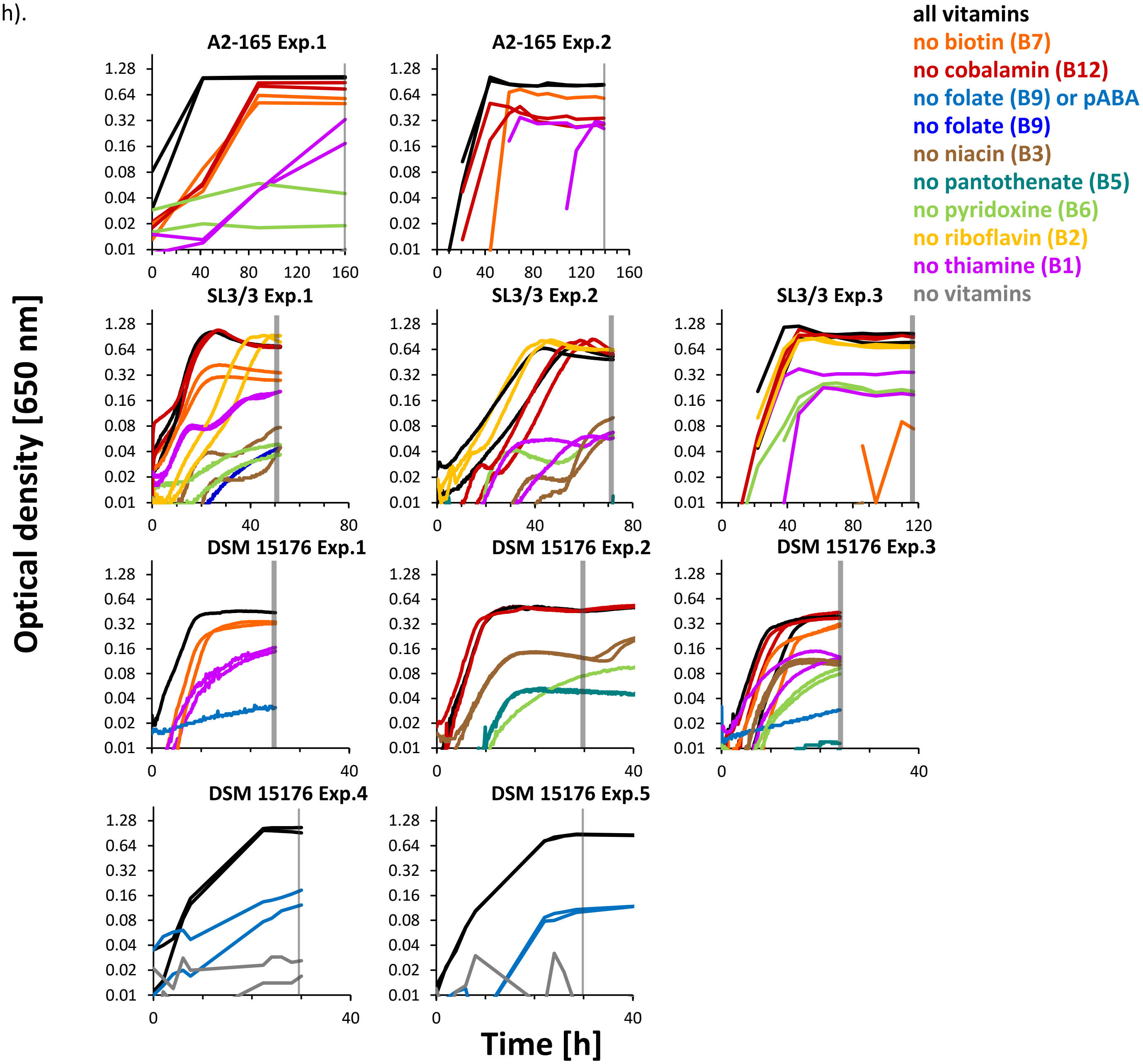

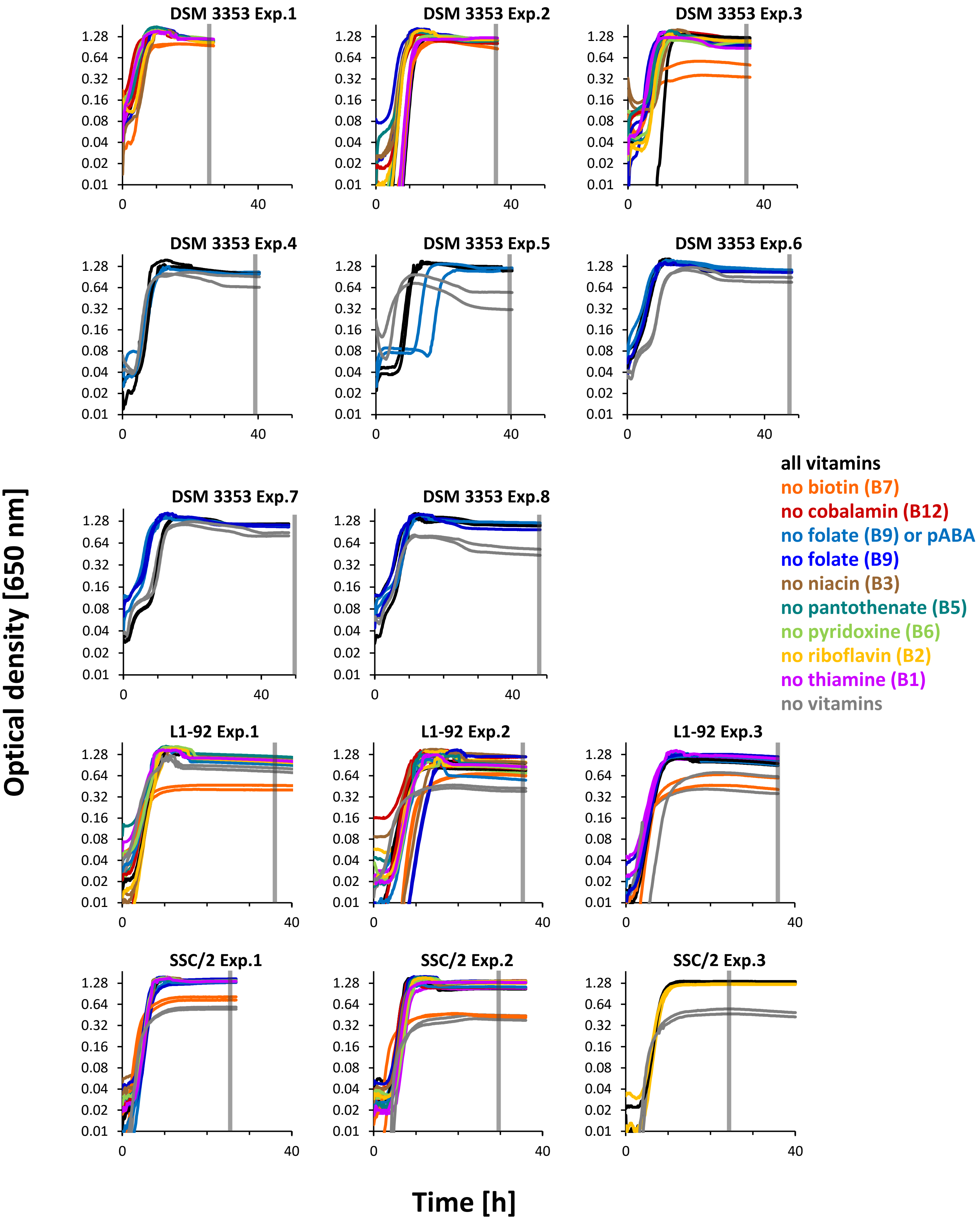
DSM 3353 Exp.1
DSM 3353 Exp.2
DSM 3353 Exp.3
DSM 3353 Exp.4
DSM 3353 Exp.5
DSM 3353 Exp.6
DSM 3353 Exp.7
DSM 3353 Exp.8
L1-92 Exp.1
L1-92 Exp.2
L1-92 Exp.3
SSC/2 Exp.1
SSC/2 Exp.2
SSC/2 Exp.3
1.28
0.64
0.32
0.16
0.08
0.04
0.02
0.01
0
40
all vitamins
no biotin (B7)
no cobalamin (B12)
no folate (B9) or pABA
no folate (B9)
no niacin (B3)
no pantothenate (B5)
no pyridoxine (B6)
no riboflavin (B2)
no thiamine (B1)
no vitamins
Optical density [650 nm]
Time [h]

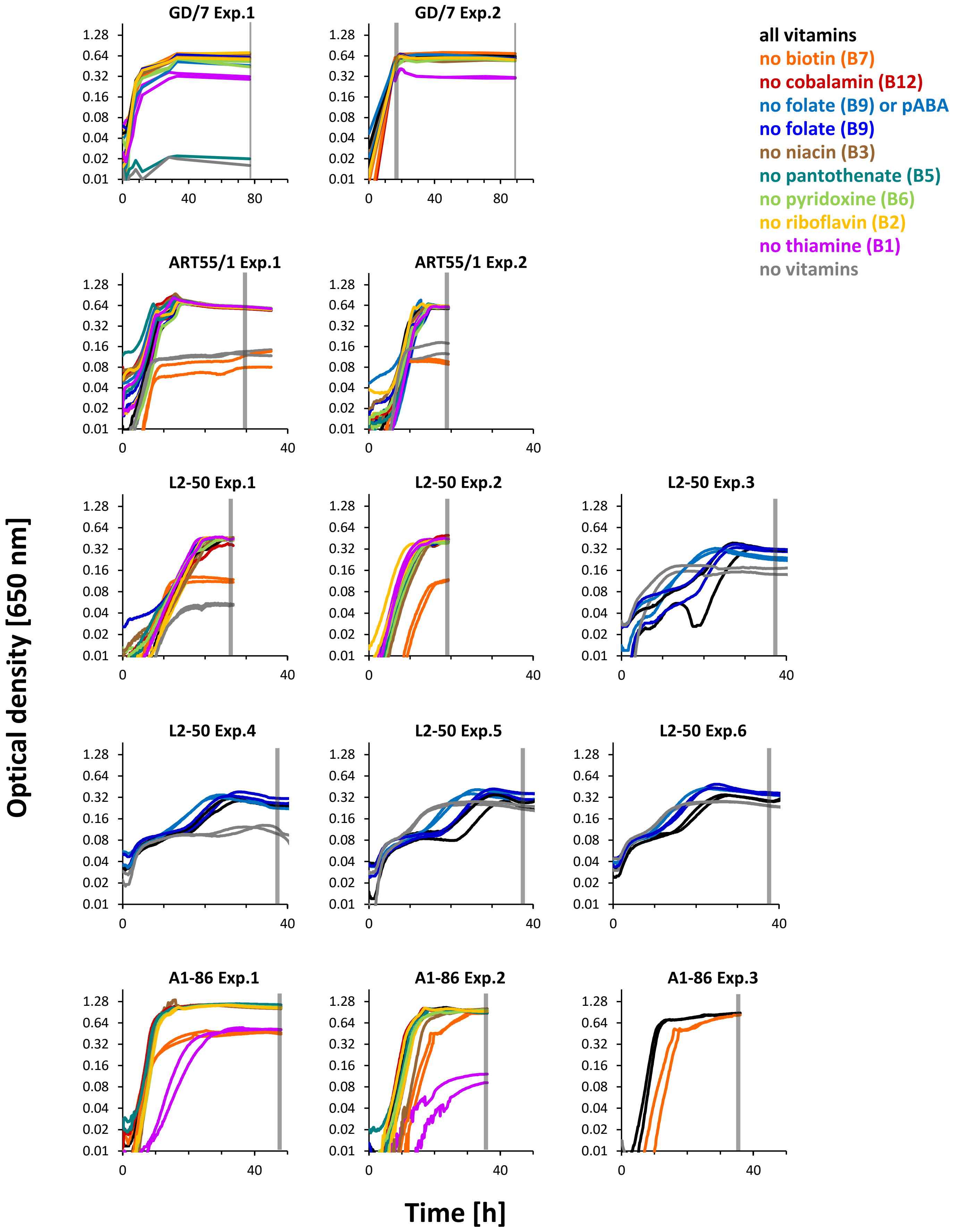
GD/7 Exp.1
GD/7 Exp.2
ART55/1 Exp.1
ART55/1 Exp.2
L2-50 Exp.1
L2-50 Exp.2
L2-50 Exp.3
L2-50 Exp.4
L2-50 Exp.5
L2-50 Exp.6
A1-86 Exp.1
A1-86 Exp.2
A1-86 Exp.3
1.28
0.64
0.32
0.16
0.08
0.04
0.02
0.01
0
40
80
Optical density [650 nm]
Time [h]
all vitamins
no biotin (B7)
no cobalamin (B12)
no folate (B9) or pABA
no folate (B9)
no niacin (B3)
no pantothenate (B5)
no pyridoxine (B6)
no riboflavin (B2)
no thiamine (B1)
no vitamins

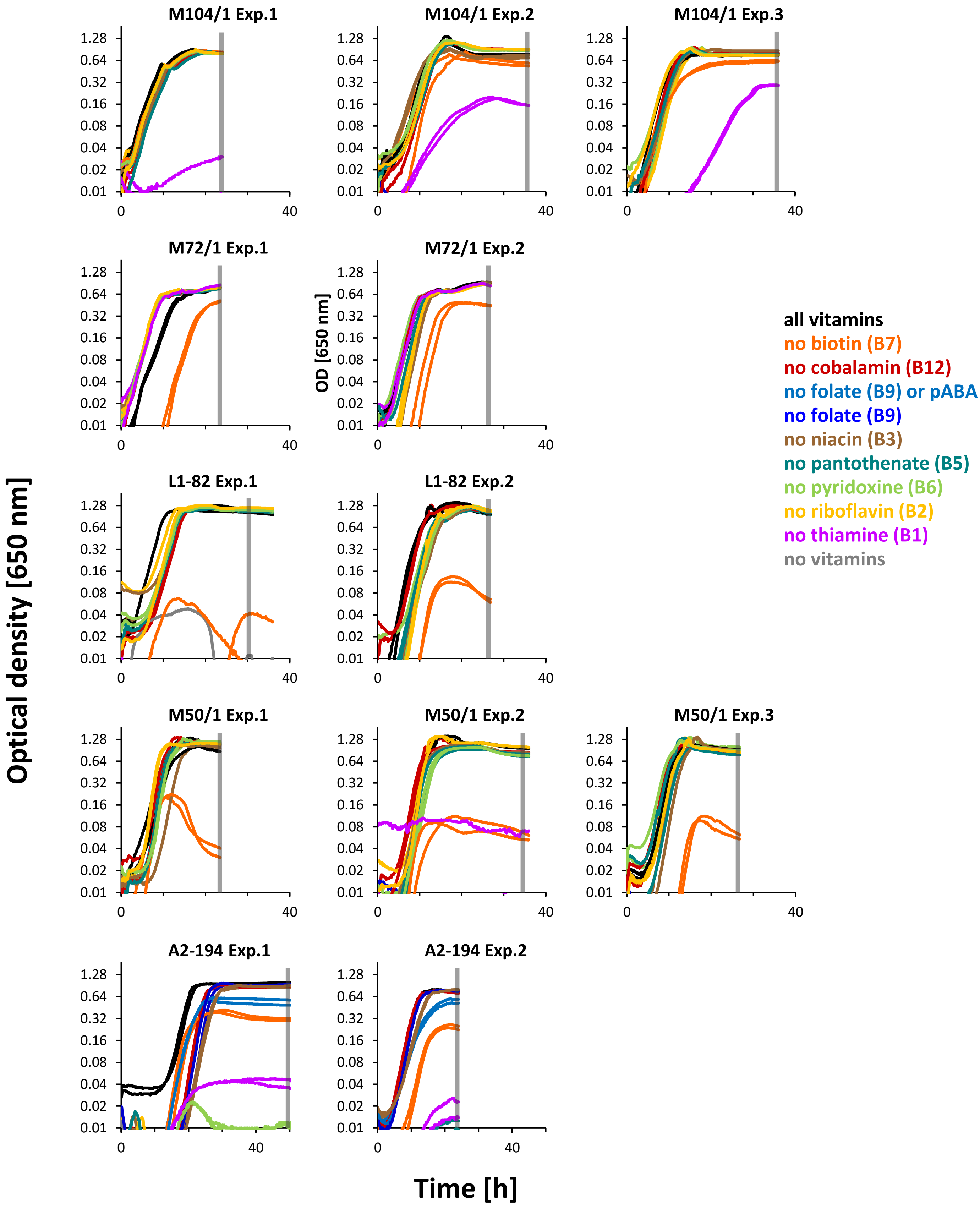
M104/1 Exp.1
M104/1 Exp.2
M104/1 Exp.3
M72/1 Exp.1
M72/1 Exp.2
L1-82 Exp.1
L1-82 Exp.2
M50/1 Exp.1
M50/1 Exp.2
M50/1 Exp.3
A2-194 Exp.1
A2-194 Exp.2
1.28
0.64
0.32
0.16
0.08
0.04
0.02
0.01
0
40
OD [650 nm]
Optical density [650 nm]
Time [h]
all vitamins
no biotin (B7)
no cobalamin (B12)
no folate (B9) or pABA
no folate (B9)
no niacin (B3)
no pantothenate (B5)
no pyridoxine (B6)
no riboflavin (B2)
no thiamine (B1)
no vitamins

c Number of replicates for pure culture growth (stationary OD/growth rate) in the presence of increasing concentrations of folate or thiamine.

	thiamine							folate						
	0	0.05 ng	0.5 ng	5 ng	0.05 µg	0.5 µg	5 µg	0	0.05 ng	0.5 ng	5 ng	0.05 µg	0.5 µg	5 µg
S. variabile **DSM 15176**	6/3	6/3	6/3	6/3	6/3	6/3	6/3	6/6	6/6	6/6	6/6	6/6	6/6	6/6
E. rectale **A1-86**	6/6	6/3	6/3	6/3	5/2	6/3	6/3	6/6	6/6	6/6	6/6	4/4	5/5	6/6
R. intestinalis **M50/1**	6/6	6/6	6/6	6/6	6/6	6/6	6/6	6/5	6/5	6/5	6/6	6/6	6/6	6/6
R. inulinivorans **A2-194**	6/6	6/6	6/6	6/6	6/6	6/6	6/6							
R. faecis **M72/1**	6/6	6/5	6/5	6/6	6/6	6/6	6/6	5/6	6/6	5/6	6/6	5/5	6/6	6/5
L. paracasei	4/3	5/5	6/6	6/6	6/6	6/6	6/6							
Coprococcus **sp. ART55/1**								6/6	6/6	6/6	6/6	6/6	6/6	6/6
S. thermophilus								6/3	6/6	6/6	6/6	6/6	6/6	6/6
B. bifidum								6/6	6/6	6/6	6/6	6/6	6/6	6/6

d Growth curves for all vitamin conditions and strains tested. Gray lines indicate data used for Fig. 4c and d. For colour coding see Fig. 4.

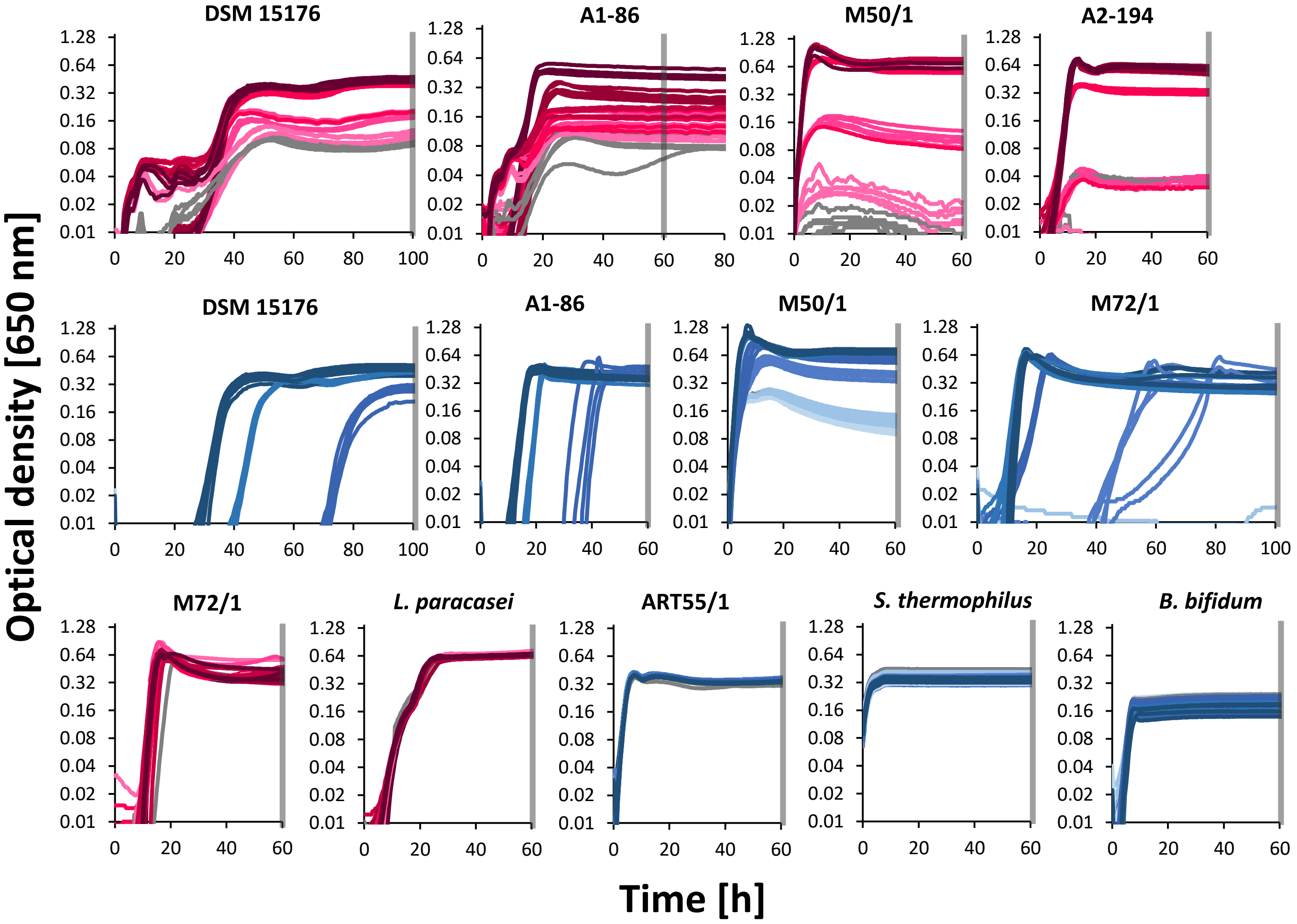

Fig. S3 Growth data of vitamin experiments in pure and mixed culture. (a) Number of replicates for vitamin auxotrophy screen to establish growth relative to positive control in the presence of all vitamins. Vitamin concentrations are given in Table S5 apart from strains A2-165, SL3/3, DSM 15176, L1-92, A1-86, M104/1 & A2-194, which were grown at 50X higher levels. (b) Growth curves for vitamin auxotrophy screen at a single vitamin concentration. Gray lines indicate data used for Fig. 3 (thin line: Growth in Hungate tubes, single OD reading; thick line: growth in 96-well plate, average data over 1 h). (c) Number of replicates for pure culture growth (stationary OD/growth rate) in the presence of increasing concentrations of folate or thiamine. (d) Growth curves for all vitamin conditions and strains tested. Gray lines indicate data used for Fig. 4c and d. For colour coding see Fig. 4.
