# Supplementary material for: Vitamin Biosynthesis by Human Gut Butyrate-Producing Bacteria and Cross-Feeding in Synthetic Microbial Communities
Source: mBio. 2020 Jul 14;11(4):e00886-20. doi: 10.1128/mBio.00886-20 (PMC7360928; doi:10.1128/mBio.00886-20)
Supplement: FIG S4 [file mBio.00886-20-sf004.pdf]

a Overview of strains and amino acids tested in comparison to the positive control in the presence of all amino acids. The growth of strains tested in the absence of specific amino acids are highlighted. Numbers in gray refer to in silico data as per Table S2.

- Stationary OD significantly lower than positive control but overall growth very similar (see growth curves)
- Stationary OD not significantly different to positive control
- Stationary OD significantly higher than positive control but overall growth very similar (see growth curves)
- Stationary OD significantly lower than positive control
- Stationary OD significantly lower than positive control and growth rate reduced

Amino acid absent	Ala	Asp	Asn	Glu	Gln	Gly	Ser	Thr	Cys	Met	Val	Leu	Ile	Lys	Arg	Pro	His	Phe Tyr Trp*	Phe	Tyr	Trp	all 19$
TOTAL NUMBER OF GENES	1	1	1	1	1	1	1	5	2	7	5	5	6	7	8	3	10	7	+3	+3	+7	
F. prausnitzii A2-165	0?	0	0	0	0	0	0	0	0	0?	0	0	0	0	0	0	0	0	0	0	7	
F. prausnitzii SL3/3	0?	0	0	0	0	0	0	0	0	0?	0	0	0	0	0	0	0	0	0	0	7	
S. variabile DSM 15176	0?	0	0	0	0	0	0	0	0	0?	0	0	0	0	0	0	0	1	0	0	6	
A. hallii DSM 3353	0	0	1	0	0	0	0	0	0	0?	0	0	0	0	0	0	0	0	0	0	0	
A. caccae L1-92	0	0	0	0	0	0	0	1	0	0?	0	0	0	0	0	0	0	0	0	0	0	
A. hadrus SSC/2	0	0	0	0	0	0	0	1	0	0	0	0	0	0	0	0	0	0	0	0	0	
C. catus GD/7	0?	0	0	0	0	0	0	0	0	0	0	0	0	0	0	0	0	0	0	0	4	
Clostridium sp. L2-50	0	0	0	0	0	0	0	0	0	0	0	0	0	0	0	0	0	0	0	0	0	
Coprococcus sp. ART55/1	0	0	0	0	0	0	0	0	0	0	0	0	0	1	0	0	1	3	0	1	0	
E. rectale A1-86	0	0	0	0	0	0	0	0	0	0	0	0	0	0	0	0	0	0	0	0	0	
E. rectale M104/1	0	0	0	0	0	0	0	0	0	0	0	0	0	0	0	0	0	0	0	0	0	
R. faecis M72/1	0?	0	0	0	0	0	0	0	0	0	0	0	0	0	0	0	0	0	0	0	0	
R. intestinalis L1-82	0?	0	0	0	0	0	0	0	0	0	0	0	0	0	0	0	0	0	0	0	0	
R. intestnalis M50/1	0?	0	0	0	0	0	0	0	0	0	0	0	0	0	0	0	0	0	0	0	0	
R. inulinivorans A2-194	0?	0	0	0	0	0	0	0	0	0	0	0	0	0	0	0	0	0	0	0	7	

*Total number of genes relate to chorismate biosynthesis, shared by all aromatic amino acids

$Cysteine was present

b Growth curves (left) and stationary OD relative to positive control (right) for all amino acid conditions and strains tested. Gray lines indicate data used for stationary OD data. The pooled standard deviations ranged between 3.9 and 30.6 per strain. Letters refer to the Tukey test results. Treatments with different a letters in common are not significantly different (P-value < 0.05).

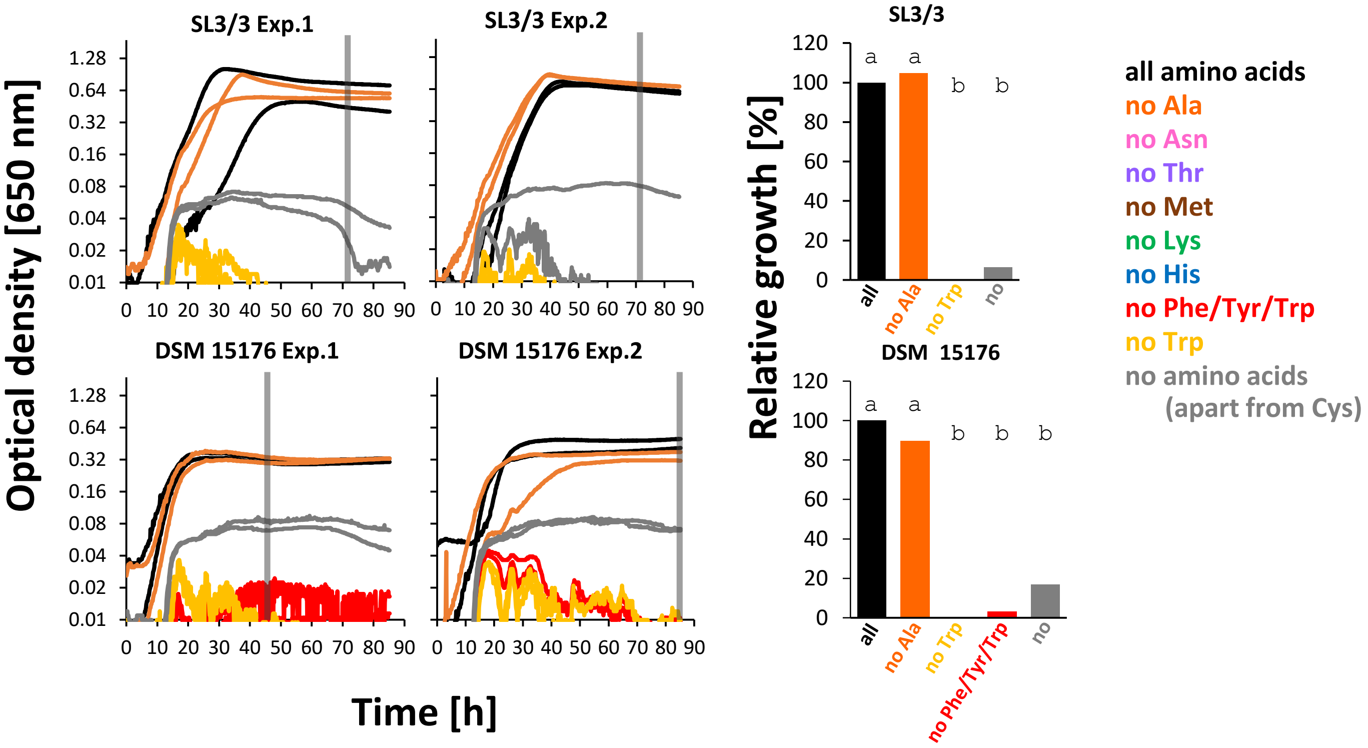

Fig. S4 2/3

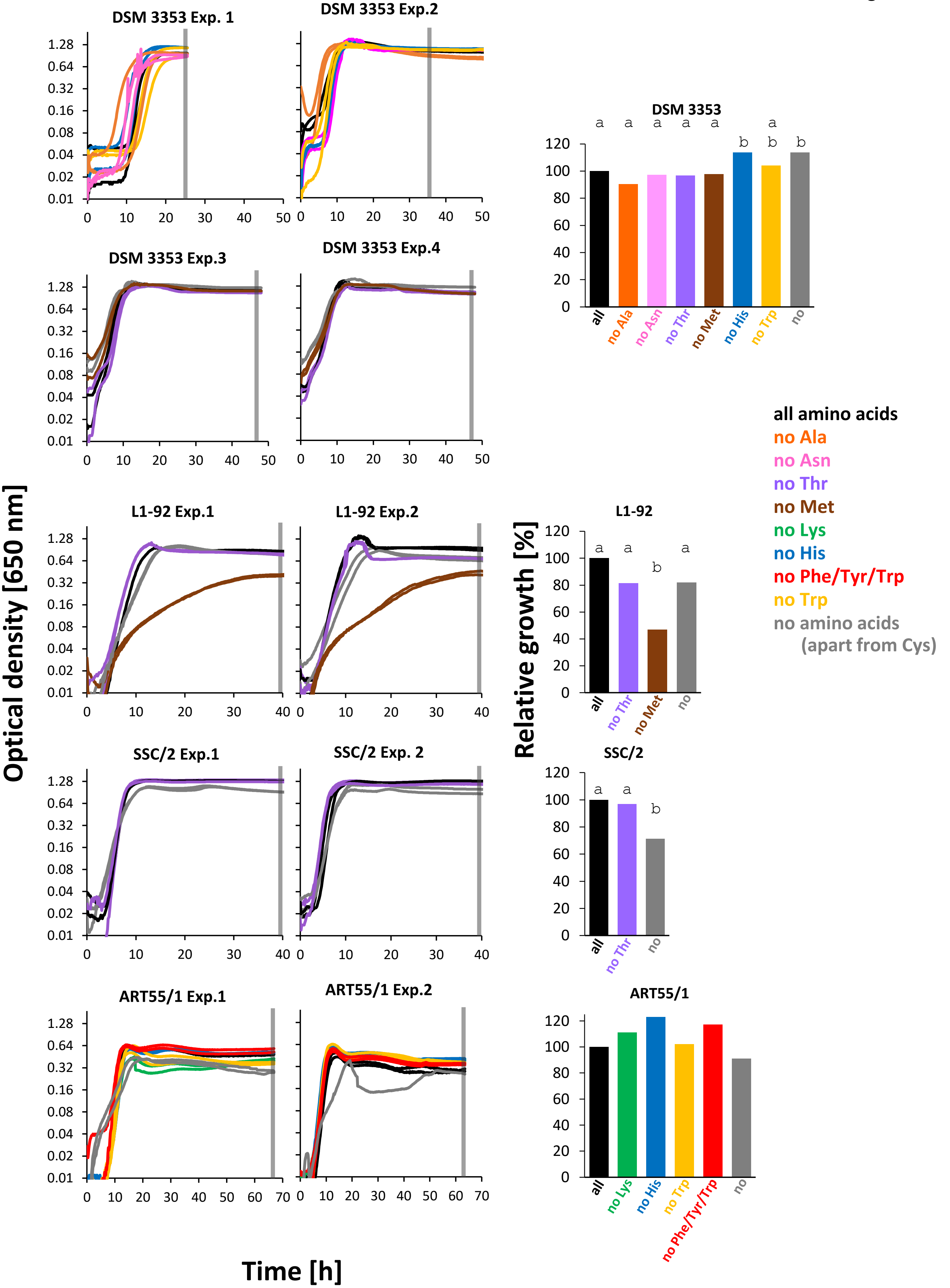
DSM 3353 Exp. 1
DSM 3353 Exp.2
DSM 3353 Exp.3
DSM 3353 Exp.4
L1-92 Exp.1
L1-92 Exp.2
SSC/2 Exp.1
SSC/2 Exp. 2
ART55/1 Exp.1
ART55/1 Exp.2
Optical density [650 nm]
Time [h]
DSM 3353
L1-92
SSC/2
ART55/1
Relative growth [%]
all
no Ala
no Asn
no Thr
no Met
no His
no Trp
no
no Lys
no Phe/Tyr/Trp
all amino acids
no Ala
no Asn
no Thr
no Met
no Lys
no His
no Phe/Tyr/Trp
no Trp
no amino acids
(apart from Cys)

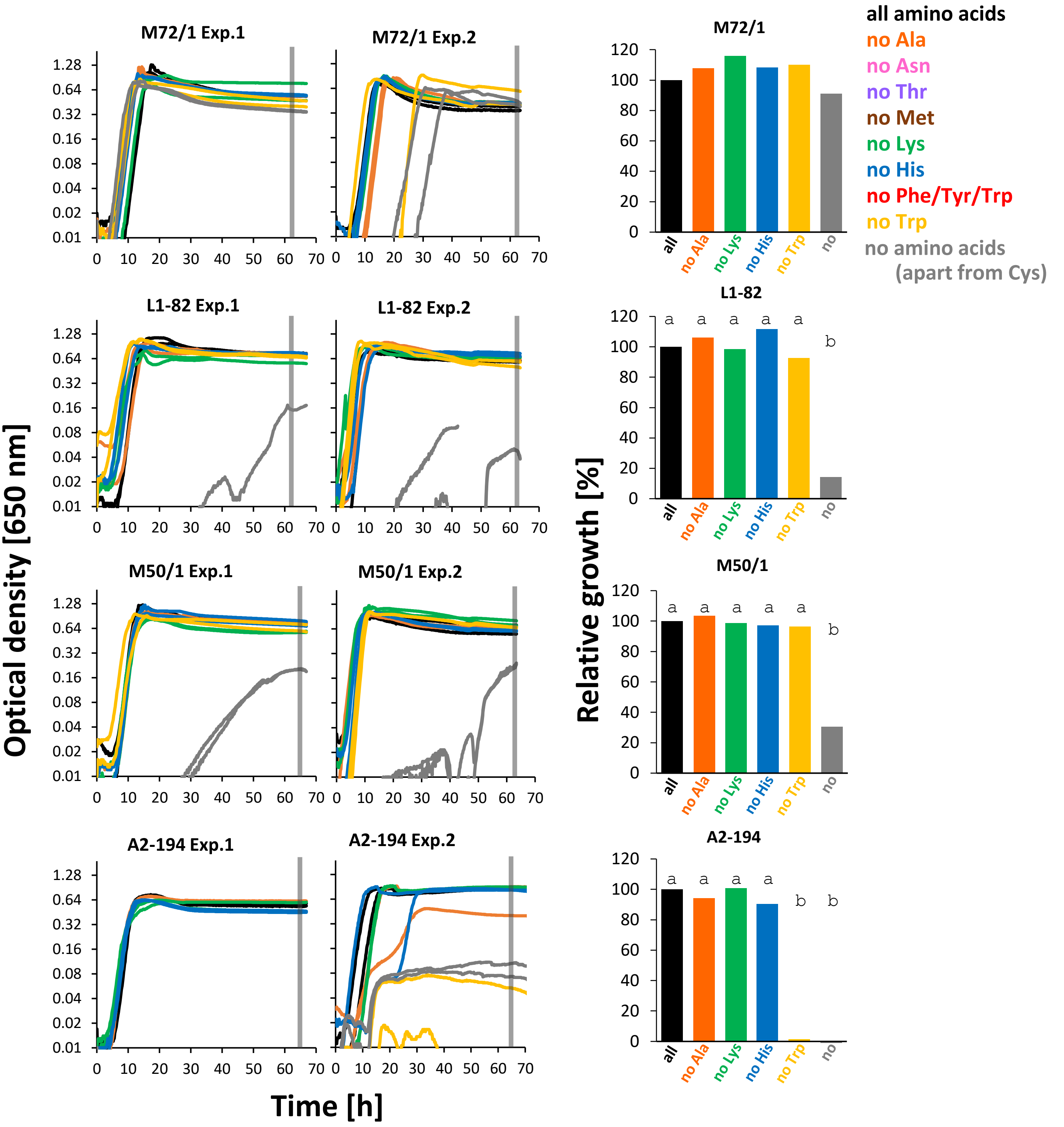

Fig. S4 Relative growth of several butyrate producing strains in the 3rd passage of culture in AA-CDM lacking certain amino acids compared to the positive control with all amino acids present. (a) Overview of strains and amino acids tested in comparison to the positive control in the presence of all amino acids. The growth of strains tested in the absence of specific amino acids are highlighted. Numbers in gray refer to *in silico* data as per Table S2. (b) Growth curves (left) and stationary OD relative to positive control (right) for all amino acid conditions and strains tested. Gray lines indicate data used for stationary OD data. The pooled standard deviations ranged between 3.9 and 30.6 per strain. Letters refer to the Tukey test results. Treatments with different a letters in common are not significantly different (P-value < 0.05).
