# Supplementary material for: Vitamin Biosynthesis by Human Gut Butyrate-Producing Bacteria and Cross-Feeding in Synthetic Microbial Communities
Source: mBio. 2020 Jul 14;11(4):e00886-20. doi: 10.1128/mBio.00886-20 (PMC7360928; doi:10.1128/mBio.00886-20)
Supplement: TABLE S3 [file mBio.00886-20-st003.pdf]

Table S3 Bacterial strains used in the study and genome information (from https://www.ncbi.nlm.nih.gov/genome/microbes/).

**Organism**[1]	**Reference**	**Genome size [Mb]**	**Assembly level**	**Number of scaffolds or contigs**	**Contig N50**	**Contig L50**	**Total assembly gap length [kb]**[2]
Faecalibacterium prausnitzii A2-165	1	3.09035	Scaffold	20	63,061	14	9.5
Faecalibacterium prausnitzii SL3/3	2	3.21442	Chromosome	1	67,073	14	48.5
Subdoligranulum variabile DSM 15176	3	3.24547	Scaffold	11	166,480	6	8.0
Anaerobutyricum hallii DSM 3353	4, 5	3.29	Contig	175	33,110	28	0
Anaerostipes caccae DSM 14662 (L1-92)	1, 6	3.60694	Scaffold	26	261,254	4	1.3
Anaerostipes hadrus SSC/2	2	3.11479	Chromosome	1	46,563	22	63.8
Coprococcus catus GD/7	7	3.5227	Chromosome	1	92,485	12	59.3
Clostridium sp. L2-50	1, 8	2.95462	Scaffold	20	197,148	6	0.5
Coprococcus sp. ART55/1	2, 8	3.12301	Chromosome	1	15,489	53	235.0
[*Eubacterium*] *rectale* DSM 17629 (A1-86)	1	3.34495	Chromosome	1	80,495	13	85.9
[*Eubacterium*] *rectale* M104/1	2	3.69842	Chromosome	1	62,843	18	131.8
Roseburia faecis M72/1	2	3.33469	Contig	101	143,514	7	0
Roseburia intestinalis L1-82	1, 9	4.49335	Complete genome				0
Roseburia intestinalis M50/1	2	4.14355	Chromosome	1	57,879	22	82.9
Roseburia inulinivorans DSM 16841 (A2-194)	1	4.04846	Contig	179	57,343	23	0
Bifidobacterium bifidum CNCM I-3650	This study						
Lactobacillus paracasei CNCM I-1518	10						
Streptococcus thermophilus CNCM I-3862	This study						

[1]The names of the organisms are given as they are recorded on the NCBI Taxonomy website (https://www.ncbi.nlm.nih.gov/Taxonomy/taxonomyhome.html/).

[2]Based on subtraction of ‘total ungapped length’ from ‘total sequence length’ provided at https://www.ncbi.nlm.nih.gov/genome/microbes/.

References

1. Barcenilla A, Pryde SE, Martin JC, Duncan SH, Stewart CS, Henderson C, Flint HJ. 2000. Phylogenetic relationships of butyrate-producing bacteria from the human gut. Appl Environ Microbiol 66:1654–1661.
2. Louis P, Duncan SH, McCrae SI, Millar J, Jackson MS, Flint HJ. 2004. Restricted distribution of the butyrate kinase pathway among butyrate-producing bacteria from the human colon. J Bacteriol 186:2099-2106.
3. Holmstrøm K, Collins MD, Møller T, Falsen E, Lawson PA. 2004. *Subdoligranulum variabile* gen. nov., sp.nov. from human feces. Anaerobe 10:197–203.
4. Holdeman LV, Moore WEC. 1974. New genus, *Coprococcus*, twelve new species, and emended descriptions of four previously described species of bacteria from human feces. Int J Syst Evol Microbiol 24:260-277.
5. Shetty SA, Zuffa S, Bui TPN, Aalvink S, Smidt H, De Vos WM. 2018. Reclassification of *Eubacterium hallii* as *Anaerobutyricum hallii* gen. nov., comb. nov., and description of *Anaerobutyricum soehngenii* sp. nov., a butyrate and propionate-producing bacterium from infant faeces. Int J Syst Evol Microbiol 68:3741-3746.
6. Schwiertz A, Hold GL, Duncan SH, Gruhl B, Collins MD, Lawson PA, Flint HJ, Blaut M. 2002. *Anaerostipes caccae* gen. nov., sp. nov., a new saccharolytic, acetate-utilising, butyrate-producing bacterium from human faeces. Syst Appl Microbiol 25:46–51.
7. Reichardt N, Duncan SH, Young P, Belenguer A, McWilliam Leitch C, Scott KP, Flint HJ, Louis P. 2014. Phylogenetic distribution of three pathways for propionate production within the human gut microbiota. ISME J 8:1323-1335.

8. Alessi AM, Gray V, Farquharson FM, Flores-Lopez A, Shaw S, Stead D, Wegmann U, Shearman C, Gasson M, Collie-Duguid ESR, Flint HR, Louis P. 2020. β-glucan is a major growth substrate for human gut bacteria related to *Coprococcus eutactus*. Environ Microbiol 22: 2150-2164.

9. Duncan SH, Hold GL, Barcenilla A, Stewart CS, Flint HJ. 2002. *Roseburia intestinalis* sp. nov., a novel saccharolytic, butyrate-producing bacterium from human faeces. Int J Syst Evol Microbiol 52:1615–1620.

10. Fehlbaum S, Chassard C, Schwab C, Voolaid M, Fourmestraux C, Derrien M, Lacroix C. 2019. In vitro study of Lactobacillus paracasei CNCM I-1518 in healthy and Clostridioides difficile colonized elderly gut microbiota. Front Nutr 6:184.
