# Supplementary material for: Vitamin Biosynthesis by Human Gut Butyrate-Producing Bacteria and Cross-Feeding in Synthetic Microbial Communities
Source: mBio. 2020 Jul 14;11(4):e00886-20. doi: 10.1128/mBio.00886-20 (PMC7360928; doi:10.1128/mBio.00886-20)
Supplement: TABLE S5 [file mBio.00886-20-st005.pdf]

**Table S5** Final concentrations of ingredients in chemically defined media (CAH-CDM and AA-CDM)[1]

| | | CAH - CDM | AA – CDM | Manufacturer |
|---|---|---|---|---|
| | | g/L | g/L | |
| Carbohydrate | D-(+)-Cellobiose | 2 | 2 | Millipore |
| Carbohydrate | D-(+)-Galactose | 2 | 2 | Sigma-Aldrich |
| Carbohydrate | D-(+)-Glucose | 2 | 2 | Sigma-Aldrich |
| Carbohydrate | D-(-)-Fructose | 2 | 2 | Sigma-Aldrich |
| Carbohydrate | D-(+)-Maltose * $H_2O$ | 2.11 | 2.11 | Sigma-Aldrich |
| Mineral | $CaCl_2$ | 0.09 | 0.09 | Sigma-Aldrich |
| Mineral | $KHCO_3$ | 4 | 4 | Sigma-Aldrich |
| Mineral | $K_2HPO_4$ | 0.45 | 0.45 | Fisher Scientific |
| Mineral | $KH_2PO_4$ | 0.45 | 0.45 | Riedel-de Haën |
| Mineral | $MgSO_4$ * $7H_2O$ | 0.09 | 0.09 | EMD Millipore |
| Mineral | NaCl | 0.9 | 0.9 | Fisher Scientific |
| Mineral | $(NH_4)_2SO_4$ | 0.9 | 0.9 | Sigma-Aldrich |
| Nucleotide | Adenine | 0.01 | 0.01 | Sigma-Aldrich |
| Nucleotide | Cytosine | 0.01 | 0.01 | Sigma-Aldrich |
| Nucleotide | Guanine | 0.01 | 0.01 | Sigma-Aldrich |
| Nucleotide | Orotic acid | 0.005 | 0.005 | Sigma-Aldrich |
| Nucleotide | Thymine | 0.01 | 0.01 | Sigma-Aldrich |
| Nucleotide | Thymidine | 0.005 | 0.005 | Sigma |
| Nucleotide | Uracil | 0.01 | 0.01 | Sigma-Aldrich |
| Nucleotide | Xanthine | 0.01 | 0.01 | Sigma-Aldrich |
| Organic source of Fe | Haemin | 0.01 | 0.01 | Sigma-Aldrich |
| SCFA | Acetic acid | 33 mM | 33 mM | Fisher Scientific |
| SCFA | iso-Butyric acid | 1 mM | 1 mM | Sigma-Aldrich |
| SCFA | iso-Valeric acid | 1 mM | 1 mM | Sigma-Aldrich |
| SCFA | Propionic acid | 9 mM | 9 mM | Sigma-Aldrich |
| SCFA | Valeric acid | 1 mM | 1 mM | Sigma-Aldrich |
| Redox indicator | Resazurin sodium salt | 0.001 | 0.001 | Sigma-Aldrich |
| Trace element | $CoCl_2$ * $6H_2O$ | 0.00025 | 0.00025 | Sigma-Aldrich |
| Trace element | $CuCl_2$ * $2H_2O$ | 0.00001 | 0.00001 | Sigma |
| Trace element | $FeS0_4$ * $7H_2O$ | 0.00210 | 0.00210 | BDH |
| Trace element | $H_3BO_3$ | 0.00050 | 0.00050 | Sigma-Aldrich |
| Trace element | $MnCl_2$ * $4H_2O$ | 0.00007 | 0.00007 | Sigma-Aldrich |
| Trace element | $Na_2MoO_4$ * $2H_2O$ | 0.00005 | 0.00005 | Sigma |
| Trace element | $Na_2SeO_3$ * $5H_20$ | 0.00001 | 0.00001 | Sigma-Aldrich |
| Trace element | $Na_2WO_4$ * $2H_2O$ | 0.00001 | 0.00001 | Sigma-Aldrich |
| Trace element | $NiCl_2$ * $6H_2O$ | 0.00001 | 0.00001 | Sigma |
| Trace element | $ZnSO_4$ * $7H_2O$ | 0.00018 | 0.00018 | Sigma-Aldrich |

| | | | | |
|---|---|---|---|---|
| Vitamin[2] | Biotin | 0.00002 | 0.00002 | Supelco |
| Vitamin[2] | Pimelic acid | 0.00002 | 0.00002 | Sigma-Aldrich |
| Vitamin[2] | Cyanocobalamin | 0.00001 | 0.00001 | Sigma-Aldrich |
| Vitamin[2] | Folic acid | 0.00005 | 0.00005 | Sigma-Aldrich |
| Vitamin[2] | *p*-Aminobenzoic acid | 0.00005 | 0.00005 | Sigma-Aldrich |
| Vitamin[2] | ±-α-Lipoic acid | 0.00005 | 0.00005 | Sigma-Aldrich |
| Vitamin | Menadione | 0.00001 | 0.00001 | Supelco |
| Vitamin | Menaquinone-4 | 0.00001 | 0.00001 | Supelco |
| Vitamin[2] | Nicotinic acid | 0.00005 | 0.00005 | Sigma-Aldrich |
| Vitamin[2] | Nicotinamide | 0.00005 | 0.00005 | Sigma-Aldrich |
| Vitamin[2] | Calcium D-Pantothenate | 0.00005 | 0.00005 | Sigma-Aldrich |
| Vitamin[2] | D-Pantethine | 0.00005 | 0.00005 | Supelco |
| Vitamin[2] | Pyridoxine HCl | 0.00010 | 0.00010 | Sigma-Aldrich |
| Vitamin[2] | Pyridoxal HCl | 0.00010 | 0.00010 | Sigma-Aldrich |
| Vitamin[2] | Pyridoxamine:2HCl | 0.00010 | 0.00010 | Supelco |
| Vitamin[2] | (-)-Riboflavin | 0.00005 | 0.00005 | Sigma-Aldrich |
| Vitamin[2] | Thiamine HCl | 0.00005 | 0.00005 | Sigma-Aldrich |
| AA[3] + Peptides | Vitamin-free casein hydrolysate | 10 | | Millipore |
| AA | L-Alanine | | 0.62 | Sigma-Aldrich |
| AA | L-Arginine | | 0.73 | Sigma-Aldrich |
| AA | L-Asparagine | 0.2 | 0.41 | Sigma-Aldrich |
| AA | L-Aspartic acid | | 0.54 | Sigma-Aldrich |
| AA | L-Glutamine | 0.2 | 0.42 | Sigma-Aldrich |
| AA | L-Glutamic acid | | 0.71 | Sigma-Aldrich |
| AA | Glycine | | 0.41 | Sigma-Aldrich |
| AA | L-Histidine | | 0.25 | Sigma-Aldrich |
| AA | L-Isoleucine | | 0.67 | Sigma-Aldrich |
| AA | L-Leucine | | 1.04 | Sigma-Aldrich |
| AA | L-Lysine | | 0.64 | Sigma-Aldrich |
| AA | L-Methionine | | 0.29 | Sigma-Aldrich |
| AA | L-Phenylalanine | | 0.53 | Sigma-Aldrich |
| AA | L-Proline | | 0.38 | Sigma-Aldrich |
| AA | L-Serine | 0.2 | 0.49 | Sigma-Aldrich |
| AA | L-Threonine | 0.2 | 0.48 | Sigma-Aldrich |
| AA | L-Tryptophan | 0.2 | 0.19 | Sigma-Aldrich |
| AA | L-Tyrosine | | 0.43 | Sigma-Aldrich |
| AA | L-Valine | | 0.63 | Sigma-Aldrich |
| AA | L- Cysteine HCl | 1 | 1 | Sigma-Aldrich |

[1]Solutions of different nucleotides were based on the recipe described in Heinken et al. (1), trace elements based on trace element solution SLA of Imhoff and Trüper (2) and vitamins were added to compensate for the lack of yeast extract in CDM. The amount of AA present in the free amino acid medium (AA–CDM), except L-Cys, is based on the δ index weighed - AA relative contents of bacteria described by Moura *et al*. (3). The L-cysteine (L-Cys) concentration, as also used to reduce the medium, remained the same as in YCFA. Amino acids were added before the medium was autoclaved. The target pH for all media was 6.5, with a pH range of 6.5±0.1 for vitamin auxotrophy experiments and 6.6±0.2 for amino acid auxotrophy experiments after gas phase equilibration in the anaerobic cabinet.

[2]The amounts of vitamins used in CDM were based on those reported in YCFA (4) and extra compounds based on empirical information regarding synthesis salvage pathways (5) were added as follows. Biotin (B7): Pimelic acid; Cobalamin (B12): Cyanocobalamin; Folate (B9): Folic acid, *p*-Aminobenzoic acid; Niacin (B3): Nicotinic acid, Nicotinamide; Pantothenate (B5): Pantethine; Pyridoxine (B6): Pyridoxal HCl, Pyridoxamine:2HCl. All vitamins were filter sterilised (0.22 µm filter) and added after the medium was autoclaved.

**REFERENCES**

1. Heinken A, Khan MT, Paglia G, Rodionov DA, Harmsen HJ, Thiele I. 2014. Functional metabolic map of *Faecalibacterium prausnitzii*, a beneficial human gut microbe. J Bacteriol 196:3289-3302.
2. Imhoff JF, Trüper HG. 1977. *Ectothiorhodospira halochloris* sp. nov., a new extremely halophilic phototrophic bacterium containing bacteriochlorophyll b. Arch Microbiol 114:115-121.
3. Moura A, Savageau MA, Alves R. 2013. Relative amino acid composition signatures of organisms and environments. PLOS One 8:e77319.
4. Duncan SH, Hold GL, Harmsen HJM, Stewart CS, Flint HJ. 2002. Growth requirements and fermentation products of *Fusobacterium prausnitzii*, and a proposal to reclassify it as *Faecalibacterium prausnitzii* gen. nov., comb. nov. Int J Syst Evol Microbiol 52:2141–2146.

5. Magnúsdóttir S, Ravcheev D, de Crécy-Lagard V, Thiele I. 2015. Systematic genome assessment of B-vitamin biosynthesis suggests co-operation among gut microbes. Front Genet 6:148.
