# Supplementary material for: Vitamin Biosynthesis by Human Gut Butyrate-Producing Bacteria and Cross-Feeding in Synthetic Microbial Communities
Source: mBio. 2020 Jul 14;11(4):e00886-20. doi: 10.1128/mBio.00886-20 (PMC7360928; doi:10.1128/mBio.00886-20)
Supplement: TABLE S6 [file mBio.00886-20-st006.pdf]

Table S6 Primers used for molecular community analysis

Target	Primer name	Primer sequence (universal tag sequence in bold)	amplicon size
Universal tag primers	TAG-for	Cyanine5-AGGTGACACTATAGAATA	
	TAG-rev	GTACGACTCACTATAGGGA	
Corynebacterium glutamicum DSMZ 1412	tag-CGTerC_F	**AGGTGACACTATAGAATA**CCACTGAGGGCGATCTTGTT	183
	tag-CGTerC_R	**GTACGACTCACTATAGGGA**AATGTGCTTGGCTCCGAAGA	
Faecalibacterium prausnitzii A2-165	tag_Fprau_CG447_RS01330F	**AGGTGACACTATAGAATA**CCAGCCCCAGCATCTCTCTG	135
	tag_Fprau_CG447_RS01330R	**GTACGACTCACTATAGGGA**GGATACCGAGGGAAGAGTGGAC	
Subdoligranulum variabile DSM 15176	Svar_SUBVAR_05219F	**AGGTGACACTATAGAATA**TGGAACCGATCTCCTGCTTG	170
	Svar_SUBVAR_05219R	**GTACGACTCACTATAGGGA**AGCAATGAGCTGTCCGATGA	
Eubacterium rectale A1-86 (=DSM 17629)	tag_Erect_EUR_16390F	**AGGTGACACTATAGAATA**ATAGCCCTCAGGCACCACTA	147
	tag_Erect_EUR_16390R	**GTACGACTCACTATAGGGA**TGGCCTTTAAGGTGGCAACA	
Roseburia intestinalis M50/1	Rint_ROI_11740F	**AGGTGACACTATAGAATA**AATCGCAATGAATCGCCTGC	254
	Rint_ROI_11740R	**GTACGACTCACTATAGGGA**AAGATGTCCGCCTTCACCAG	
Roseburia faecis M72/1	tag-RFTerC_F_1	**AGGTGACACTATAGAATA**AATCAGTTCCGCTCCCTTGG	339
	tag-RFTerC_R_1	**GTACGACTCACTATAGGGA**CCATTGGTTCCGGTGTCGTA	
Roseburia inulinivorans A2-194	tag_Rinul_ROSEINA2194_04328F	**AGGTGACACTATAGAATA**CGCAGCAGATTTTACAGCGT	275
	tag_Rinul_ROSEINA2194_04328R	**GTACGACTCACTATAGGGA**AACCTCATGCCTTGCAATCG	
Coprococcus eutactus ART55/1	tag-CETerC_F	**AGGTGACACTATAGAATA**GAAGGAAAACCAGCTCGTGA	165
	tag-CETerC_R	**GTACGACTCACTATAGGGA**CCCTCTGCTGGAACTTCAAC	
Streptococcus thermophilus CNCM I-3862	tag_S.thermo - F	**AGGTGACACTATAGAATA**TTATTTGAAAGGGGCAATTGCT	318
	tag_S.thermo - R	**GTACGACTCACTATAGGGA**GTGAACTTTCCACTCTCACAC	
Lactobacillus paracasei CNCM I-1518	tag_OFF 2201 ex	**AGGTGACACTATAGAATA**IIGTTAGCACCGCTTAAAGACG	322
	tag_OFF 2202 ex	**GTACGACTCACTATAGGGA**IIGCCATAAGCGTGTTAGCCG	
Bifidobacterium bifidum CNCM I-3650	tag_GeXP_Bif_genus_F1	**AGGTGACACTATAGAATA**TCGCGTCYGGTGTGAAAG	165
	tag_GeXP_Bif_genus_Rev	**GTACGACTCACTATAGGGA**GGTGTTCTTCCCGATATCTACA	
